# Computational and experimental demonstrations of one-pot tandem catalysis for electrochemical carbon dioxide reduction to methane

**DOI:** 10.1038/s41467-019-11292-9

**Published:** 2019-07-26

**Authors:** Haochen Zhang, Xiaoxia Chang, Jingguang G. Chen, William A. Goddard, Bingjun Xu, Mu-Jeng Cheng, Qi Lu

**Affiliations:** 1 State Key Laboratory of Chemical Engineering, Department of Chemical Engineering, Tsinghua University, 100084 Beijing, China; 2 Center for Catalytic Science and Technology, Department of Chemical and Biomolecular Engineering, University of Delaware, Newark, DE 19716 USA; 3 Department of Chemical Engineering, Columbia University, New York, NY 10027 USA; 4 Materials and Process Simulation Center, California Institute of Technology, Pasadena, CA 91125 USA; 5 Department of Chemistry, National Cheng Kung University, Tainan, 701 Taiwan

**Keywords:** Electrocatalysis, Electrochemistry

## Abstract

Electroreduction of carbon dioxide to hydrocarbons and oxygenates on copper involves reduction to a carbon monoxide adsorbate followed by further transformation to hydrocarbons and oxygenates. Simultaneous improvement of these processes over a single reactive site is challenging due to the linear scaling relationship of the binding strength of key intermediates. Herein, we report improved electroreduction of carbon dioxide by exploiting a one-pot tandem catalysis mechanism based on computational and electrochemical investigations. By constructing a well-defined copper-modified silver surface, adsorbed carbon monoxide generated on the silver sites is proposed to migrate to surface copper sites for the subsequent reduction to methane, which is consistent with insights gained from operando attenuated total reflectance surface enhanced infrared absorption spectroscopic investigations. Our results provide a promising approach for designing carbon dioxide electroreduction catalysts to enable one-pot reduction of products beyond carbon monoxide and formate.

## Abstract (web summary)

Carbon dioxide can be electrocatalytically reduced to valuable fuels and chemicals, but is hindered by poor catalytic efficiency and selectivity. Here the authors report improved electrocatalytic conversion of carbon dioxide into methane using a tandem catalysis strategy.

## Introduction

The electrochemical reduction of CO_2_ to energy-dense chemicals is an attractive strategy for storing the intermittent renewable electricity produced by solar and wind sources^1–3^. To ensure sustainability of the entire process, the electrochemical CO_2_ reduction reaction (CO_2_RR) is typically conducted in an aqueous electrolyte, in which the protons required are obtained. In this system, tremendous progress has been made in catalyst^4–6^ and reactor design^7,8^ to drive the two-electron reduction of CO_2_ to produce CO or formate. However, strategies for direct reduction of CO_2_ to more valuable fuels and chemicals have been less successful because the catalysts capable of catalyzing this conversion are very limited. Only Cu exhibits appreciable activity and Faradaic efficiency (FE) for reducing CO_2_ to hydrocarbons and oxygenates^9–11^. The lack of predictive catalyst design principles for CO_2_RR limits the development of catalysts capable of directly converting CO_2_ to products beyond CO and formate

To achieve higher efficiencies in the CO_2_RR toward hydrocarbons and oxygenates, the most common approach involves modifying the Cu surface to produce and/or enrich active sites with a specific structure. These efforts include oxidation and reduction treatment to expose grain boundary-terminated Cu surfaces^12,13^, plasma treatment^14,15^, and electro-redeposition^16^ to introduce stable Cu^+^ species, morphology control to expose high density low-coordinated surface sites^17,18^, and alloying with an additional metal to tune the binding strength to the reaction intermediates^19–22^. Despite the recent progress, the improvement in the performance as compared to the pure Cu remains unsatisfactory. In particular, the selectivity of alloy catalysts toward products beyond CO and formate do not surpass that of pure Cu^10,11^. Therefore, novel approaches to design more efficient CO_2_RR catalysts capable of selectively producing valuable products are highly desirable.

On polycrystalline Cu surfaces, CO_2_ is first converted to adsorbed CO adsorbate (*CO) followed by its further reduction to hydrocarbons and oxygenates. However, the optimal sites for these two processes could have quite different properties because the formation of *CO requires the optimal binding strength for *COOH^23–25^ while the formation of hydrocarbons and oxygenates requires the optimal binding strength for *CO^25–27^. The simultaneous optimization of the binding strength of *COOH and *CO on one type of reactive site can be challenging due to the linear scaling relationship^26^. For example, the Au_3_Cu nanoparticle catalyst exhibited a very high activity for reducing CO_2_ to CO. However, this catalyst was nearly incapable of producing further reduced products^19^. An oxide-derived Cu catalyst exhibited a much improved FE for reducing CO to alcohols. However, this catalyst cannot effectively and directly reduce CO_2_ to products beyond CO and formate^12^.

Therefore, the conversion of CO_2_ using tandem catalysis can be a promising strategy to improve the overall efficiencies for further reduced products. By co-locating Cu with a CO-producing surface (e.g., Au and Ag), the Cu may be supplied with abundant CO via spillover. A higher coverage of CO on the Cu surface can not only increase the rates of hydrocarbon and oxygenate production^28–30^ but also suppress the competing hydrogen evolution reaction (HER) by weakening the binding strength of *H*_ads_^31^. Recently, several bimetallic surfaces including Cu-Zn^20^ and Cu-Au^22^ were investigated in the CO_2_RR. The Cu-Zn surface exhibited improved selectivity for ethanol over ethylene, and the Cu-Au surface exhibited an improved partial current density toward reduction products beyond CO from approximately 0.005 to 0.16 mA cm^−2^ compared to a bare Cu foil at a low overpotential. A tandem catalysis mechanism was proposed to explain these improvements. However, CO spillover was not demonstrated in these systems. Therefore, the origin of the observed synergetic improvements remains unclear. Another recent study demonstrated that a Cu-Ag bimetallic surface can be more selective for C_2+_ product formation^21^. However, this improvement was not due to CO spillover but suppression of the HER from the formation of the compressively strained alloy surface. Herein we report a combined computational and experimental study of one-pot tandem catalytic CO_2_RR. By constructing well-defined model surfaces with isolated thin Cu layers on a CO-producing substrate (i.e., Au and Ag), the CO produced on the CO-producing Au or Ag would migrate to Cu with a low activation energy and be further reduced by Cu. In comparison to pure Cu, our model surface exhibited better CH_4_ selectivity and activity as well as suppressed HER^10,11^. Operando attenuated total reflectance surface enhanced infrared absorption spectroscopic (ATR-SEIRAS) investigations yielded the first spectroscopic evidence of CO spillover on a Cu-based bimetallic catalyst. More importantly, our results clearly demonstrate a new paradigm for the design of CO_2_RR catalysts to achieve further reduced products beyond CO and formate.

## Results

### Theoretical investigations of carbon monoxide spillover

**Fig. 1:**
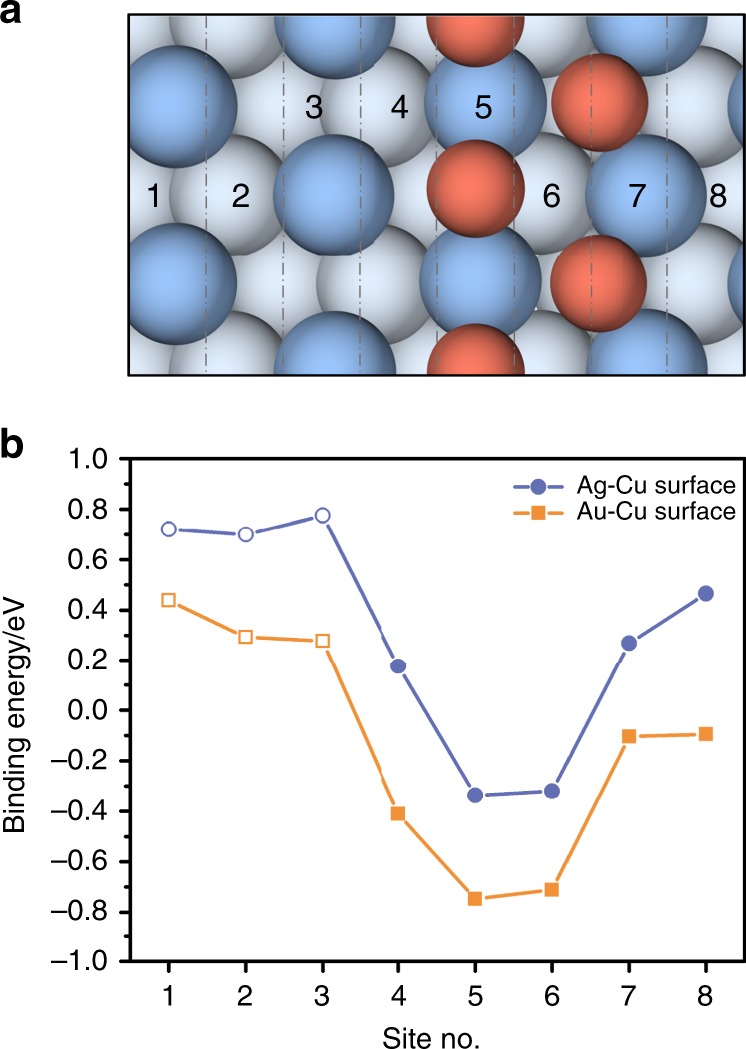
Schematic diagram. The computational model is constructed by adding a single layer of Cu with a coverage of 1/2 ML (monolayer) on top of the three-layer Ag or Au substrate. The surface CO adsorption is under −1.0 V_SHE_. The substrate orientation is chosen to be (111) because it is the most stable and abundant surface facet for bulk Ag or Au materials. A clean surface, CO_2(*g*)_, H_2_O_(*l*)_ and H_2(*g*)_ are used as references to construct the free energy surfaces. **a** Top view of the unit cell used for computational investigations (blue: top layer Ag or Au atoms; light blue: bottom layer Ag or Au atoms; orange: Cu atoms). The site numbers show the position and chemical environment of the binding sites. **b** Energy diagram for CO adsorption on the Ag-Cu surface (blue line) and Au-Cu surface (orange line). Data of sites nonadjacent to the surface Cu are shown in hollow bullets

Ag and Au are known to be the most efficient monometallic surfaces for CO production from the CO_2_RR^4,5^. Our computational investigation begins with examination of the possibility for CO spillover from a CO-producing Ag or Au site to a surface Cu site prior to further reduction. The surface is divided into eight regions along the direction from bare Ag (or Au) sites toward surface Cu sites for the discussion of CO spillover, and only the Δ*G*_CO_ of sites with the strongest binding energy in each region on the Cu-added Ag or Au surface are considered in the CO spillover discussion (Fig. 1a and Supplementary Table 1). As shown in Fig. 1b, *CO adsorption is typically more stable on surface Cu sites than Ag or Au sites. The ΔG_CO_ at bare substrate sites nonadjacent to Cu (i.e., site nos. 1–3) exhibit similar values (i.e., ~0.73 eV for Ag and ~0.34 eV for Au, respectively). As the CO molecule approaches the surface Cu, Δ*G*_CO_ decreases substantially (stronger adsorption) and reaches a minimum value at site no. 5 on Ag (−0.34 eV) and Au (−0.75 eV) where CO forms a bond with the surface Cu atoms. Significantly better CO adsorption is observed on surface Cu than its substrate for both Ag (1.07 eV) and Au (1.09 eV). Moreover, the free energy barrier for CO spillover on both surfaces is calculated to be very small (no more than 0.16 eV), which can be easily surmounted at room temperature (Supplementary Table 2) and is consistent with previous works^32,33^. Thus our computational results indicate that CO spillover from the Ag or Au substrate to the surface Cu is thermodynamically and kinetically feasible. The one-pot tandem reduction of CO_2_ may be viable by converting CO_2_ to CO on a CO-producing substrate followed by CO spillover and further reduction on a surface Cu site.

### Theoretical investigations of carbon monoxide reduction after spillover

**Fig. 2:**
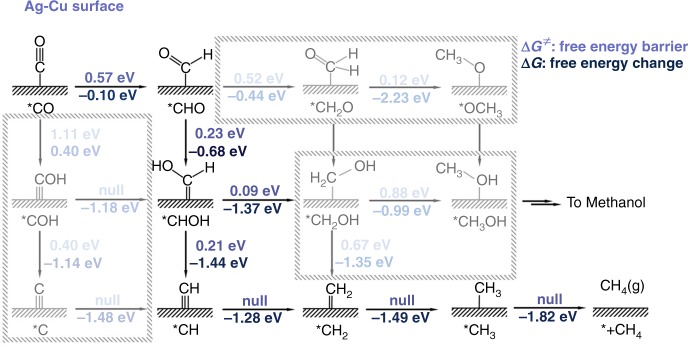
Kinetics and free energy diagram for all possible pathways toward C_1_ products. CO is reduced to C_1_ products on the Ag-Cu surface. The values shown blue (upper) and dark blue (bottom) are the free energy barrier and free energy change at −1.0 V_SHE_ for all steps, respectively. Adsorbates with an asterisk correspond to species that are adsorbed on the surface. Null stands for no free energy barrier

The further reduction of CO on surface Cu after spillover is investigated. The conversion of CO to C_1_ products is chosen as the model reaction process for this investigation because (a) this process is less controversial and the results can be supported by previous work^34–37^, and (b) other processes require C-C coupling via mechanisms that are unclear and currently under debate^38–40^. The kinetics and thermodynamics for all possible pathways toward C_1_ products are calculated (Fig. 2, Supplementary Fig. 1 and Supplementary Table 3). Different from traditional calculations with a fixed electron number, the number of electrons in each calculation is adjusted to maintain a potential of −1.0 V_SHE_, which is more representative of experimental reaction conditions. The most energetically favored pathway toward CH_4_ at −1.0 V_SHE_ is determined to be *CO → *CHO → *CHOH → *CH → *CH_2_ → *CH_3_ → * + CH_4_ on both the Ag-Cu and Au-Cu surfaces (Fig. 2 and Supplementary Fig. 1). The surface Cu exhibits the ability to reduce CO to CH_4_ at −1.0 V_SHE_. As suggested by the results in Fig. 2 and Supplementary Fig. 1, the hydrogenation of *CO to *CHO, which is the most difficult reaction among all the reaction steps, exhibits the highest free energy barrier (Δ*G*^≠^) with a value of 0.57 and 0.50 eV for the Ag-Cu and Au-Cu surfaces, respectively. All reactions along the pathway at a potential bias of −1.0 V_SHE_ are thermodynamically downhill and kinetically feasible with Δ*G*^≠^ values being <0.75 eV, a number leading to a turnover frequency of approximately 1 s^−1^ at room temperature based on the transition state theory^34,35^. The hydrogenation of *CH, *CH_2_, and *CH_3_ exhibits no free energy barrier. Similar results were also reported by Chan et al. in the study of CO_2_ reduction on stepped copper^41,42^. This result indicates that CO reduction by the surface Cu on Ag or Au will be kinetically feasible. In contrast, pathways toward other possible C_1_ product methanol are kinetically unfavorable, although they are thermodynamically feasible. Accordingly, both the thermodynamics and kinetics indicate that the surface Cu on Ag or Au can reduce CO_2_ to CH_4_ in a one-pot tandem fashion.

### Ag-Cu model surface for electrochemical study

**Fig. 3:**
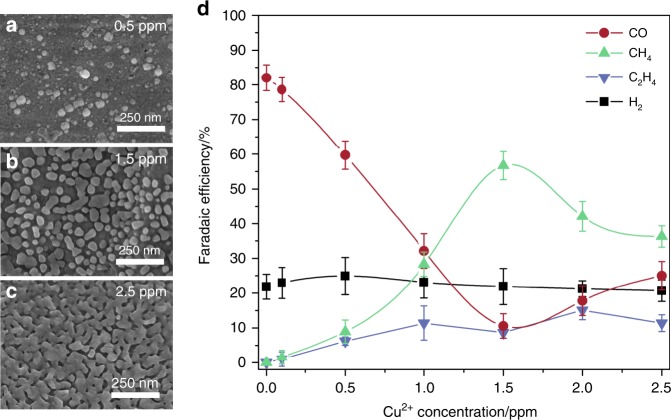
Silver–copper model catalyst surface with different copper coverages. Scanning electron microscopic images of Ag-Cu surfaces achieved at Cu^2+^ concentrations of 0.5 ppm (**a**), 1.5 ppm (**b**) and 2.5 ppm (**c**). **d** Faradaic efficiencies of Ag-Cu surfaces with different Cu coverages for CO_2_ electrolysis at −1.10 V_RHE_. The error bars represent the standard deviation from at least three independent measurements

The Ag-Cu surface rather than the Au-Cu surface is chosen as the model catalyst for electrochemical investigations due to the cost-effectiveness of Ag over Au. The Cu-modified Ag surface is prepared at the beginning of CO_2_ electrolysis by conducting the reaction in a bicarbonate electrolyte containing a predetermined (ppm) level of Cu^2+^. Owing to the reduction potential of Cu^2+^ (Cu^2+^(aq) + 2e^−^ → Cu(s) (+0.16 V_SHE_ for ppm level Cu^2+^)) being significantly more positive than the electroreduction potential of CO_2_ (typically <−1.3 V_SHE_), the ppm-level Cu^2+^ is instantly electrochemically deposited onto the Ag foil when the electrolysis is initiated, making the deposition process indistinguishable during chronoamperometry. This is supported by the observation that the reduction current of Cu^2+^ is indistinguishable in current profiles at all potentials (Supplementary Fig. 2). Further, the surface morphologies at the early stage and at the conclusion of the electrolysis are similar (Supplementary Fig. 3). The deposited Cu form islands that are a few tens of nanometers in size on the Ag surface (Fig. 3a–c), which is consistent with previous results using a similar technique^43^. The coverage of surface Cu can be tuned by controlling the initial Cu^2+^ concentration in the electrolyte and is characterized using scanning electron microscopy (SEM) and X-ray photoelectron spectroscopy (XPS) (Fig. 3a–c, Table 1, Supplementary Fig. 4). The highly crystalline nature of surface Cu is confirmed by high-resolution transmission electron microscope (HR-TEM) with samples prepared using focused ion beam technique (Supplementary Fig. 5).

**Table 1:** Surface Cu coverage with different Cu^2+^ concentrations in the electrolyte

Cu^2+^ concentration/ppm	0.5	1.0	1.5	2.0	2.5
Surface Cu coverage	2.7%	32.3%	50.2%	70.0%	85.3%
Surface Ag coverage	97.3%	67.7%	49.8%	30.0%	14.7%

**Fig. 4:**
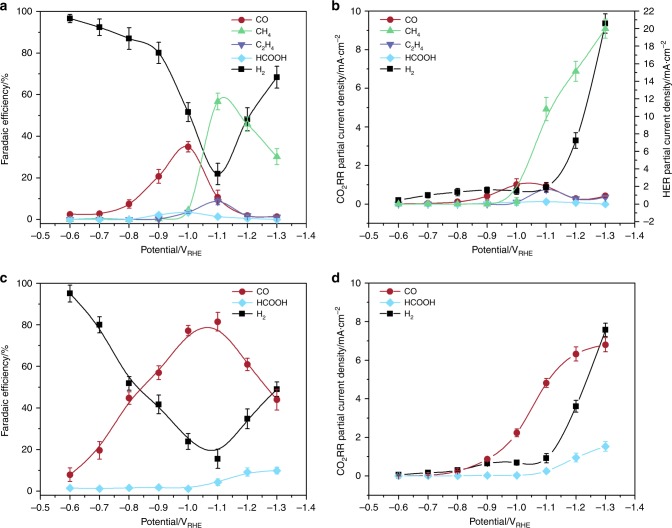
Carbon dioxide electrolysis on the silver–copper and bare silver surfaces. Faradaic efficiencies (**a**) and partial current densities (**b**) of CO_2_ electrolysis products on the Ag-Cu surface achieved at a Cu^2+^ concentration of 1.5 ppm. Faradaic efficiencies (**c**) and partial current densities (**d**) of CO_2_ electrolysis products on a bare polycrystalline Ag surface. The error bars represent the standard deviation from at least three independent measurements

The CO_2_ electrolysis study of these Ag-Cu surfaces is conducted at −1.1 V_RHE_ because Ag foil exhibits the highest FE for CO production at this potential (Fig. 4c)^23^. The distribution of the major products is shown in Fig. 3d. As the Cu^2+^ concentration increases from 0 to 1.5 ppm, which corresponds to a Cu coverage increases from 0% to 50.2% (Table 1), the CH_4_ FE increases substantially from 0% to approximately 60%, and the CO FE decreases concomitantly from >80% to approximately 10% (Fig. 3d). The CH_4_ FE achieved on this partially Cu-covered Ag-Cu surface (i.e., Cu coverage of 50.2%) is much higher that on a bare polycrystalline Cu foil at the same potential (Supplementary Fig. 6)^11^. This result indicates that CH_4_ production on Cu can be efficiently improved via the prior reduction of CO_2_ to CO on a nearby Ag surface. Because a further increase in the Cu coverage decreases the CH_4_ FE, the essential role of Ag surface exposure is to provide sufficient CO supply to achieve a high CH_4_ FE. At a Cu^2+^ concentration of 2.5 ppm, the Cu coverage reaches 85.3%, and the CH_4_ FE value decreases to approximately 35%, which is consistent with results obtained on a polycrystalline Cu foil at the same potential (Supplementary Fig. 6)^11^. The Ag-Cu surfaces are not very selective to C_2_H_4_, which is most likely due to the preferential adsorption of those *CO that migrates from the Ag surface on the edge of Cu islands where the reduced dimension may promote the exposure of low-coordinated surface sites. These low-coordinated sites may bind *CO too strongly that prevent the further movement of *CO for dimerization.

### Potential dependence study at the silver–copper surface

The Ag-Cu surface with the optimal Cu coverage achieved at a Cu^2+^ concentration of 1.5 ppm is employed to further investigate the one-pot tandem catalysis in the CO_2_RR. The potential range for the electrolysis experiment is −0.6 V_RHE_ to −1.3 V_RHE_ (equivalent to −1.0 V_SHE_ to −1.7 V_SHE_) to drive sufficient but not excessive activities. The Ag-Cu surface at these potentials exhibits nearly identical morphologies (Supplementary Fig. 7). This is most likely due to the large deposition overpotentials (>1.16 V) for only ppm-level Cu^2+^ in the electrolyte and the deposition processes are limited by the diffusion of Cu^2+^ rather than the electrode potential. This is supported by the observation in a previous study that the electrodeposition of Cu begins to be diffusion-limited with an overpotential of >300 mV at a Cu^2+^ concentration of 0.15 M^44^. When the overpotential is >800 mV, such deposition process is completely diffusion-limited and the deposited Cu exhibits near identical morphologies^44,45^.

In the −0.6 V_RHE_ to −1.0 V_RHE_ potential range, the Ag-Cu surface exhibits a similar electrocatalytic behavior as that of a bare Ag surface with CO and H_2_ as the major products (Fig. 4). Both the CO and H_2_ partial current densities increase as the potential becomes more negative due to the increased overpotential (Fig. 4b, d). The increase in CO production is more significant than that of H_2_ production, resulting in an increase in CO FE and a decrease in H_2_ FE (Fig. 4a–c). As the potential becomes more negative than −1.0 V_RHE_, the Ag-Cu surface exhibits a substantial increase in the CH_4_ partial current density with a concomitant decrease in the CO partial current density (Fig. 4b). However, for the bare Ag surface, the CO partial current density continuously increases and begins to plateau at −1.1 V_RHE_, which is most likely due to mass transport limitations (Fig. 4d). This result clearly demonstrates that the exposed Ag surface on Ag-Cu behaves very differently from the bare Ag. If the exposed Ag surface merely converts CO_2_ to molecular CO that leaves the Ag-Cu surface, as is the case for the bare Ag surface, the CO partial current density should increase as the potential becomes more negative until mass-transport limitation. In contrast, the CO partial current density of Ag-Cu surface actually decreases at more negative potentials where CH_4_ formation starts to increase (Fig. 4b), indicating that the extra CO produced on exposed Ag sites beyond a potential of −1.0 V_RHE_ is consumed in other processes (i.e., CO spillover). In addition, at −1.0 V_RHE_, the CO partial current density of Ag-Cu (1.0 mA cm^−2^) is approximately 46% of that of the bare Ag surface (2.2 mA cm^−2^). This value is consistent with the 49.8% exposed Ag on the Ag-Cu surface because its CO partial current density is primarily attributed to the exposed Ag due to the very low CO activity of Cu (less than 0.1 mA cm^−2^ at potentials more negative than −1.0 V_RHE_)^11^. As the potential decreases from −1.0 V_RHE_ to −1.1 V_RHE_, the CO partial current density on the bare Ag increases from 2.23 to 4.82 mA cm^−2^ representing a factor of 2.16 increase (Fig. 4d). However, the CO partial current density on the Ag-Cu surface decreases slightly from 1.0 to 0.9 mA cm^−2^ under the same condition (Fig. 4b), assuming that the Ag sites on the Ag-Cu surface would produce more CO with the same factor of 2.16 and the missing portion (i.e., 1.26 mA cm^−2^) is further converted to CH_4_ by the surface Cu. The CH_4_ partial current density can be estimated to be 5.04 mA cm^−2^ (1.26 mA cm^−2^ × 4) since CH_4_ production requires four times as many electrons as CO production. Indeed, this value is consistent with the experimentally measured value (i.e., 4.9 mA cm^−2^). This result indicates that, at the optimal Cu coverage, nearly 60% of the CO produced on Ag is further reduced to CH_4_ on the Cu surface via the tandem process. At more negative potentials, significant HER activity is observed on both surfaces, which results in the decreased FE of CO_2_RR. The drastically increased HER activity on the Ag-Cu surface is most likely due to the rapid HER on surface Cu sites at high potential bias (Supplementary Fig. 6). CO_2_ electrolysis at extended time (2 h) is also conducted on Ag-Cu and bare Ag surfaces (Supplementary Fig. 8). The CH_4_ formation is stable in the first hour with an FE of approximately 60%. After that, the CH_4_ FE gradually increases to 67% at the end of the 2-h electrolysis, which is accompanied by the concomitant decrease of CO and C_2_H_4_ FE. The further increase in CH_4_ FE is likely due to the surface reconstruction of Cu under CO_2_ electroreduction conditions that favors CH_4_ formation^46,47^. Further development of more advanced Ag-Cu catalysts combining Cu and Ag with optimized material structure is a promising approach to achieve better performance in CO_2_RR.

### Operando spectroscopic investigations

**Fig. 5:**
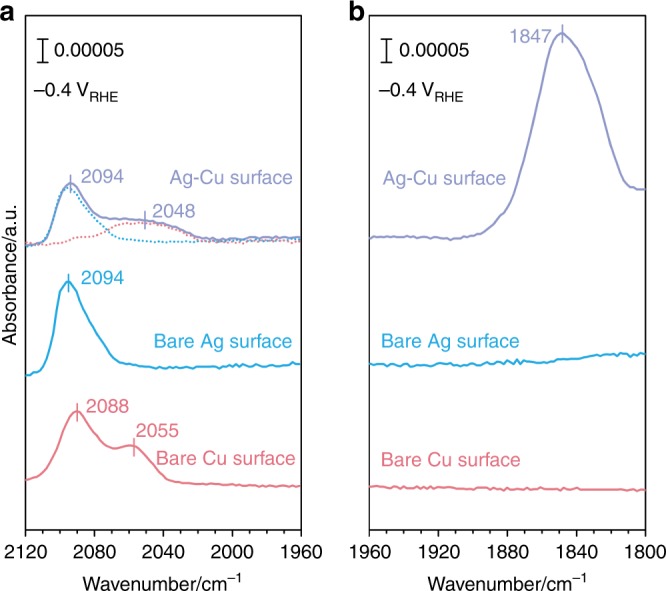
Studies of surface-adsorbed CO on Cu, bare Ag and Ag-Cu films. Operando attenuated total reflectance surface enhanced infrared absorption spectroscopy (ATR-SEIRAS) of surface adsorption. **a** The atop-bonded CO band on different surfaces under −0.4 V_RHE_. **b** The bridge-bonded CO band on Ag-Cu film under −0.4 V_RHE._ The background is collected at 0.1 V_RHE_ under Ar purge

To gain further insights into the CO spillover on the Ag-Cu surface, operando ATR-SEIRAS is employed to monitor the adsorbed CO at conditions closely mimicking those in the reactivity studies. Experiments on bare Ag, bare Cu, and Ag-Cu surface (1.5 ppm Cu^2+^) at −0.4 V_RHE_ are conducted in a custom-designed stirred spectroelectrochemical cell (Supplementary Fig. 9)^48^. The bare Ag surface in the ATR-SEIRAS study is prepared by electrochemical deposition of Ag film in a silver cyanide plating bath on an Au film that is chemically deposited onto the reflecting plane of a Si prism^49,50^. The Ag-Cu surface is then prepared in the same fashion as in the reactivity study, i.e., by conducting the experiments in a Cu^2+^-containing bicarbonate on the Ag film. The bare Cu surface is prepared via the chemical deposition method^51^. The bare Ag surface exhibits a C≡O stretching band at 2094 cm^−1^ (Fig. 5), which is typically attributed to CO molecule bound in an atop geometry^52^. The bare Cu surface shows two stretching bands of atop-bound CO in the 2000–2120 cm^−1^ range, with the 2088 and 2055 cm^−1^ bands being attributed to CO adsorption on defect sites and terraces sites, respectively^51^. This is also consistent with previous studies by Waegele et al. under similar electrolysis conditions^53^. The 2094 cm^−1^ band on the Ag-Cu surface is identical to that of bare Ag surface, as the peak position and width are both identical. The deconvoluted C≡O stretching band on surface Cu has a broad feature centered at 2048 cm^−1^, which is consistent with a previous report^54^. The difference between the CO adsorption feature on the bare Cu and the Ag-Cu surface shows that the underlying Ag exerts an impact on the properties of Cu. Remarkably, the Ag-Cu surface exhibits a band in the 1800–1900 cm^−1^ range, typically assigned to bridge CO with stronger binding strength, which is absent on both bare Ag and Cu surfaces^55,56^. This band is much more intense than the atop CO band, thus is the major CO species on the Ag-Cu surface according to Beer–Lambert law. The spectroscopic observations clearly indicate that the Ag-Cu surface is different from a simple superposition of bare Cu and Ag surfaces. This is consistent with our one-pot tandem catalysis theory that the *CO produced on the exposed Ag sites can migrate to stronger binding sites on the Ag-Cu surface for further reduction. In addition, the exclusive band on the Ag-Cu surface is not due to some new reactive site existing on the Ag-Cu bimetallic interface. If this is the case, the CO produced on the exposed Ag sites on Ag-Cu will not be consumed by further reduction, and the CO production should be promoted at more negatively biased potential until mass-transport limitation, similar as the case of bare Ag foil. This contradicts the experimental results shown in Fig. 4b, d.

### Constant and square-wave potential electrolysis of CO on the Ag-Cu surface

**Fig. 6:**
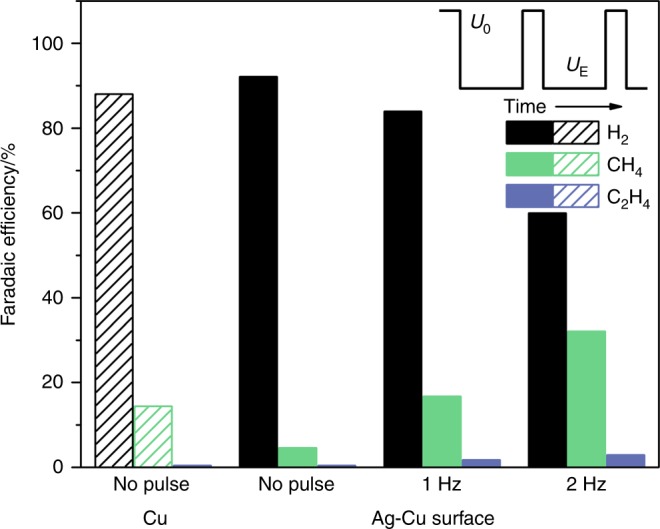
Faradaic efficiencies of major carbon monoxide electroreduction products. Constant potential electrolysis at −1.1 V_RHE_ is conducted on the bare Cu and Ag-Cu surfaces. Square-wave potential electrolysis alternating between 0.4 V_RHE_ (*U*_0_) and −1.1 V_RHE_ (*U*_E_) is conducted on the Ag-Cu surface. Inset: schematic representation of the square-wave potential profile employed in the electrolysis

To further probe CH_4_ formation over Ag-Cu surface with respect to local *CO concentration, the electrochemical CO reduction reaction is conducted at −1.1 V_RHE_ using the same bicarbonate electrolyte, and the results are compared to those obtained using a bare Cu foil. At constant potential, the Ag-Cu surface produces fewer hydrocarbons than the bare Cu foil in CO electrolysis (Fig. 6). This result can be rationalized that Ag is not active in CO electroreduction, thus fewer active Cu sites are present on the Ag-Cu surface than on the Cu foil surface. Therefore, the observed enhancement of CH_4_ formation on the Ag-Cu surface in CO_2_RR is apparently due to the tandem chemistry between Ag and Cu.

In CO_2_RR, CO adsorption can be difficult because CO adsorbate is suggested to come from the transformation of radical anion CO_2_^−•^ instead of direct CO adsorption^57,58^. In addition, the surface CO adsorption can also be negatively impacted by the near electrode cations that are attracted by electrostatic forces as the electrode potential is negatively biased during the electrolysis^50^. However, the near electrode cations can be effectively removed at a more positive potential (e.g., 0.4 V_RHE_), and CO adsorption can be promoted^50^. Based on these insights, square-wave potential electrolysis (inset of Fig. 6) of CO is employed to probe the catalytic behavior of our Ag-Cu surface with an increased local *CO concentration. The potential is alternated between 0.4 V_RHE_ (*U*_0_) for a fixed time interval of 0.01 s, at which the CO adsorption is maximized^50^, and an electrolysis potential (i.e., −1.1 V_RHE_, *U*_E_) at which the CO reduction is expected to occur. By flipping the electrode potential at a frequency of approximately 1 Hz, the FE toward hydrocarbon formation on the Ag-Cu surface is significantly improved from 5% to 18%, which surpassed that on a bare Cu surface. By increasing the frequency to approximately 2 Hz, the FE toward hydrocarbon formation is further increased to 30%, which is twice as high as that of bare Cu. These results suggest that the increase of electrode *CO concentration can efficiently improve the formation of further reduced products. The one-pot tandem catalysis mechanism that unitizes the complementary surface chemistry between a CO-producing material and Cu can be a very effective strategy for achieving this goal. However, to achieve more valuable products (e.g., C_2+_ products) requires further development employing more active CO-producing catalysts (e.g., nanoporous Ag^4^ or oxide-derived Au^5^) and CO reduction catalysts (e.g., oxide-derived Cu^59,60^) as well as their combination pattern and structure design for binding *CO to more desired sites after spillover.

## Discussion

Density functional theory (DFT) calculations were carried out to investigate the one-pot tandem catalysis of the CO_2_RR on Cu-modified Ag and Au surface models. We found that the surface Cu stabilized *CO by 1.07 and 1.09 eV compared to Ag and Au, respectively, indicating that the abundant *CO produced on the Ag or Au surface can migrate to surface Cu for further reduction. We found that all reactions along the possible pathways are downhill under a potential bias of −1.0 V_SHE_, indicating that the migrated *CO from the Ag surface can be further reduced on the surface Cu with barriers that are not larger than those on a bare Cu surface. Electrochemical studies were conducted using well-defined Ag-Cu surfaces with tunable Cu coverages to confirm our computational predictions. The CO spillover phenomenon was experimentally demonstrated for the first time. At optimum Cu coverage, nearly 70% of the CO produced on Ag can be further reduced on surface Cu, resulting in a high CH_4_ FE of approximately 60%. This FE is much higher than that on a bare Cu surface, which has intrinsically limited surface *CO. In addition, operando ATR-SEIRAS was employed to investigate the spillover of CO on the Ag-Cu surface. A dominating C≡O stretching band on the Ag-Cu surface with stronger binding strength was found at −0.4 V_RHE_, which was absent on the bare Ag and Cu surface, suggesting that the major *CO was not from CO_2_RR on Cu. The stronger adsorption of *CO on Cu as compared to Ag strongly suggest the CO spillover as a viable pathway. Moreover, we conducted square-wave potential electrolysis of CO to assess the role of a higher *CO concentration on hydrocarbon formation over the Ag-Cu surface. By alternating the electrolysis potential between a reductive point and a point that removes the near-surface cations, the FE for hydrocarbon formation was significantly enhanced owing to increased *CO adsorption. These results suggest that the increase of surface *CO could efficiently improve the formation of further reduced products. We conclude that the one-pot tandem catalysis mechanism unitizing the complementary surface chemistry between a CO-producing material and Cu can be a very effective strategy for achieving this goal. Further development employing more active CO-producing catalysts (e.g., nanoporous Ag^4^ or oxide-derived Au^5^) and CO reduction catalysts (e.g., oxide-derived Cu^59,60^) as well as their combination pattern and structure design provides a very promising route to achieve efficient CO_2_RRs toward more valuable products (e.g., C_2+_ products).

## Methods

### Computational details

The total energy of the Cu-modified Ag(111) and Au(111) surfaces with different adsorbates were calculated using DFT with the Perdew-Burke-Ernzerhof exchange-correlation functional^61^ in plane-wave pseudopotentials^62,63^, as implemented in the Vienna ab initio Simulation Package (VASP)^64,65^. The empirical D_2_ approach as implemented in VASP was employed to describe the van der Waals interactions^66^. All calculated energy values were extrapolated to *k*_B_*T* = 0. A Monkhorst–Pack *k*-point net of 3 × 6 × 1 was chosen to sample the reciprocal space for the slab calculations, and only the gamma point was sampled for the molecule calculations. A metal slab (4 × 2) consisting of 3 layers with the bottom layer fixed in its bulk position was employed to simulate the surface of Ag and Au, and a single layer of Cu with a coverage of 1/2 ML (monolayer) was placed on the substrate, as shown in Fig. 1a. A vacuum of 25 Å was introduced to each side to avoid interactions between successive metal slabs. Coordinates of all calculation models are provided (Supplementary Note 1).

The transition state for each reaction was first approached using the nudged elastic band (NEB) method in the neutral state^67^. Forces on the climbing image were converged to <0.02 eV Å^−1^. The plane-wave cutoff, smearing parameter and functional, and calculator parameters were the same as those used in slab geometry optimizations. Structures obtained from NEB were employed to generate the input structure and orientation for the dimer calculation^68^. The force of the dimer calculation was converged to <0.1 eV Å^−1^ to accurately locate the saddle point, i.e., the transition state. After that, the free energy of transition state was calculated under constant potential. An explicit water molecule was used as the proton source as previous work suggested^38^.

To establish the electrochemical interface, the approach proposed by Head-Gordon et al., Goddard et al., and Sautet et al. was applied^37–39^. In this model, the Fermi energy is adjusted to a target value by changing the number of electrons in the system during each step of the geometry optimization, which keeps the work function and electrode potential constant in the calculations. Then the linear Poisson–Boltzmann implicit solvation model with a Debye screening length of 3.0 Å was used to neutralize the non-zero charge in the simulation cell and simulate water and the electrolyte, allowing for a more realistic description of the electrochemical double layer. A detailed description of this approach has been provided in our previous work^36^.

### Electrolysis and product quantification

Ag foil (thickness 0.1 mm, 99.998% metal basis), Cu foil (thickness 0.1 mm, 99.9999% metal basis), Ti foil (thickness 0.127 mm, 99.99+% metal basis), and Ni wire (99.9%) were purchased from Alfa Aesar. A 5 mm × 18 mm piece of Ag or Cu foil was used as the working electrode in the CO_2_ and CO electrolysis experiments. The Ag foil was mechanically polished using sand paper (1200 G, 3 M) and thoroughly cleaned in an ultrasonic bath with deionized water prior to electrolysis. The Cu foil was mechanically polished using sand paper (1200 G, 3 M) followed by electrochemical polishing in phosphoric acid (85 wt.% in H_2_O, Sigma-Aldrich, 99.99% metal basis) at 2.0 V vs a Ti foil counter electrode and thorough rinsing in fresh 0.1 M NaHCO_3_ solution to remove phosphoric acid residue prior to each experiment. Ni wires were welded to the edge of these Ag and Cu foil pieces as current collectors.

The 0.1 M NaHCO_3_ solution was prepared by dissolving Na_2_CO_3_ (99.999%, Fluka) in deionized water that was obtained from a Millipore system (18.2 MΩ·cm) and converted to NaHCO_3_ using CO_2_ gas (99.99%, Air Liquide). The electrolyte was treated using Chelex® 100 resin (Sigma-Aldrich) prior to electrolysis. The 1 mM Cu^2+^ solution was prepared by dissolving Cu_2_SO_4_·5H_2_O (99.999%, Sigma-Aldrich) in 0.05 M sulfuric acid (99.999%, Sigma-Aldrich) according to a previously reported protocol^43^.

The CO_2_ electrolysis experiments were performed in a gas-tight two-compartment three-electrode electrochemical cell separated by a piece of a proton exchange membrane (Nafion^®^ perfluorinated membrane). A graphite rod (99.999%, Sigma-Aldrich) was used as the counter electrode. The cathodic compartment contained 18.0 mL of electrolyte and approximately 8.2 mL of headspace. Prior to electrolysis, the electrolyte in the cathodic compartment was purged with CO_2_ (99.99%, Air Liquide) gas for at least 25 min until a pH of 6.8 was reached. Then the Cu^2+^ solution was added for the Ag-Cu surface investigations. The electrolyte in the cathodic compartment was stirred at a rate of 800 rpm during the electrolysis.

The CO electrolysis experiments were performed under identical conditions as the CO_2_ electrolysis experiments except for the gas feed. Prior to electrolysis, the electrolyte in the cathodic compartment was purged with CO (99.999%, Air Liquide) gas rather than CO_2_ for at least 25 min, and the pH was measured to be 8.4.

The square-wave potential electrolysis was performed by alternating the potential between 0.4 V_RHE_ (*U*_0_) for a fixed time of 0.01 s and −1.1 V_RHE_ (*U*_E_) for 1 and 0.5 s, which is equivalent to a frequency of approximately 1 and 2 Hz, respectively. Only the cathodic charges were counted for the FE calculation. The charges from the capacitive current can be neglected owing to them being <1% of the total cathodic charges.

A Gamry Reference 600+ potentiostat was used for all electrolysis. All potentials were measured against a Ag/AgCl reference electrode (3.0 M KCl, BASi) and converted to the RHE reference scale using E (vs RHE) = E (vs Ag/AgCl) + 0.210 V + 0.05916 V × pH. The IR compensation function of the potentiostat was used to correct the electrode Ru.

The gas products were quantified using a gas chromatograph (Agilent 7890B). The gas chromatograph was equipped with a ShinCarbon ST Micropacked GC Column. Argon (99.999%, Air Liquide) was used as the carrier gas. First, the column effluent was passed through a thermal conductivity detector where the hydrogen was quantified. Then the effluent was passed through a methanizer where CO was converted to methane and subsequently quantified using a flame ionization detector.

The liquid products were quantified using a Bruker AVIII 400 MHz NMR spectrometer. After electrolysis, 0.5 mL of the electrolyte was mixed with 0.1 mL of D_2_O (99.9%, Sigma-Aldrich), and 1.67 ppm (*m*/*m*) dimethyl sulfoxide (≥99.9%, Alfa Aesar) was added as an internal standard. The ^1^H spectrum was measured with water suppression using a presaturation method.

### Operando ATR-SEIRAS

Al_2_O_3_, Na_2_S_2_O_3_ (98%), Na_2_SO_3_ (98%), NaOH (99.99%), KOH (99.99%), AgNO_3_ (99%), KCN (98%), HCHO (37 wt.%), HF (99%), NaAuCl_4_·2H_2_O and NH_4_Cl were purchased from Sigma-Aldrich.

The Au substrate film for the Ag film was deposited directly on the reflecting plane of Si prism using a chemical deposition method^50^. Before depositing, Si prism was first polished with a slurry of 0.05 μm Al_2_O_3_ and sonicated in acetone (Fisher Chemical) and deionized water. After sonicating, the Si prism was dried with air and immersed in NH_4_F (40%, Sigma-Aldrich) for 120 s to create a hydrogen-terminated surface. Then the reflecting surface was immersed into a mixture of 0.8 mL HF aqueous solution (2 wt.%) and 4.4 mL Au plating solution consisting of 5.75 mM NaAuCl_4_·2H_2_O, 0.025 M NH_4_Cl, 0.025 M Na_2_S_2_O_3_·5H_2_O, 0.075 M Na_2_SO_3_, and 0.026 M NaOH for 10 min. The temperature of the mixed solution was maintained at 55 °C during the deposition. After the deposition, the Au film was rinsed with deionized water and dried with air.

The Ag film was electrodeposited on the Au substrate film potentiostatically in a typical three-electrode system using the Au film as the working electrode, a graphite rod as the counter electrode, and saturated Ag/AgCl (BASi) as the reference electrode^49^. The electrolytic bath was prepared with deionized water and contained 0.15 M AgNO_3_, 0.54 M KCN, and 0.38 M Na_2_CO_3_. Electrodeposition in the prepared bath was carried out at 50 mV_RHE_ for 200 mC at room temperature. Afterwards, the obtained Ag film was rinsed with deionized water and dried with air. The Ag-Cu surface was then prepared in the same fashion as in reactivity study, i.e., by conducting the experiments in a Cu^2+^-containing bicarbonate on the Ag film.

The Cu reference film directly deposited on the Si prism was prepared using a similar method as described previously^51^. Briefly, the polished Si prism was immersed in NH_4_F for 60 s and then immersed into a copper seeding solution (0.5 wt.% HF and 750 μM CuSO_4_) for 120 s followed by a plating solution (0.25 M HCHO, 0.02 M CuSO_4_, 20 mM Na_2_EDTA (99–101%, ACS Reagent), and 0.3 mM 2,2-bipyridine (99%, Reagent Plus)) for 7 min. The pH of the plating solution was adjusted to 12.2 by KOH and the temperature was maintained at 55 °C during the deposition. Afterwards, the obtained Cu film was rinsed with deionized water and dried with air.

A two-compartment, three-electrode spectroelectrochemical cell, separated by a Nafion^®^ perfluorinated membrane, was designed to accommodate the Si prism and to avoid any possible cross-contamination from the counter electrode (Supplementary Fig. 9)^48^. NaHCO_3_ 0.1 M was used as the electrolyte. The ATR-SEIRAS experiments were conducted using an Agilent Technologies Cary 660 FTIR spectrometer equipped with a liquid nitrogen-cooled MCT detector. The spectrometer was coupled with a Solartron SI 1260/1287 system for electrochemical measurements. All spectra were collected at a 4 cm^−1^ spectral resolution and were presented in absorbance units. In a typical process, the obtained films on Si prisms were used as working electrodes with a graphite rod as the counter electrode and saturated Ag/AgCl as the reference electrode.

### Physical characterization

SEM images were recorded using a HITACHI S-5500 SEM. The accelerating voltage was 5 kV. TEM sample was prepared using Ga^+^ focused ion on a ZEISS AURIGA^®^ Field Emission-SEM implemented with CrossBeam^®^ Workstations. TEM images were recorded using a JEOL JEM-2010F TEM. The accelerating voltage was 200 kV. XPS measurements were carried out using a PHI Quantera II with Al Kα radiation. The resulting spectra were analyzed using the CasaXPS software package (Casa Software Ltd., U.K.) and peaks were fit using a Gaussian/Lorentzian product line shape with the Shirley-type background.

## Supplementary information

Supplementary information

## Data Availability

The data that support the findings of this study are available from the corresponding author upon request.
