# Supplementary material for: Computational and experimental demonstrations of one-pot tandem catalysis for electrochemical carbon dioxide reduction to methane
Source: Nat Commun. 2019 Jul 26;10:3340. doi: 10.1038/s41467-019-11292-9 (PMC6659690; doi:10.1038/s41467-019-11292-9)
Supplement: Supplementary file 1 — Supplementary information [file 41467_2019_11292_MOESM1_ESM.pdf]

Supplementary Information for

Zhang *et al*., Computational and experimental demonstrations of one-pot tandem catalysis for electrochemical carbon dioxide reduction to methane

Supplementary Table 1. ΔGs (eV) for CO among all surface sites on Ag-Cu and Au-Cu surfaces under -1.0 V_{SHE}.

Ag-Cu			Au-Cu		
fcc	hcp	atop	fcc	hcp	atop
0.72	0.70	0.84	0.44	0.29	0.72
0.78	0.17	0.40	0.28	-0.41	-0.13
-0.34	-0.32	0.40	-0.75	-0.71	-0.20
0.27	0.47	0.44	-0.10	-0.10	-0.15

Supplementary Table 2. Free energy barriers (eV) for CO spillover from pure Ag or Au sites to surface Cu sites at -1.0 V_{SHE} on Ag-Cu and Au-Cu surfaces.

Spillover	1 to 2	2 to 3	3 to 4	4 to 5
Ag-Cu	0.09	0.09	0.01	0.07
Au-Cu	0.16	0.13	0.14	0.13

Supplementary Table 3. Δ*G*s (eV) for all intermediates under zero charge and -1.0 V_{SHE} on Ag-Cu and Au-Cu surfaces.

	Ag-Cu		Au-Cu	
	zero-charge	-1.0 V_{SHE}	zero-charge	-1.0 V_{SHE}
*CO	-0.12	-0.34	-0.25	-0.75
*CHO	0.76	0.56	0.58	0.25
*COH	0.85	1.06	0.53	0.72
*CHOH	0.75	0.88	0.45	0.84
*CH_2O	0.63	1.12	0.25	0.95
*C	0.99	0.92	0.75	0.49
*OCH_3	-0.41	-0.11	-0.66	-0.40
*CH_2OH	0.47	0.51	-0.02	0.36
*CH	0.60	0.44	0.45	0.07
*CH_2	0.16	0.16	-0.35	-0.47
*$HOCH_3$	-0.16	0.52	-0.62	0.18
*O	-1.10	-1.30	-1.06	-1.70
*OH	-1.57	-1.22	-1.81	-1.49
*CH_3	-0.41	-0.33	-0.94	-0.49

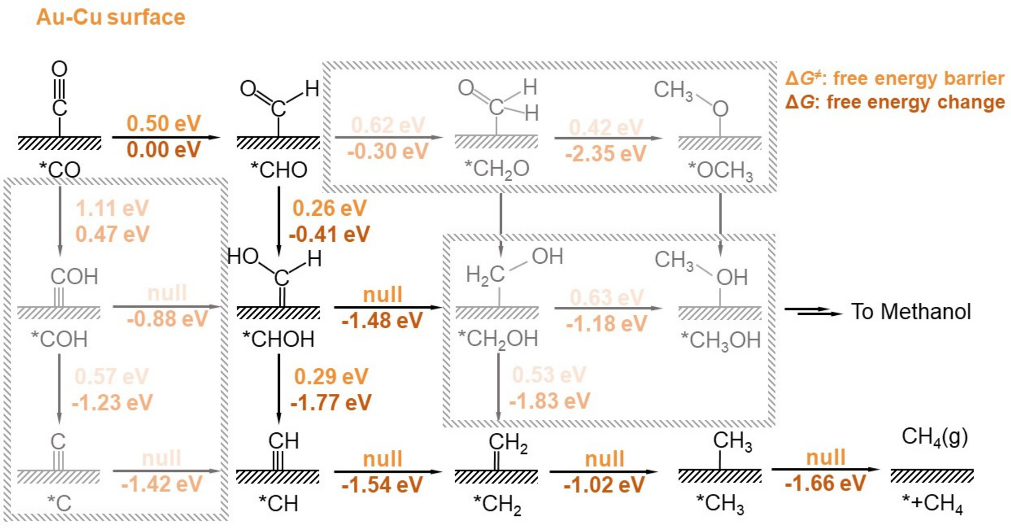

Supplementary Figure 1: Kinetics and free energy diagram for CO reduction to C_1 products on the Au-Cu surface. The values shown orange (upper) and dark orange (bottom) are the free energy barrier and free energy change at -1.0 V_{SHE} for all steps respectively. Adsorbates with an asterisk correspond species that are adsorbed on the surface. Null stands for no free energy barrier.

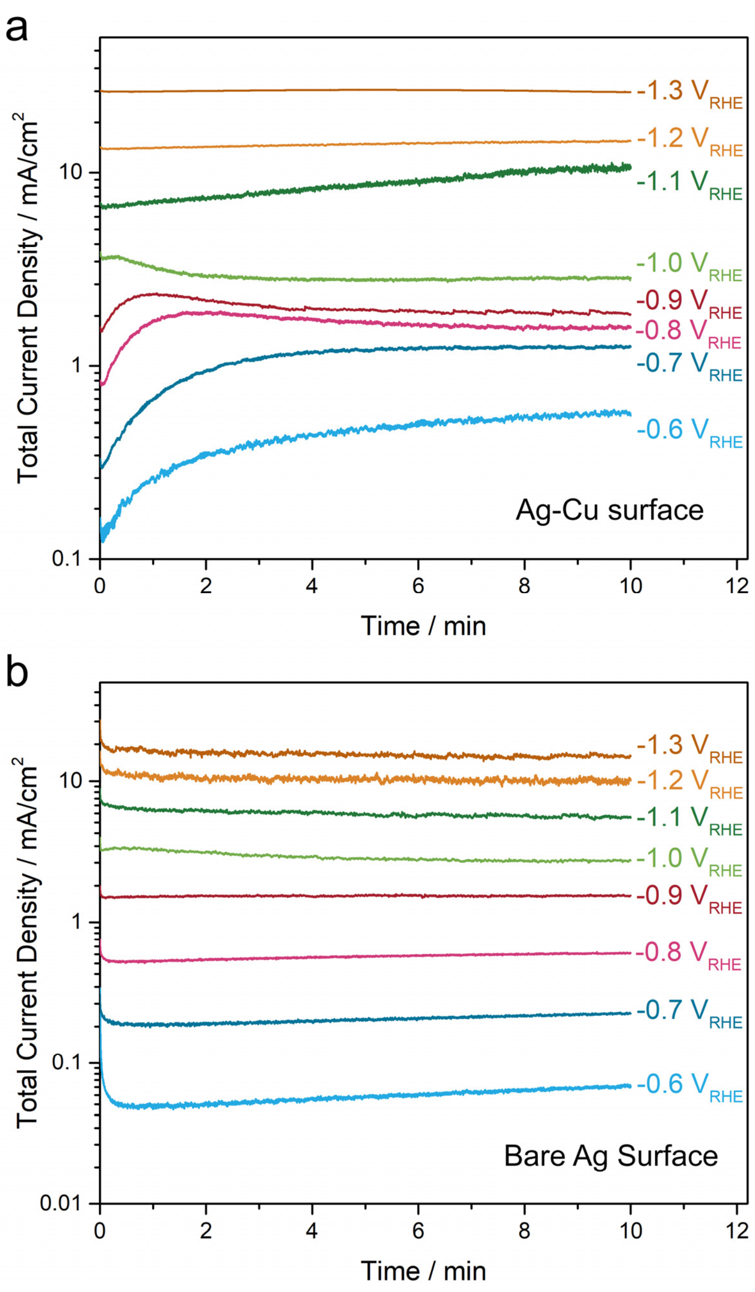

Supplementary Figure 2: CO_2 electrolysis current densities for the Ag-Cu surface achieved at a Cu^{2+} concentration of 1.5 ppm (**a**) and bare Ag (**b**) in a potential range of -0.6 V_{RHE} to -1.3 V_{RHE}.

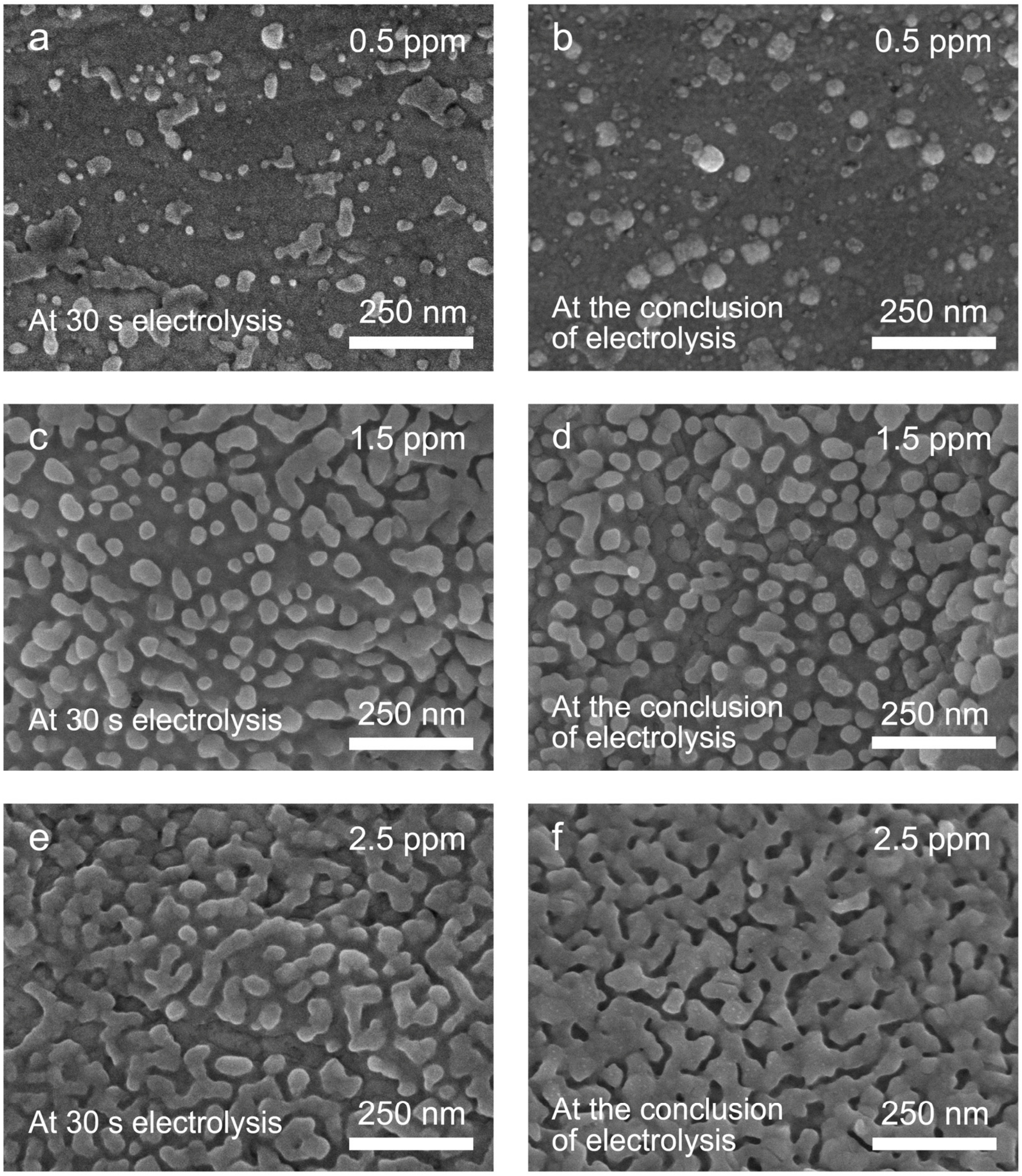

Supplementary Figure 3: SEM images of Ag-Cu surfaces achieved at a Cu^{2+} concentration of 0.5 (**a**, **b**), 1.5 (**c**, **d**) and 2.5ppm (**e**, **f**) at 30 s electrolysis (**a**, **c**, **e**) and at the conclusion of electrolysis (**b**, **d**, **f**) at -1.1 V_{RHE}.

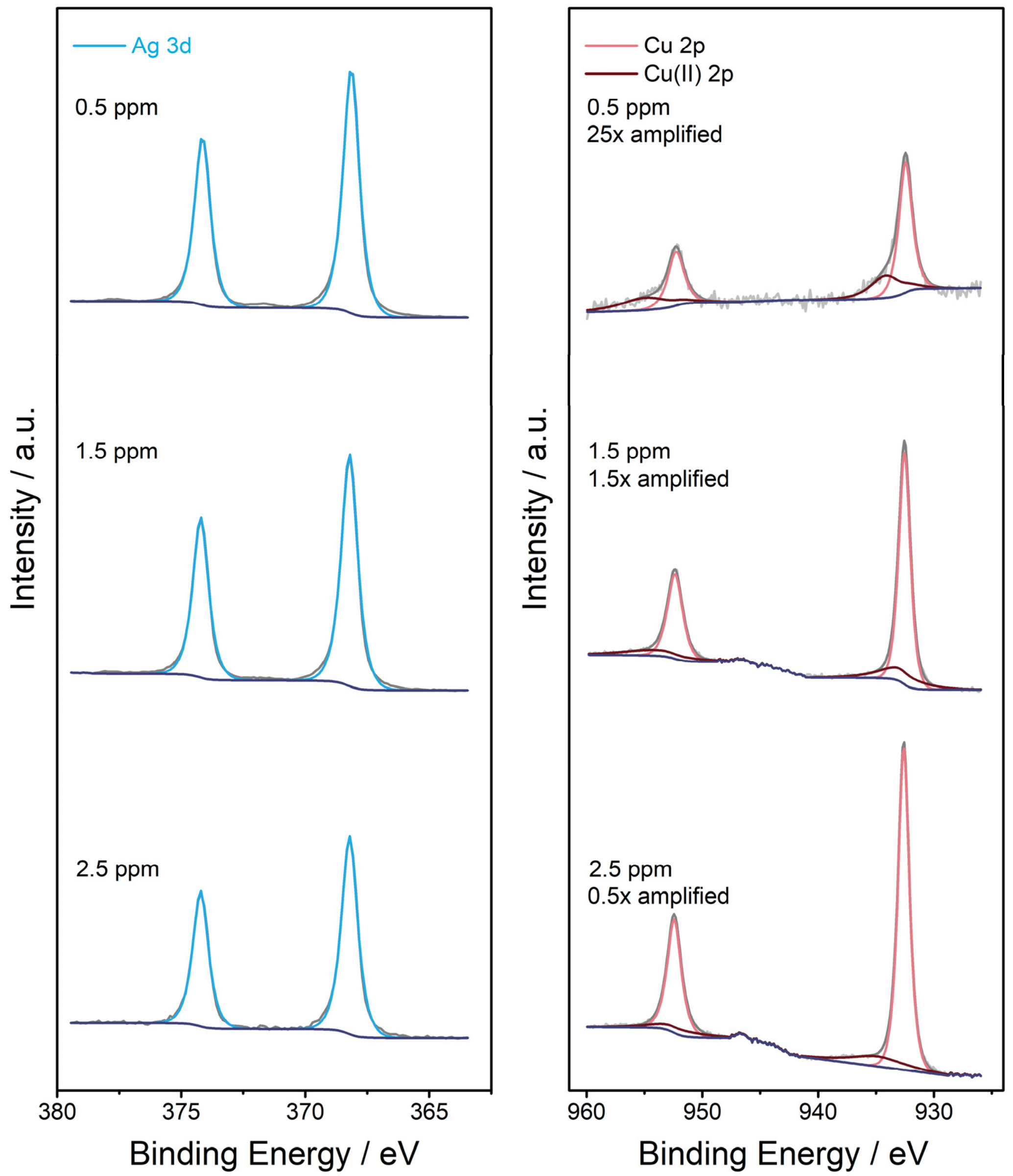

Supplementary Figure 4: Representative XPS spectra of Ag-Cu surfaces achieved at a Cu^{2+} concentration of 0.5, 1.5 and 2.5 ppm. (**a**), Ag 3d spectra. (**b**), Cu 2p spectra. The small amount of oxides observed in the Cu spectra were most likely due to material handling in atmospheric air. The peak assignment and fitting parameters were referenced from Supplementary Ref 1-4.

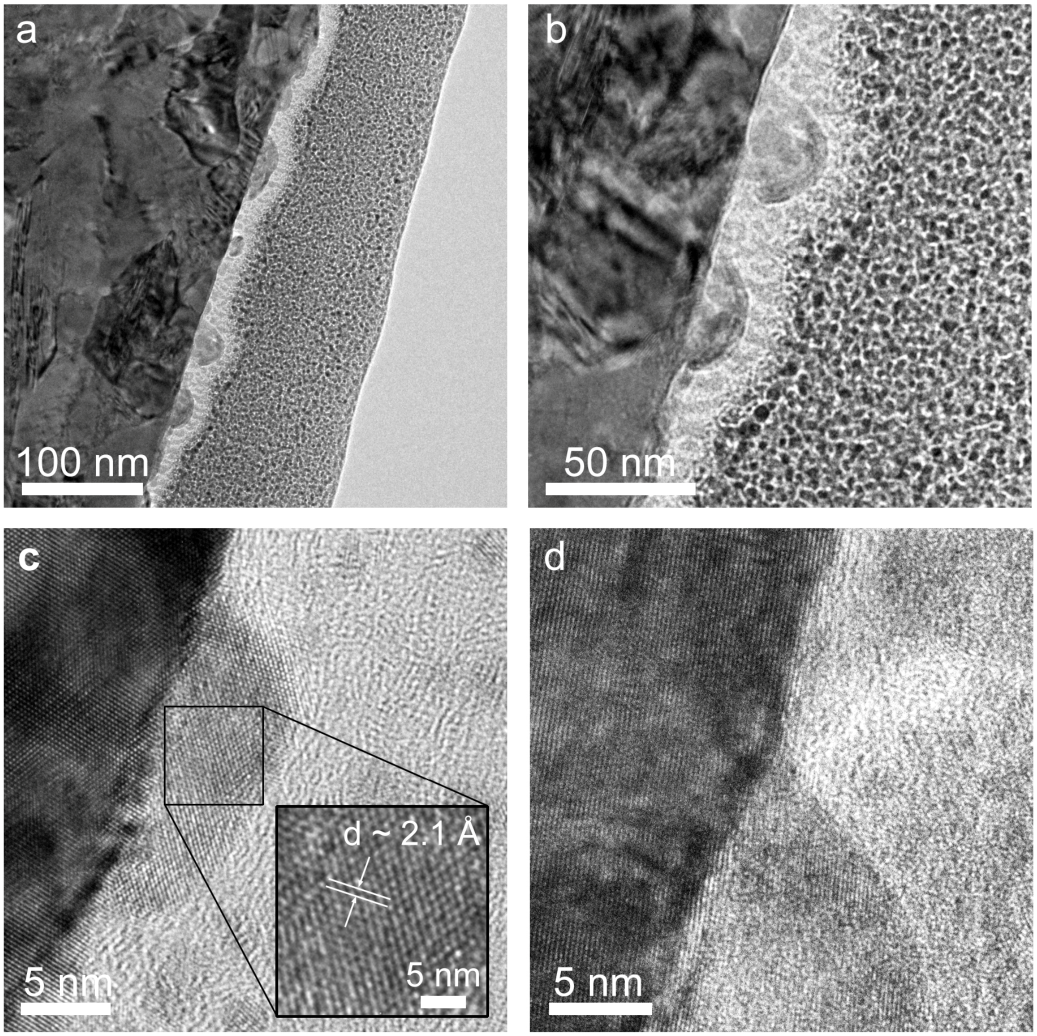

Supplementary Figure 5: TEM images of Ag-Cu surfaces with magnifications of 100k (**a**) and 400k (**b**). **c, d,** High-resolution TEM images of Ag-Cu surfaces. The inset of **c** exhibits lattice fringes from surface Cu with interplanar spacings corresponding to Cu(111). The Ag-Cu surface sample was prepared at a Cu^{2+} concentration of 1.5 ppm with an electrolysis potential of -1.1 V_{RHE}. A protective Pt film was applied to avoid undesirable damages of surface Cu when employing the FIB technique.

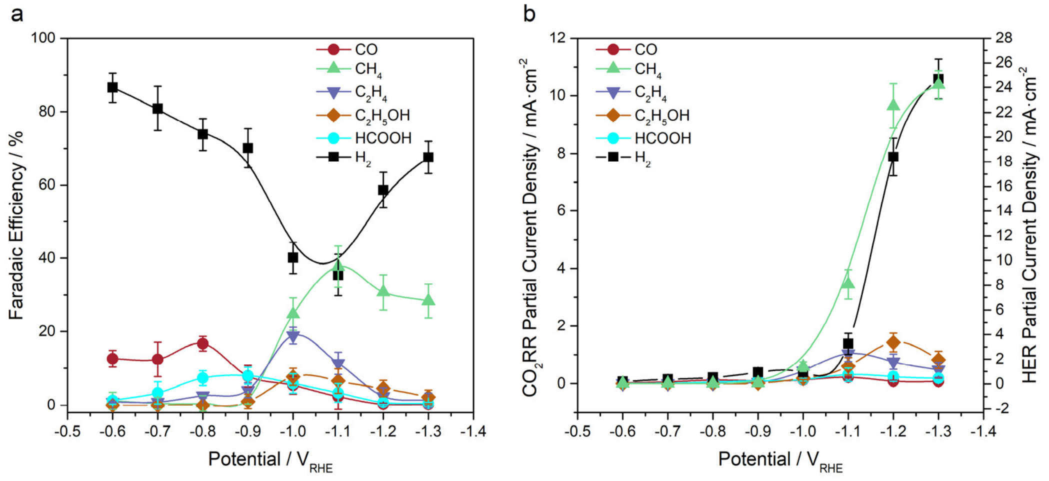

Supplementary Figure 6: Faradaic efficiencies (**a**) and partial current densities (**b**) of CO_2 electrolysis products on a bare polycrystalline Cu surface. The error bars represent the standard deviation from at least three independent measurements.

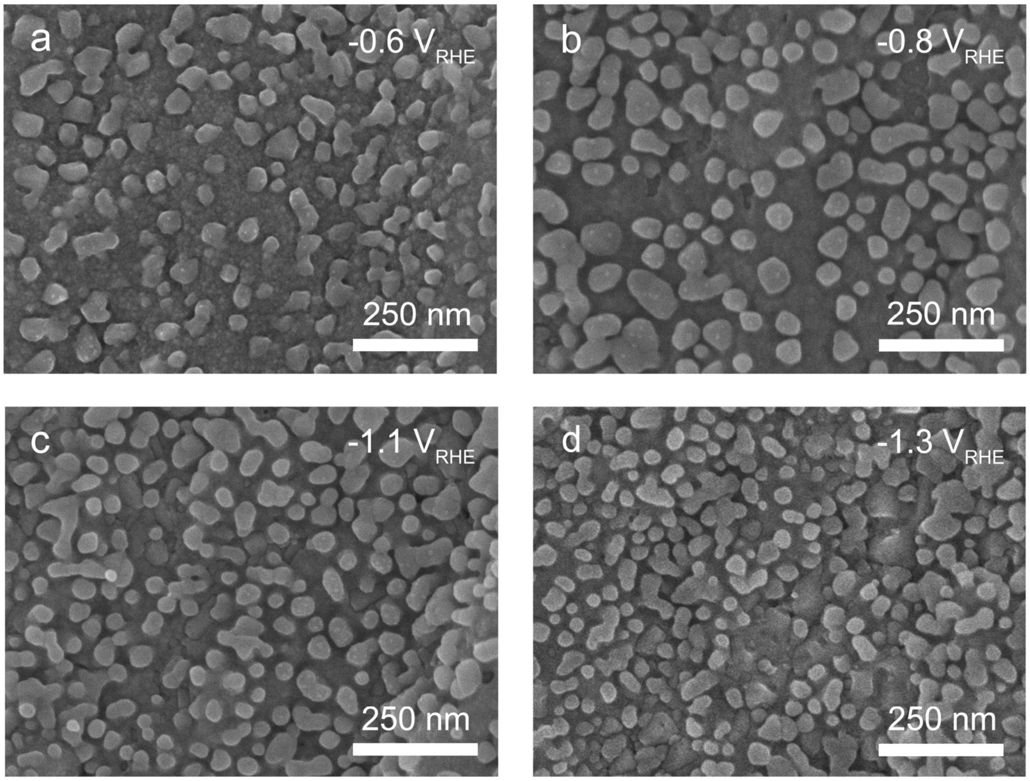

Supplementary Figure 7: SEM images of Ag-Cu surfaces achieved at a Cu^{2+} concentration of 1.5 ppm with electrolysis potentials of (**a**) -0.6 V_{RHE}, (**b**) -0.8 V_{RHE}, (**c**) -1.1 V_{RHE} and (**d**) -1.3 V_{RHE}.

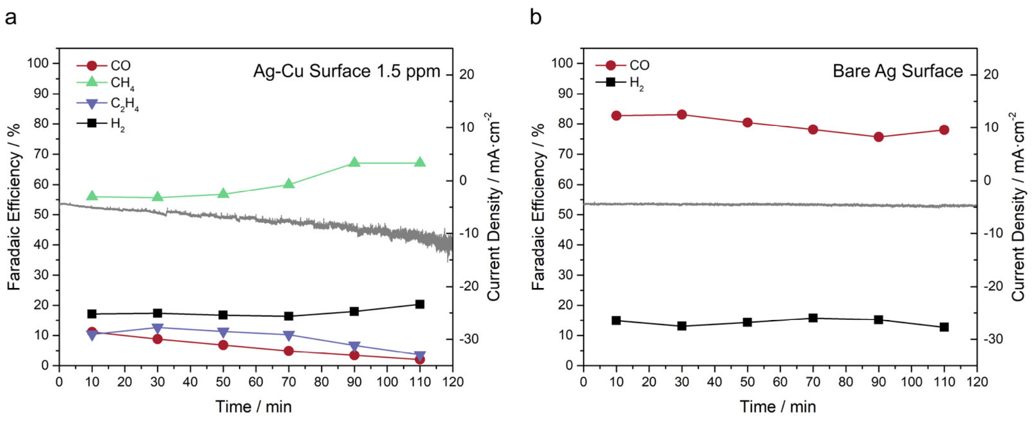

Supplementary Figure 8: 2-hour electrolysis for (**a**) the Ag-Cu surface with 1.5 ppm Cu^{2+} and (**b**) the bare Ag surface at -1.1 V_{RHE}. The further increase of CH_4 FE is likely due to the surface reconstruction of Cu under CO_2 electroreduction conditions so that CH_4 formation becomes more preferred[5, 6].

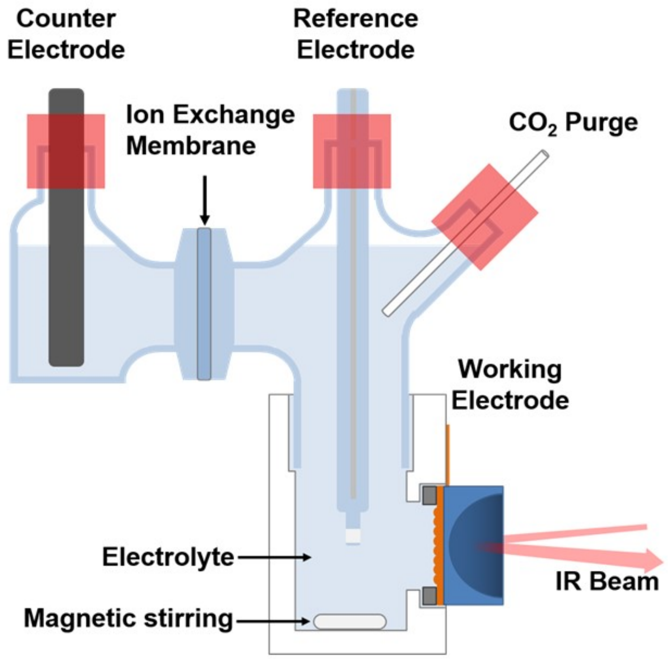

Supplementary Figure 9: The custom-designed stirred spectroelectrochemical cell for ATR-SEIRAS study.

Supplementary Note 1

Coordinates for intermediate calculations.

Ag-Cu surface

*CO

```
   11.5560998917000006    0.0000000000000000    0.0000000000000000
   -2.8890500068999998    5.0039813974999996    0.0000000000000000
    0.0000000000000000    0.0000000000000000   32.0765991211000028
   Ag   Cu   C    O
    24     4     1     1
Selective dynamics
Direct
  0.0000000000000000  0.0000000000000000  0.1558800110000007   F   F   F
  0.0000000049999969  0.5000000189999980  0.1558800110000007   F   F   F
  0.2500000000000000  0.0000000000000000  0.1558800110000007   F   F   F
  0.2500000049999969  0.5000000189999980  0.1558800110000007   F   F   F
  0.5000000000000000  0.0000000000000000  0.1558800110000007   F   F   F
  0.4999999839999987  0.5000000189999980  0.1558800110000007   F   F   F
  0.7500000209999982  0.0000000000000000  0.1558800110000007   F   F   F
  0.7500000459999967  0.5000000189999980  0.1558800110000007   F   F   F
  0.0794177525941361  0.3301643131508613  0.2297059182193502   T   T   T
  0.0783091927213102  0.8297590711364358  0.2288682527000688   T   T   T
  0.3365097335472256  0.3420158337223375  0.2288332730707673   T   T   T
  0.3371797300612374  0.8405788926091988  0.2288290501780124   T   T   T
  0.6023958994681936  0.3501472522433517  0.2308263352686327   T   T   T
  0.5990759634489415  0.8475037964409864  0.2330965234584185   T   T   T
  0.8408984805829272  0.3403079482166855  0.2463456324980573   T   T   T
  0.8407771628937256  0.8405819854268823  0.2460135066818097   T   T   T
  0.1617936630947270  0.1615430536020960  0.3013201868702668   T   T   T
  0.1603108877290969  0.6689255372432017  0.3004276934706766   T   T   T
  0.4334482746620893  0.1909461430663472  0.2982735862121939   T   T   T
  0.4254075641053969  0.6855448340122735  0.3007034977086275   T   T   T
  0.6700992650849662  0.1707374447494745  0.3221846854288921   T   T   T
  0.6716411704172203  0.6725821601944796  0.3215859135084952   T   T   T
  0.9287070894078024  0.1795179699668728  0.3244688218935475   T   T   T
  0.9281220752639797  0.6793828690830302  0.3247056134394218   T   T   T
  0.2372240448181187  0.9866586550145255  0.3655910553120926   T   T   T
  0.2371776875411795  0.5001188305480503  0.3637906523122953   T   T   T
  0.4414578182790970  0.9433471098387921  0.3697196390553474   T   T   T
  0.4399791584707331  0.4553949313143763  0.3688688843890335   T   T   T
  0.3847616389558644  0.1486728609976543  0.4082036013647584   T   T   T
  0.3908657369254483  0.1567324533301890  0.4454224064101279   T   T   T
```

*CHO

```
   11.5560998917000006    0.0000000000000000    0.0000000000000000
   -2.8890500068999998    5.0039813974999996    0.0000000000000000
    0.0000000000000000    0.0000000000000000   32.0765991211000028
   Ag   Cu   C    O    H
    24     4     1     1     1
Selective dynamics
Direct
```

```
 0.0000000000000000  0.0000000000000000  0.1558800110000007   F   F   F
 0.0000000049999969  0.5000000189999980  0.1558800110000007   F   F   F
 0.2500000000000000  0.0000000000000000  0.1558800110000007   F   F   F
 0.2500000049999969  0.5000000189999980  0.1558800110000007   F   F   F
 0.5000000000000000  0.0000000000000000  0.1558800110000007   F   F   F
 0.4999999839999987  0.5000000189999980  0.1558800110000007   F   F   F
 0.7500000209999982  0.0000000000000000  0.1558800110000007   F   F   F
 0.7500000459999967  0.5000000189999980  0.1558800110000007   F   F   F
 0.0809891926140359  0.3274611506044466  0.2284957236780819   T   T   T
 0.0810358159492128  0.8347674689851948  0.2283999157827452   T   T   T
 0.3369098224547252  0.3360056701696453  0.2288862839696292   T   T   T
 0.3384123877107226  0.8368442564223362  0.2283813163577209   T   T   T
 0.6004103138069773  0.3508910319404712  0.2314137230736082   T   T   T
 0.5997650291968781  0.8513867450621463  0.2316643965683818   T   T   T
 0.8434000838792571  0.3435639493829257  0.2439583372907163   T   T   T
 0.8412021782334299  0.8414855319647500  0.2443322168194549   T   T   T
 0.1628726020379557  0.1621651347189967  0.3026432532495171   T   T   T
 0.1541750783223117  0.6554802245856961  0.2984359429485062   T   T   T
 0.4287018943441308  0.1780072697446010  0.2993601807781392   T   T   T
 0.4283268045215733  0.6755897250659297  0.2998576961343254   T   T   T
 0.6758716897771974  0.1779391203010749  0.3186055668653826   T   T   T
 0.6708272561287272  0.6726954515558918  0.3201761915823282   T   T   T
 0.9270059275432940  0.1756979704905866  0.3228759982809120   T   T   T
 0.9265121203861835  0.6793906511855570  0.3227435544801723   T   T   T
 0.2446524784421439  0.9684056800600543  0.3631953652896766   T   T   T
 0.2434915691740616  0.5216633894692604  0.3622903694836967   T   T   T
 0.4558406213652971  0.9532691362296171  0.3671474513456822   T   T   T
 0.4355999503429878  0.4306789953886909  0.3691191736705868   T   T   T
 0.4259640263170745  0.4225162811799972  0.4288968442392901   T   T   T
 0.4720722808519564  0.3182850977647188  0.4517345215737932   T   T   T
 0.3831148219545221  0.5392017201877187  0.4432914386197340   T   T   T
```

*COH

```
   11.5560998917000006    0.0000000000000000    0.0000000000000000
   -2.8890500068999998    5.0039813974999996    0.0000000000000000
    0.0000000000000000    0.0000000000000000   32.0765991211000028
  Ag   Cu   C    O    H
  24    4    1    1    1
Selective dynamics
Direct
 0.0000000000000000  0.0000000000000000  0.1558800110000007   F   F   F
 0.0000000049999969  0.5000000189999980  0.1558800110000007   F   F   F
 0.2500000000000000  0.0000000000000000  0.1558800110000007   F   F   F
 0.2500000049999969  0.5000000189999980  0.1558800110000007   F   F   F
 0.5000000000000000  0.0000000000000000  0.1558800110000007   F   F   F
 0.4999999839999987  0.5000000189999980  0.1558800110000007   F   F   F
 0.7500000209999982  0.0000000000000000  0.1558800110000007   F   F   F
 0.7500000459999967  0.5000000189999980  0.1558800110000007   F   F   F
 0.0811814873596092  0.3305781349536319  0.2302841913447082   T   T   T
 0.0809159800280773  0.8312159144051375  0.2311011387435653   T   T   T
 0.3370152894756657  0.3326154624705496  0.2296659906595901   T   T   T
 0.3371915975989485  0.8375118972928127  0.2296560753862359   T   T   T
 0.5981486292656693  0.3491538906322364  0.2335483515692244   T   T   T
```

```
0.6018876888442412  0.8532926300941539  0.2312496395969985   T   T   T
0.8411595179200123  0.3415237890311681  0.2457630695877124   T   T   T
0.8412146300545965  0.8405870811029672  0.2452325143104237   T   T   T
0.1651417537924415  0.1702179278929450  0.3037929488956482   T   T   T
0.1646264201968247  0.6624014720659644  0.3030587940278514   T   T   T
0.4299932003906452  0.1768749715416043  0.3029532672728482   T   T   T
0.4333346357397590  0.6796828640563727  0.2988720938500549   T   T   T
0.6722338934495146  0.1736700599548613  0.3225799078675458   T   T   T
0.6752237103128874  0.6775968660278809  0.3214407399039673   T   T   T
0.9298343652495885  0.1810175583328089  0.3250475160673509   T   T   T
0.9315351280291120  0.6819479792005000  0.3251960749394286   T   T   T
0.2460924347636003  0.9965402380620474  0.3660331532744173   T   T   T
0.2265545431751026  0.4810404619472044  0.3701624626923514   T   T   T
0.4412102011932104  0.9317371360889510  0.3709450488579766   T   T   T
0.4494240751545881  0.4441885032492090  0.3712374933152486   T   T   T
0.3819696169283722  0.6167124274839917  0.4064454087866293   T   T   T
0.3820117986877511  0.6329293261663391  0.4478246623294037   T   T   T
0.4436605854340170  0.5853396426206501  0.4609765888391443   T   T   T
```

*CHOH

```
   11.5560998917000006    0.0000000000000000    0.0000000000000000
   -2.8890500068999998    5.0039813974999996    0.0000000000000000
    0.0000000000000000    0.0000000000000000   32.0765991211000028
   Ag   Cu   C    O    H
   24    4    1    1    2
Selective dynamics
Direct
  0.0000000000000000  0.0000000000000000  0.1558800110000007   F   F   F
  0.0000000049999969  0.5000000189999980  0.1558800110000007   F   F   F
  0.2500000000000000  0.0000000000000000  0.1558800110000007   F   F   F
  0.2500000049999969  0.5000000189999980  0.1558800110000007   F   F   F
  0.5000000000000000  0.0000000000000000  0.1558800110000007   F   F   F
  0.4999999839999987  0.5000000189999980  0.1558800110000007   F   F   F
  0.7500000209999982  0.0000000000000000  0.1558800110000007   F   F   F
  0.7500000459999967  0.5000000189999980  0.1558800110000007   F   F   F
  0.0784033916357784  0.3342574808498709  0.2290007868997262   T   T   T
  0.0807161612200912  0.8287942879771670  0.2296745713832929   T   T   T
  0.3378292459967310  0.3280372081839315  0.2284792050883501   T   T   T
  0.3341380133934152  0.8180724628284503  0.2280603860866581   T   T   T
  0.6021843382168223  0.3686943118965293  0.2319794775271576   T   T   T
  0.5999052962611516  0.8687196444080648  0.2323283120382099   T   T   T
  0.8402763003497594  0.3465111707727073  0.2473480709602924   T   T   T
  0.8439220625315823  0.8500272233438929  0.2428874523438212   T   T   T
  0.1575333553563683  0.1681314506891404  0.2994075592207865   T   T   T
  0.1639626399257112  0.6632438507756411  0.3037436202773370   T   T   T
  0.4229314930445366  0.1443277404319738  0.2967755721745837   T   T   T
  0.4320689641325188  0.6597992536215639  0.3010766324847091   T   T   T
  0.6645128449160083  0.1809105987817604  0.3245723887992776   T   T   T
  0.6828438018649506  0.6977920730039671  0.3173797994326418   T   T   T
  0.9292833596725081  0.1901452301571481  0.3249261990153112   T   T   T
  0.9300980083546764  0.6867335599603912  0.3245152602494462   T   T   T
  0.2378696795675231  0.0216727395499894  0.3633927006213976   T   T   T
  0.2568784173912298  0.4618340501636702  0.3671125684096294   T   T   T
```

0.4250100862133481 0.9326898727881119 0.3702777823649600 T T T
0.4671234003043488 0.4056921032368924 0.3645244549275110 T T T
0.3203886008960075 0.6916342089916503 0.4167900568220340 T T T
0.3894121287488248 0.6614474025330996 0.4488178233184962 T T T
0.2419686812122599 0.7200975420686968 0.4315345853803524 T T T
0.4595997602699936 0.6281267891757275 0.4381096468572201 T T T

*CH_2O

11.5560998917000006 0.0000000000000000 0.0000000000000000
-2.8890500068999998 5.0039813974999996 0.0000000000000000
0.0000000000000000 0.0000000000000000 32.0765991211000028
Ag Cu C O H
24 4 1 1 2
Selective dynamics
Direct
0.0000000000000000 0.0000000000000000 0.1558800106620666 F F F
0.0000000048476565 0.5000000192346832 0.1558800106620666 F F F
0.2500000000064873 0.0000000000000000 0.1558800106620666 F F F
0.2500000047676068 0.5000000192346832 0.1558800106620666 F F F
0.5000000000129816 0.0000000000000000 0.1558800106620666 F F F
0.4999999841789133 0.5000000192346832 0.1558800106620666 F F F
0.7500000206146566 0.0000000000000000 0.1558800106620666 F F F
0.7500000460574938 0.5000000192346832 0.1558800106620666 F F F
0.0841221695286371 0.3413672975430477 0.2308329059084167 T T T
0.0845048267217963 0.8378412497448763 0.2310937381365459 T T T
0.3388570793241706 0.3417784926681730 0.2302483204451687 T T T
0.3381261160108641 0.8479368361102985 0.2288242887857755 T T T
0.5947437560708225 0.3337671551158702 0.2360531097591503 T T T
0.6022745876939097 0.8451657305080997 0.2314072738857328 T T T
0.8408433090446290 0.3425686114541722 0.2464502975038987 T T T
0.8459868386862494 0.8446421810470963 0.2397664100265736 T T T
0.1668535363579759 0.1785578524947826 0.3039898634644027 T T T
0.1704781474222730 0.6811117984610292 0.3035207790815601 T T T
0.4268922233795204 0.2095473023991529 0.3075719466917214 T T T
0.4292871922358262 0.6987312421522880 0.2984864836189007 T T T
0.6711345676708212 0.1642109640020762 0.3248161297284267 T T T
0.6882004363114917 0.6813484942546305 0.3132158488967822 T T T
0.9310105189308872 0.1866472021523352 0.3242899873405994 T T T
0.9340599669226176 0.6863341018131265 0.3248796066536646 T T T
0.2271658843788402 -0.0374007841949220 0.3712749749032269 T T T
0.2128814786485051 0.5000669892322713 0.3718271836404723 T T T
0.4355375043954698 0.9261851409810641 0.3689782444914804 T T T
0.5029977814568867 0.5799885010507438 0.3701354786622502 T T T
0.3896096766703251 0.4253235964998436 0.4207971748310059 T T T
0.2587529649796006 0.2691726717248726 0.4064632566765578 T T T
0.4306970243548297 0.3045779285407925 0.4340490428331401 T T T
0.4007894949210578 0.5841784568021305 0.4421748297970088 T T T

*C

11.5560998917000006 0.0000000000000000 0.0000000000000000
-2.8890500068999998 5.0039813974999996 0.0000000000000000
0.0000000000000000 0.0000000000000000 32.0765991211000028

```
  Ag   Cu   C
  24    4    1
Selective dynamics
Direct
  0.0000000000000000  0.0000000000000000  0.1558800110000007   F   F   F
  0.0000000049999969  0.5000000189999980  0.1558800110000007   F   F   F
  0.2500000000000000  0.0000000000000000  0.1558800110000007   F   F   F
  0.2500000049999969  0.5000000189999980  0.1558800110000007   F   F   F
  0.5000000000000000  0.0000000000000000  0.1558800110000007   F   F   F
  0.4999999839999987  0.5000000189999980  0.1558800110000007   F   F   F
  0.7500000209999982  0.0000000000000000  0.1558800110000007   F   F   F
  0.7500000459999967  0.5000000189999980  0.1558800110000007   F   F   F
  0.0772459574301152  0.3466213641576175  0.2274217427658247   T   T   T
  0.0777211478712824  0.8369521043828734  0.2279579360151506   T   T   T
  0.3403742588515059  0.3117267233572339  0.2286178892998178   T   T   T
  0.3378582747080699  0.8086243779201174  0.2291945648664594   T   T   T
  0.6027642269390232  0.3823008766547729  0.2343073064510184   T   T   T
  0.6024630854709374  0.8842254102719648  0.2345706964208687   T   T   T
  0.8407137098795500  0.3659593788881340  0.2484768287099577   T   T   T
  0.8455606463205211  0.8670172582217289  0.2433200368003234   T   T   T
  0.1557679511345609  0.1799807650525105  0.2956214430056042   T   T   T
  0.1620134420463623  0.6932683446422696  0.3012366078722806   T   T   T
  0.4269500089530147  0.1266583276489112  0.3001439439490117   T   T   T
  0.4297320309997882  0.6294314046497647  0.3007780771139482   T   T   T
  0.6656102486563711  0.1718901188774390  0.3282100282693608   T   T   T
  0.6912182711787447  0.6875123484417422  0.3172358977016486   T   T   T
  0.9255953001870980  0.2049328328402475  0.3244506529699664   T   T   T
  0.9414728044090092  0.7167567022668069  0.3317129721311773   T   T   T
  0.2489340714510263  0.0775614300099411  0.3641059227581577   T   T   T
  0.1680068488874488  0.4291671127330329  0.3653703080732704   T   T   T
  0.3928981171269610  0.8357795146157517  0.3698816241982298   T   T   T
  0.4856408473443462  0.4141417855509800  0.3678314042953705   T   T   T
  0.3362383517855520  0.4601363031851298  0.3766858995753647   T   T   T
```

*CH

```
   11.5560998917000006    0.0000000000000000    0.0000000000000000
   -2.8890500068999998    5.0039813974999996    0.0000000000000000
    0.0000000000000000    0.0000000000000000   32.0765991211000028
  Ag   Cu   C    H
  24    4    1    1
Selective dynamics
Direct
  0.0000000000000000  0.0000000000000000  0.1558800110000007   F   F   F
  0.0000000049999969  0.5000000189999980  0.1558800110000007   F   F   F
  0.2500000000000000  0.0000000000000000  0.1558800110000007   F   F   F
  0.2500000049999969  0.5000000189999980  0.1558800110000007   F   F   F
  0.5000000000000000  0.0000000000000000  0.1558800110000007   F   F   F
  0.4999999839999987  0.5000000189999980  0.1558800110000007   F   F   F
  0.7500000209999982  0.0000000000000000  0.1558800110000007   F   F   F
  0.7500000459999967  0.5000000189999980  0.1558800110000007   F   F   F
  0.0807897379596919  0.3309058051201599  0.2296981850020564   T   T   T
  0.0801588193425466  0.8301799623637919  0.2299852952059802   T   T   T
  0.3370794329444631  0.3337537313856987  0.2297225794896107   T   T   T
```

```
0.3380869230226899  0.8376814927871737  0.2297250939589549   T   T   T
0.5987142037576616  0.3496541524786171  0.2327478801804608   T   T   T
0.6004983638226157  0.8519717229179495  0.2320882193409895   T   T   T
0.8411181092597566  0.3422840125561173  0.2455143196707014   T   T   T
0.8411113295951171  0.8411658280092149  0.2449576688058260   T   T   T
0.1635418490095552  0.1651353922297704  0.3023609838930458   T   T   T
0.1624728003743764  0.6601769312674002  0.3019380703097439   T   T   T
0.4309464398196029  0.1744958542754966  0.3021587119251945   T   T   T
0.4324648754784371  0.6767120672952978  0.2999133643069583   T   T   T
0.6738639876307009  0.1755877231606909  0.3222791974247950   T   T   T
0.6758448089653770  0.6739221477843914  0.3209812506303047   T   T   T
0.9292290210100470  0.1788214698678023  0.3248520606661665   T   T   T
0.9330581117458276  0.6830455147358772  0.3263975477931867   T   T   T
0.2401751346382608  0.9888499389005155  0.3655655653918151   T   T   T
0.2126932030074538  0.4656387084225925  0.3701401295853458   T   T   T
0.4382809301679800  0.9332684840628606  0.3715135032231499   T   T   T
0.4443676445651966  0.4307527890079166  0.3712014171509542   T   T   T
0.3723523209647948  0.6088001370411470  0.4020508581412967   T   T   T
0.3732636732252414  0.6080873746320069  0.4363502179155343   T   T   T
```

*CH_2OH

```
  11.5560998917000006    0.0000000000000000    0.0000000000000000
  -2.8890500068999998    5.0039813974999996    0.0000000000000000
   0.0000000000000000    0.0000000000000000   32.0765991211000028
 Ag   Cu   C    O    H
 24    4   1    1    3
Selective dynamics
Direct
0.0000000000000000  0.0000000000000000  0.1558800110000007   F   F   F
0.0000000049999969  0.5000000189999980  0.1558800110000007   F   F   F
0.2500000000000000  0.0000000000000000  0.1558800110000007   F   F   F
0.2500000049999969  0.5000000189999980  0.1558800110000007   F   F   F
0.5000000000000000  0.0000000000000000  0.1558800110000007   F   F   F
0.4999999839999987  0.5000000189999980  0.1558800110000007   F   F   F
0.7500000209999982  0.0000000000000000  0.1558800110000007   F   F   F
0.7500000459999967  0.5000000189999980  0.1558800110000007   F   F   F
0.0804826040836635  0.3339053958494006  0.2290346096841850   T   T   T
0.0809205981600486  0.8270945931577203  0.2290524752263358   T   T   T
0.3381411030440813  0.3396091089405548  0.2287544412614903   T   T   T
0.3365829415622921  0.8344343031187279  0.2291185970274638   T   T   T
0.6012639558330680  0.3520883831525164  0.2315001273631250   T   T   T
0.5986775015832424  0.8484820016181288  0.2325875748541365   T   T   T
0.8413295322340636  0.3406440798780326  0.2452043505874945   T   T   T
0.8431964315723062  0.8428955472388066  0.2446025615229432   T   T   T
0.1541318520331756  0.1545581474606651  0.2989890488269596   T   T   T
0.1627426685552417  0.6634926884042236  0.3032784608907136   T   T   T
0.4277482463183477  0.1752945726328691  0.2987129643793459   T   T   T
0.4290039020685265  0.6814112182231571  0.3018848084451365   T   T   T
0.6715714118056818  0.1752595349392246  0.3205407259546061   T   T   T
0.6748804656519665  0.6757780671032401  0.3195896406197504   T   T   T
0.9274070474301948  0.1797534141721870  0.3245037570199273   T   T   T
0.9279790077209124  0.6769030861404129  0.3249174893338947   T   T   T
0.2374158944806860  0.0150207783073567  0.3639372118750832   T   T   T
```

0.2465397848106210 0.4698493473873933 0.3630981588843359 T T T
0.4322859467064012 0.9532147404894661 0.3704758527731622 T T T
0.4518172697970034 0.4316790437894902 0.3677104672844874 T T T
0.4383794663642175 0.9449850715902076 0.4320605366425103 T T T
0.3399312183813115 0.7002430100066988 0.4498752802404276 T T T
0.4271324945820051 0.1050939567144548 0.4461810698759472 T T T
0.5408250213881259 0.9805811950388039 0.4366993590202730 T T T
0.3532219075431243 0.5565855114861952 0.4390064139020906 T T T

*OCH_3

```
   11.5560998917000006    0.0000000000000000    0.0000000000000000
   -2.8890500068999998    5.0039813974999996    0.0000000000000000
    0.0000000000000000    0.0000000000000000   32.0765991211000028
   Ag   Cu   C    O    H
   24    4   1    1    3
Selective dynamics
Direct
  0.0000000000000000  0.0000000000000000  0.1558800110000007   F   F   F
  0.0000000049999969  0.5000000189999980  0.1558800110000007   F   F   F
  0.2500000000000000  0.0000000000000000  0.1558800110000007   F   F   F
  0.2500000049999969  0.5000000189999980  0.1558800110000007   F   F   F
  0.5000000000000000  0.0000000000000000  0.1558800110000007   F   F   F
  0.4999999839999987  0.5000000189999980  0.1558800110000007   F   F   F
  0.7500000209999982  0.0000000000000000  0.1558800110000007   F   F   F
  0.7500000459999967  0.5000000189999980  0.1558800110000007   F   F   F
  0.0773938656502076  0.3257635850311680  0.2279668260929013   T   T   T
  0.0790049613922997  0.8339024526576360  0.2288775712924667   T   T   T
  0.3366759175858114  0.3393068912651530  0.2296661774409907   T   T   T
  0.3381858420763458  0.8405450352256219  0.2296998103156016   T   T   T
  0.5979634400292220  0.3482169024612014  0.2322828157523793   T   T   T
  0.5982336752772259  0.8468792940715308  0.2331984230335395   T   T   T
  0.8415015151356749  0.3426285483997472  0.2438129514178642   T   T   T
  0.8395611534514662  0.8399923052878350  0.2462238049153154   T   T   T
  0.1603184449276113  0.1691357594278539  0.3020823750069304   T   T   T
  0.1518224269508879  0.6506671940827992  0.2982310929249705   T   T   T
  0.4269372598341834  0.1855179663323203  0.3012765873912813   T   T   T
  0.4283468381318707  0.6841306977866146  0.3022285520496134   T   T   T
  0.6750589637049130  0.1756819852742384  0.3191100673583574   T   T   T
  0.6705266819138441  0.6688515998095306  0.3224823518626456   T   T   T
  0.9259021383089479  0.1762211442027836  0.3257106520127509   T   T   T
  0.9272476038266989  0.6801428491794824  0.3253328155387952   T   T   T
  0.2290040839240126  0.9619355815170135  0.3624456971634687   T   T   T
  0.2377007968825154  0.5213836547067946  0.3635739597571064   T   T   T
  0.4506553709067006  0.9928680502229366  0.3707166974121476   T   T   T
  0.4302233981343196  0.4029031621288286  0.3728690427019331   T   T   T
  0.3805466791127321  0.6707867505599855  0.4494805174016564   T   T   T
  0.4012561666836029  0.6648234734120204  0.4049579530876414   T   T   T
  0.2862214693218472  0.4940773800221070  0.4588634549657639   T   T   T
  0.3758301990366862  0.8516988363039593  0.4569802163936478   T   T   T
  0.4641360603687673  0.6747518202915248  0.4664765432771352   T   T   T
```

*CH_2

```
 11.5560998917000006    0.0000000000000000    0.0000000000000000
 -2.8890500068999998    5.0039813974999996    0.0000000000000000
  0.0000000000000000    0.0000000000000000   32.0765991211000028
 Ag   Cu   C    H
  24    4    1    2
Selective dynamics
Direct
 0.0000000000000000  0.0000000000000000  0.1558800110000007   F   F   F
 0.0000000049999969  0.5000000189999980  0.1558800110000007   F   F   F
 0.2500000000000000  0.0000000000000000  0.1558800110000007   F   F   F
 0.2500000049999969  0.5000000189999980  0.1558800110000007   F   F   F
 0.5000000000000000  0.0000000000000000  0.1558800110000007   F   F   F
 0.4999999839999987  0.5000000189999980  0.1558800110000007   F   F   F
 0.7500000209999982  0.0000000000000000  0.1558800110000007   F   F   F
 0.7500000459999967  0.5000000189999980  0.1558800110000007   F   F   F
 0.0806119108335950  0.3318171967769079  0.2299259412598749   T   T   T
 0.0808107944751665  0.8269804556631627  0.2296410284924759   T   T   T
 0.3376138708507886  0.3439183223002128  0.2289862197205526   T   T   T
 0.3367252383701716  0.8387076100185844  0.2298632711808990   T   T   T
 0.6025787279686375  0.3517167597730337  0.2304910636253015   T   T   T
 0.5984931380552768  0.8470094467131697  0.2338325823959796   T   T   T
 0.8411895445734955  0.3389083171063235  0.2452830227440237   T   T   T
 0.8425105566191907  0.8405532266290610  0.2447747479662805   T   T   T
 0.1598550450525827  0.1574889655383995  0.3003937620458575   T   T   T
 0.1659413807979490  0.6613696164914769  0.3045116547765514   T   T   T
 0.4339053604188370  0.1925970081038525  0.2980476146269843   T   T   T
 0.4294175417654529  0.6869109805550430  0.3031402488089732   T   T   T
 0.6715647981276551  0.1784758007812362  0.3216343250481818   T   T   T
 0.6745002439769110  0.6778550944929249  0.3210701838448448   T   T   T
 0.9287975942942629  0.1770603807620827  0.3245881323472349   T   T   T
 0.9308055188868624  0.6768652381266996  0.3252852912117428   T   T   T
 0.2251052448680085  0.0013201918171139  0.3675716453219208   T   T   T
 0.2447800153761489  0.4503006944449404  0.3679300160531145   T   T   T
 0.4260451607419455  0.9701938895744359  0.3698330529176722   T   T   T
 0.4479735565373347  0.4213459008932896  0.3687152860257519   T   T   T
 0.3310482694337449  0.7073083931195534  0.4120872311022323   T   T   T
 0.2463619045872764  0.6853478061892219  0.4310686447570157   T   T   T
 0.4121939573818030  0.7267024169483149  0.4325898478621462   T   T   T
```

*$HOCH_3$

```
 11.5560998917000006    0.0000000000000000    0.0000000000000000
 -2.8890500068999998    5.0039813974999996    0.0000000000000000
  0.0000000000000000    0.0000000000000000   32.0765991211000028
 Ag   Cu   C    O    H
  24    4    1    1    4
Selective dynamics
Direct
 0.0000000000000000  0.0000000000000000  0.1558800106620666   F   F   F
 0.0000000048476565  0.5000000192346832  0.1558800106620666   F   F   F
 0.2500000000064873  0.0000000000000000  0.1558800106620666   F   F   F
 0.2500000047676068  0.5000000192346832  0.1558800106620666   F   F   F
 0.5000000000129816  0.0000000000000000  0.1558800106620666   F   F   F
 0.4999999841789133  0.5000000192346832  0.1558800106620666   F   F   F
```

0.7500000206146566	0.0000000000000000	0.1558800106620666	F	F	F
0.7500000460574938	0.5000000192346832	0.1558800106620666	F	F	F
0.0764210058391151	0.3374283009374179	0.2278767966689988	T	T	T
0.0790260702070316	0.8312778509667937	0.2294159426357804	T	T	T
0.3371135974776411	0.3406091654982172	0.2290769750852555	T	T	T
0.3340260198362722	0.8336392693419321	0.2288507353221815	T	T	T
0.5980059762793496	0.3481746253705941	0.2319316585926507	T	T	T
0.5959395357838195	0.8473604108575139	0.2331528350138270	T	T	T
0.8372718663528699	0.3391450902055664	0.2460378348504560	T	T	T
0.8404435594335722	0.8424728687801836	0.2432566233749457	T	T	T
0.1492697968447452	0.1717928532795971	0.2980283459673959	T	T	T
0.1588759258677754	0.6704768447954039	0.3034627944174886	T	T	T
0.4231491581408135	0.1736435362344041	0.2988404175706635	T	T	T
0.4244228067347910	0.6824011752168665	0.3023257895305734	T	T	T
0.6632216086899436	0.1750640563187769	0.3235668489799597	T	T	T
0.6738127844639139	0.6840447822440633	0.3182286898119829	T	T	T
0.9216530385136785	0.1865805204437429	0.3235855286556724	T	T	T
0.9240436460200505	0.6850779067849732	0.3232802630009023	T	T	T
0.2351653333226477	0.0372923827410813	0.3610830820572166	T	T	T
0.2425110098785792	0.4772828066890176	0.3641421732842224	T	T	T
0.4284451645113013	0.9629439662162750	0.3700626649762174	T	T	T
0.4546146554602741	0.4389035953314595	0.3669683753587111	T	T	T
0.3626300695153191	0.6112991987740818	0.4576717993373317	T	T	T
0.3255609847948718	0.7422989792658760	0.4260154361971259	T	T	T
0.2802046254148045	0.4057694955947679	0.4620438068865689	T	T	T
0.3857550312719414	0.7236473960638761	0.4870472005968375	T	T	T
0.4514002532592513	0.6095554709529698	0.4460002214312224	T	T	T
0.2640253933888927	0.7937794247129152	0.4380853097427383	T	T	T

*CH_3

```
   11.5560998917000006    0.0000000000000000    0.0000000000000000
   -2.8890500068999998    5.0039813974999996    0.0000000000000000
    0.0000000000000000    0.0000000000000000   32.0765991211000028
  Ag   Cu   C    H
   24    4    1    3
Selective dynamics
Direct
```

0.0000000000000000	0.0000000000000000	0.1558800110000007	F	F	F
0.0000000049999969	0.5000000189999980	0.1558800110000007	F	F	F
0.2500000000000000	0.0000000000000000	0.1558800110000007	F	F	F
0.2500000049999969	0.5000000189999980	0.1558800110000007	F	F	F
0.5000000000000000	0.0000000000000000	0.1558800110000007	F	F	F
0.4999999839999987	0.5000000189999980	0.1558800110000007	F	F	F
0.7500000209999982	0.0000000000000000	0.1558800110000007	F	F	F
0.7500000459999967	0.5000000189999980	0.1558800110000007	F	F	F
0.0791571050947186	0.3293128556269018	0.2291542684510416	T	T	T
0.0794879030440295	0.8299354163098978	0.2291932483517601	T	T	T
0.3367607979874303	0.3384822327738276	0.2282298912625173	T	T	T
0.3368875465736378	0.8366448297644897	0.2281985380108446	T	T	T
0.5997568887891562	0.3500720633414076	0.2317589864518375	T	T	T
0.5995520132331155	0.8494975980409711	0.2320855920481993	T	T	T
0.8409019165429565	0.3412296872188532	0.2451307215494060	T	T	T
0.8406963272389890	0.8401750524424039	0.2453226718424062	T	T	T

0.1617548551416534	0.1658916383048866	0.3011828928522656	T	T	T
0.1613930711938348	0.6585340294624196	0.3009809735278026	T	T	T
0.4245429868825274	0.1754920899789074	0.2983519783639739	T	T	T
0.4265460154988892	0.6775533948378103	0.3003242359408287	T	T	T
0.6721740371311626	0.1743532482120949	0.3203493571913371	T	T	T
0.6712643714940060	0.6734655507299627	0.3207578143845398	T	T	T
0.9274124795058947	0.1782273014480734	0.3235008579738236	T	T	T
0.9301888053350847	0.6812941141066206	0.3242421823193529	T	T	T
0.2369690447626060	0.9858694956436456	0.3638914995042363	T	T	T
0.2489174469136898	0.5009777357768869	0.3670988761815461	T	T	T
0.4436773719369455	0.9629073744216464	0.3670210982300037	T	T	T
0.4425271655918744	0.4239589122982810	0.3671287941544216	T	T	T
0.2412883511776591	0.4905373385880738	0.4278006108299812	T	T	T
0.1364547246323867	0.4351574038095325	0.4337448085404361	T	T	T
0.3089110560472633	0.6834140300612616	0.4425788563428710	T	T	T
0.2608073597729283	0.3358072954455584	0.4401231131319666	T	T	T

Au-Cu surface

*CO

11.5352001190000006	0.0000000000000000	0.0000000000000000
-2.8838000298000002	4.9948881704000003	0.0000000000000000
0.0000000000000000	0.0000000000000000	32.0638008118000002

Au	Cu	C	O
24	4	1	1

Selective dynamics

Direct

0.0000000000000000	0.0000000000000000	0.1559400000000011	F	F	F
0.0000000029999967	0.5000000140000012	0.1559400000000011	F	F	F
0.2500000000000000	0.0000000000000000	0.1559400000000011	F	F	F
0.2500000029999967	0.5000000140000012	0.1559400000000011	F	F	F
0.5000000000000000	0.0000000000000000	0.1559400000000011	F	F	F
0.4999999830000021	0.5000000140000012	0.1559400000000011	F	F	F
0.7499999590000002	0.0000000000000000	0.1559400000000011	F	F	F
0.7499999409999987	0.5000000140000012	0.1559400000000011	F	F	F
0.0617077943314782	0.3115495354344764	0.2319069803142846	T	T	T
0.0606882190725681	0.8109310542477421	0.2313097162856049	T	T	T
0.3250554386156298	0.3253335876998952	0.2403238968871350	T	T	T
0.3245673243056201	0.8237255979449007	0.2403083659092267	T	T	T
0.5993015996634132	0.3495046633175738	0.2271987873841078	T	T	T
0.5956689944564431	0.8460590218898049	0.2288198838127242	T	T	T
0.8242673395316852	0.3233594737588552	0.2542649605968211	T	T	T
0.8248647753081993	0.8256044047710004	0.2541259669384847	T	T	T
0.1370025479621514	0.1347331260521592	0.3048821379747849	T	T	T
0.1363227963116003	0.6394816005550484	0.3047390473556979	T	T	T
0.4768991644545753	0.2292849350389102	0.3010233973823581	T	T	T
0.4748643682283534	0.7289027196469621	0.3030079641633106	T	T	T
0.6993028404279125	0.2006272973672022	0.3354036936381529	T	T	T
0.7003068955262588	0.6986463478739777	0.3347972876569594	T	T	T
0.9364622567040836	0.1863417837913275	0.3458418010587256	T	T	T
0.9356830109493908	0.6852920655675769	0.3466123279394791	T	T	T
0.2669103920590039	0.0189982430680457	0.3588011170653650	T	T	T
0.2687835737696738	0.5220359244670326	0.3557582677818401	T	T	T
0.4640897953416444	0.9648178866851339	0.3721343190781006	T	T	T

0.4664957068538264 0.4762277786007975 0.3713157151449281 T T T
0.3908176108311502 0.1506871596044416 0.4081275634104475 T T T
0.3845942399257817 0.1533245086166272 0.4452207864744391 T T T

*CHO

11.5352001190000006 0.0000000000000000 0.0000000000000000
-2.8838000298000002 4.9948881704000003 0.0000000000000000
0.0000000000000000 0.0000000000000000 32.0638008118000002
Au Cu C O H
24 4 1 1 1
Selective dynamics
Direct
0.0000000000000000 0.0000000000000000 0.1559400000000011 F F F
0.0000000029999967 0.5000000140000012 0.1559400000000011 F F F
0.2500000000000000 0.0000000000000000 0.1559400000000011 F F F
0.2500000029999967 0.5000000140000012 0.1559400000000011 F F F
0.5000000000000000 0.0000000000000000 0.1559400000000011 F F F
0.4999999830000021 0.5000000140000012 0.1559400000000011 F F F
0.7499999590000002 0.0000000000000000 0.1559400000000011 F F F
0.7499999409999987 0.5000000140000012 0.1559400000000011 F F F
0.0624467514923019 0.3125195634450777 0.2306025566784452 T T T
0.0626255398975382 0.8172941370759919 0.2303092980391341 T T T
0.3237843573417748 0.3231203510417499 0.2371524119524850 T T T
0.3275995534612072 0.8298886163961285 0.2373304894844581 T T T
0.5956481040330044 0.3462749635537751 0.2286819607692243 T T T
0.5980285904154208 0.8490548467823835 0.2280162167363308 T T T
0.8264127614792466 0.3277103585607183 0.2512643247846489 T T T
0.8255578215312593 0.8258265817075899 0.2526406686495241 T T T
0.1404411607573620 0.1454619987488636 0.3042581445374896 T T T
0.1328049418594112 0.6394661462038439 0.3014585965997721 T T T
0.4712406231729949 0.2266086129732317 0.3030988536361703 T T T
0.4666900434691296 0.7199580935307544 0.3007376347762726 T T T
0.6989300481067153 0.2004940886067734 0.3314448216900202 T T T
0.6954925393950160 0.7005324314696775 0.3325832184246015 T T T
0.9335078151884653 0.1857029813369180 0.3425556595356040 T T T
0.9323467732608146 0.6879348469343711 0.3422243176561976 T T T
0.2721904122920579 0.0125585531958639 0.3547217230466537 T T T
0.2645225491444325 0.5425820380561531 0.3554372158147699 T T T
0.4713296867134326 0.9647010691115177 0.3710805665518840 T T T
0.4598258784332899 0.4954350257890470 0.3714787499084248 T T T
0.4530857693964616 0.4290782123525577 0.4302249063973330 T T T
0.4847908911836584 0.2687316245833118 0.4428415085813057 T T T
0.4296806302815607 0.5461552553803726 0.4529083799904753 T T T

*COH

11.5352001190000006 0.0000000000000000 0.0000000000000000
-2.8838000298000002 4.9948881704000003 0.0000000000000000
0.0000000000000000 0.0000000000000000 32.0638008118000002
Au Cu C O H
24 4 1 1 1
Selective dynamics
Direct

0.0000000000000000 0.0000000000000000 0.1559400000000011 F F F
0.0000000029999967 0.5000000140000012 0.1559400000000011 F F F
0.2500000000000000 0.0000000000000000 0.1559400000000011 F F F
0.2500000029999967 0.5000000140000012 0.1559400000000011 F F F
0.5000000000000000 0.0000000000000000 0.1559400000000011 F F F
0.4999999830000021 0.5000000140000012 0.1559400000000011 F F F
0.7499999590000002 0.0000000000000000 0.1559400000000011 F F F
0.7499999409999987 0.5000000140000012 0.1559400000000011 F F F
0.0643542154768035 0.3138128590829901 0.2304507097142021 T T T
0.0656077427390352 0.8210627252439522 0.2303194898390084 T T T
0.3259182435082052 0.3181559075790288 0.2361984904465359 T T T
0.3299126087438666 0.8253396242729446 0.2347253569040247 T T T
0.6009494927108473 0.3527191060806876 0.2292543210180828 T T T
0.6070843206438762 0.8588617391593395 0.2271225833680660 T T T
0.8302386757689413 0.3330818536492041 0.2549996104746304 T T T
0.8296716808429929 0.8295476186933345 0.2555672931859684 T T T
0.1495890256325928 0.1611591765290314 0.3056703840234850 T T T
0.1426050468615527 0.6484889625892541 0.3010205120212147 T T T
0.4636673021868282 0.2100982953622546 0.3022363706857891 T T T
0.4672730743664575 0.7149157286310390 0.2976027534974753 T T T
0.6874608156379106 0.1897303463098171 0.3331099085580359 T T T
0.6870279837918871 0.6934030100130913 0.3333118661484001 T T T
0.9280532836475032 0.1836823637231922 0.3365244218070276 T T T
0.9269587080840833 0.6828892849918395 0.3348982382665192 T T T
0.2617506856880811 -0.0009013876715645 0.3583238047485653 T T T
0.2350856494866726 0.5194530524751225 0.3644464936381294 T T T
0.4553867782531059 0.9379094417959205 0.3693030483908684 T T T
0.4506198569139262 0.4562446872539955 0.3681171794622135 T T T
0.3798308293424726 0.6148694841848866 0.4039999797891058 T T T
0.3769378600568494 0.6289209522028230 0.4450384390501481 T T T
0.4137990524933146 0.5287516473767656 0.4594103146937301 T T T

*CHOH

11.5352001190000006 0.0000000000000000 0.0000000000000000
-2.8838000298000002 4.9948881704000003 0.0000000000000000
0.0000000000000000 0.0000000000000000 32.0638008118000002
Au Cu C O H
24 4 1 1 2
Selective dynamics
Direct
0.0000000000000000 0.0000000000000000 0.1559400000000011 F F F
0.0000000029999967 0.5000000140000012 0.1559400000000011 F F F
0.2500000000000000 0.0000000000000000 0.1559400000000011 F F F
0.2500000029999967 0.5000000140000012 0.1559400000000011 F F F
0.5000000000000000 0.0000000000000000 0.1559400000000011 F F F
0.4999999830000021 0.5000000140000012 0.1559400000000011 F F F
0.7499999590000002 0.0000000000000000 0.1559400000000011 F F F
0.7499999409999987 0.5000000140000012 0.1559400000000011 F F F
0.0644263732093001 0.3221291167801451 0.2291795745315398 T T T
0.0658106907662064 0.8169228415930245 0.2298242110624806 T T T
0.3331919579459467 0.3264416211014539 0.2342197022942399 T T T
0.3275136630550785 0.8176120989029372 0.2348828395650848 T T T
0.6062178880844398 0.3598115216429633 0.2281212598721500 T T T

```
0.6042039380615374  0.8588137293847541  0.2286315583555945   T   T   T
0.8299366445584543  0.3335597425884575  0.2563518316108010   T   T   T
0.8320281705498912  0.8353590991371901  0.2539799051830431   T   T   T
0.1400210713557195  0.1549757407057889  0.2996957299170077   T   T   T
0.1501291537912063  0.6545477165757880  0.3041313466968133   T   T   T
0.4628010974449061  0.2053737070708779  0.2985764236961825   T   T   T
0.4656564580511660  0.7076048428852312  0.3010677415317650   T   T   T
0.6829451254958507  0.1887293958236866  0.3343891762654537   T   T   T
0.6931790771452212  0.6973821764307379  0.3319288146443309   T   T   T
0.9269392755521232  0.1883998519888829  0.3352201855665933   T   T   T
0.9299806370374978  0.6836739955432598  0.3355530109076613   T   T   T
0.2570838533233089  0.0455687899209292  0.3555217282935761   T   T   T
0.2671578046106555  0.4895654032368334  0.3602789650597707   T   T   T
0.4418759422334824  0.9633357331938960  0.3691767724112644   T   T   T
0.4683724305457986  0.4379669987579702  0.3690059632690926   T   T   T
0.3347328799355814  0.7032556079583285  0.4122731197128703   T   T   T
0.2356688403637326  0.6955917304093781  0.4365326946122826   T   T   T
0.4041971747223522  0.6841135216596916  0.4339105885590498   T   T   T
0.1723879679618567  0.7170177999994578  0.4185464747088474   T   T   T
```

*CH_2O

```
   11.5352001190000006    0.0000000000000000    0.0000000000000000
   -2.8838000298000002    4.9948881704000003    0.0000000000000000
    0.0000000000000000    0.0000000000000000   32.0638008118000002
   Au   Cu   C    O    H
   24    4   1    1    2
Selective dynamics
Direct
  0.0000000000000000  0.0000000000000000  0.1558800106620666   F   F   F
  0.0000000048476565  0.5000000192346832  0.1558800106620666   F   F   F
  0.2500000000064873  0.0000000000000000  0.1558800106620666   F   F   F
  0.2500000047676068  0.5000000192346832  0.1558800106620666   F   F   F
  0.5000000000129816  0.0000000000000000  0.1558800106620666   F   F   F
  0.4999999841789133  0.5000000192346832  0.1558800106620666   F   F   F
  0.7500000206146566  0.0000000000000000  0.1558800106620666   F   F   F
  0.7500000460574938  0.5000000192346832  0.1558800106620666   F   F   F
  0.0777119974305638  0.3357979068687736  0.2302570670761620   T   T   T
  0.0788556925182116  0.8285588091661704  0.2304183256616935   T   T   T
  0.3351550457043413  0.3394782656936983  0.2319366885746157   T   T   T
  0.3375880191189058  0.8470759616583028  0.2311791803925975   T   T   T
  0.5946478323058473  0.3468888138689781  0.2326424022865029   T   T   T
  0.5996079714604008  0.8564209289749800  0.2299428452379654   T   T   T
  0.8351588123791864  0.3388769156956833  0.2520963917557220   T   T   T
  0.8375755167829508  0.8373797182880901  0.2350610712587645   T   T   T
  0.1536139357909796  0.1589791400779472  0.3023590258640623   T   T   T
  0.1629808837967686  0.6676804146792621  0.3030883189570189   T   T   T
  0.4361968955189492  0.2035961144894330  0.3061738878022086   T   T   T
  0.4396651949371569  0.6989939828743016  0.2993889592598341   T   T   T
  0.6788088960084329  0.1833250615302098  0.3291305594886557   T   T   T
  0.6890085399020834  0.6900694607113276  0.3139805998465767   T   T   T
  0.9235199191283353  0.1767180481214321  0.3271770764138395   T   T   T
  0.9249844976623647  0.6772706560395219  0.3274167495923535   T   T   T
  0.2375165695672424  0.9791690365871012  0.3655564396478814   T   T   T
```

```
 0.2185666014569505  0.4881583295315110  0.3669458314110751   T   T   T
 0.4458131828653239  0.9281165997782616  0.3683105271161862   T   T   T
 0.5057904029542373  0.5619512476694446  0.3700812925470325   T   T   T
 0.3877604529910429  0.4250697838141868  0.4200107813805384   T   T   T
 0.2596339732710725  0.2644793593656222  0.4043602452432727   T   T   T
 0.4298327962171636  0.3093778547215089  0.4346569085160452   T   T   T
 0.3958222828939029  0.5868346008374331  0.4401237459278068   T   T   T
```

*C

```
   11.5352001190000006    0.0000000000000000    0.0000000000000000
   -2.8838000298000002    4.9948881704000003    0.0000000000000000
    0.0000000000000000    0.0000000000000000   32.0638008118000002
   Au   Cu   C
   24    4    1
Selective dynamics
Direct
 0.0000000000000000  0.0000000000000000  0.1559400000000011   F   F   F
 0.0000000029999967  0.5000000140000012  0.1559400000000011   F   F   F
 0.2500000000000000  0.0000000000000000  0.1559400000000011   F   F   F
 0.2500000029999967  0.5000000140000012  0.1559400000000011   F   F   F
 0.5000000000000000  0.0000000000000000  0.1559400000000011   F   F   F
 0.4999999830000021  0.5000000140000012  0.1559400000000011   F   F   F
 0.7499999590000002  0.0000000000000000  0.1559400000000011   F   F   F
 0.7499999409999987  0.5000000140000012  0.1559400000000011   F   F   F
 0.0566805252446700  0.2874732907412801  0.2296041288956087   T   T   T
 0.0623290005392298  0.7993641515997023  0.2303107746339052   T   T   T
 0.3324951473363504  0.3904964902298477  0.2318280309855105   T   T   T
 0.3453740197139238  0.9045925932526537  0.2326188104564267   T   T   T
 0.6095253606879038  0.3688268009740646  0.2313549647106556   T   T   T
 0.6043810657190798  0.8622987124858295  0.2301572075683983   T   T   T
 0.8308229618838254  0.3338603093209074  0.2559066733516352   T   T   T
 0.8295829754379256  0.8324900330191656  0.2590257048923142   T   T   T
 0.1729486679010145  0.1198147018340360  0.3027652903225189   T   T   T
 0.1608782415968314  0.6177016861485408  0.2963863524840412   T   T   T
 0.4560694478335862  0.3154349569940736  0.3006090752170564   T   T   T
 0.4710473647168344  0.8264937469878969  0.3015296102305838   T   T   T
 0.7264989393885882  0.1412869376119928  0.3365166912354317   T   T   T
 0.7075950849033693  0.6270525414366933  0.3401759986551401   T   T   T
 0.9646765602156123  0.1413095335706915  0.3399134125642858   T   T   T
 0.9571220517995471  0.6428199636075368  0.3390861903261022   T   T   T
 0.2820440853170458  0.9513189178119338  0.3578745701559067   T   T   T
 0.2318966130471694  0.4543988100499987  0.3627715325277732   T   T   T
 0.5254760248601409  0.1603775945052944  0.3688741062771838   T   T   T
 0.4775131481094426  0.5762856196198972  0.3699645253028325   T   T   T
 0.3907855054585200  0.7888966648109821  0.3699629165171999   T   T   T
```

*CH

```
   11.5352001190000006    0.0000000000000000    0.0000000000000000
   -2.8838000298000002    4.9948881704000003    0.0000000000000000
    0.0000000000000000    0.0000000000000000   32.0638008118000002
   Au   Cu   C    H
   24    4    1    1
```

```
Selective dynamics
Direct
  0.0000000000000000  0.0000000000000000  0.1559400000000011   F   F   F
  0.0000000029999967  0.5000000140000012  0.1559400000000011   F   F   F
  0.2500000000000000  0.0000000000000000  0.1559400000000011   F   F   F
  0.2500000029999967  0.5000000140000012  0.1559400000000011   F   F   F
  0.5000000000000000  0.0000000000000000  0.1559400000000011   F   F   F
  0.4999999830000021  0.5000000140000012  0.1559400000000011   F   F   F
  0.7499999590000002  0.0000000000000000  0.1559400000000011   F   F   F
  0.7499999409999987  0.5000000140000012  0.1559400000000011   F   F   F
  0.0653568700617441  0.3158521646188997  0.2294334758584637   T   T   T
  0.0673624074460550  0.8158602688108962  0.2293798322375327   T   T   T
  0.3348476352503196  0.3238519795013669  0.2350947302884069   T   T   T
  0.3335171888227744  0.8263660902241325  0.2348741633747372   T   T   T
  0.6037381836202742  0.3558565618269217  0.2294745250567912   T   T   T
  0.6091239486170485  0.8623238192115115  0.2272243594435611   T   T   T
  0.8319968698382012  0.3352840367718081  0.2562015306102622   T   T   T
  0.8315770126049382  0.8323074078396173  0.2556335381098057   T   T   T
  0.1462849404507162  0.1452249474491233  0.3003687772732436   T   T   T
  0.1492352910714463  0.6464322539800151  0.3016386556682353   T   T   T
  0.4663492716517007  0.2125176564642336  0.3029292793843025   T   T   T
  0.4714406161287731  0.7175934994430131  0.2977519684132356   T   T   T
  0.6890814422778042  0.1845439631429634  0.3339690901692053   T   T   T
  0.6899441025728879  0.6880676737520290  0.3336418616803178   T   T   T
  0.9332526756005315  0.1799944968756531  0.3367980651431927   T   T   T
  0.9303909676030627  0.6768870643692043  0.3350469094841155   T   T   T
  0.2579838595011859  0.0135192445861502  0.3580069225545577   T   T   T
  0.2126155745856700  0.4508512197681818  0.3655076002261610   T   T   T
  0.4455377625675663  0.9255181102070452  0.3686201263943047   T   T   T
  0.4493314015917219  0.4650290268899845  0.3689081593784537   T   T   T
  0.3631881231534936  0.6101813063569301  0.4009675316015295   T   T   T
  0.3650102436086616  0.6106963693827756  0.4352052111811653   T   T   T
```

*CH$_2$OH

```
    11.5352001190000006    0.0000000000000000    0.0000000000000000
    -2.8838000298000002    4.9948881704000003    0.0000000000000000
     0.0000000000000000    0.0000000000000000   32.0638008118000002
   Au   Cu   C    O    H
    24    4    1    1    3
Selective dynamics
Direct
  0.0000000000000000  0.0000000000000000  0.1559400000000011   F   F   F
  0.0000000029999967  0.5000000140000012  0.1559400000000011   F   F   F
  0.2500000000000000  0.0000000000000000  0.1559400000000011   F   F   F
  0.2500000029999967  0.5000000140000012  0.1559400000000011   F   F   F
  0.5000000000000000  0.0000000000000000  0.1559400000000011   F   F   F
  0.4999999830000021  0.5000000140000012  0.1559400000000011   F   F   F
  0.7499999590000002  0.0000000000000000  0.1559400000000011   F   F   F
  0.7499999409999987  0.5000000140000012  0.1559400000000011   F   F   F
  0.0678052844298298  0.3092232687532979  0.2295342184645568   T   T   T
  0.0665800670767775  0.8055391335236842  0.2291158455219626   T   T   T
  0.3362330661633357  0.2923894946139781  0.2336572040758299   T   T   T
  0.3352847048586559  0.7893354748307999  0.2332260023488364   T   T   T
```

```
0.6012887450368322  0.3465533071568973  0.2307409105081111   T   T   T
0.6054173844612163  0.8531226268197875  0.2289159386512659   T   T   T
0.8309355183269191  0.3288064412188398  0.2549293330024467   T   T   T
0.8318877397069162  0.8290986408334792  0.2530438386495700   T   T   T
0.1494220114448961  0.1257616862190922  0.2999057898297999   T   T   T
0.1576395991272715  0.6331727010915136  0.3020576894610155   T   T   T
0.4480364802580843  0.1454300060896828  0.3017027965637144   T   T   T
0.4642841135174837  0.6634020263403094  0.3003404278831941   T   T   T
0.6837402641470089  0.1634667476451816  0.3332067812333548   T   T   T
0.6950001573746843  0.6741482313444958  0.3318184605001923   T   T   T
0.9318435924995029  0.1654713254132153  0.3321545558830418   T   T   T
0.9305869173497447  0.6555013838279365  0.3320308739045537   T   T   T
0.2530432360819861  0.9944301749796841  0.3609989356985343   T   T   T
0.2633154901514330  0.4492172367559873  0.3599673390785498   T   T   T
0.4486992201300895  0.8930660414377490  0.3695312425671445   T   T   T
0.4679714014528391  0.4138075300896857  0.3683023266727760   T   T   T
0.2506395672537091  0.5932890000833000  0.4151517823458394   T   T   T
0.1699338452283779  0.7209213485093093  0.4105428735699883   T   T   T
0.3337205892812531  0.7212954592173311  0.4362399305797683   T   T   T
0.1918033115265263  0.3971077801311351  0.4303223816703100   T   T   T
0.0806908220845834  0.5834933942688836  0.4005092169443787   T   T   T
```

*OCH_3

```
   11.5352001190000006    0.0000000000000000    0.0000000000000000
   -2.8838000298000002    4.9948881704000003    0.0000000000000000
    0.0000000000000000    0.0000000000000000   32.0638008118000002
  Au   Cu   C    O    H
  24    4   1    1    3
Selective dynamics
Direct
0.0000000000000000  0.0000000000000000  0.1559400000000011   F   F   F
0.0000000029999967  0.5000000140000012  0.1559400000000011   F   F   F
0.2500000000000000  0.0000000000000000  0.1559400000000011   F   F   F
0.2500000029999967  0.5000000140000012  0.1559400000000011   F   F   F
0.5000000000000000  0.0000000000000000  0.1559400000000011   F   F   F
0.4999999830000021  0.5000000140000012  0.1559400000000011   F   F   F
0.7499999590000002  0.0000000000000000  0.1559400000000011   F   F   F
0.7499999409999987  0.5000000140000012  0.1559400000000011   F   F   F
0.0633328994726517  0.3148784543193992  0.2302828833548493   T   T   T
0.0645522646326743  0.8164599558420428  0.2312824540583960   T   T   T
0.3286963545017628  0.3285180089021364  0.2374140454111750   T   T   T
0.3285879077010932  0.8292505978550222  0.2372808976595585   T   T   T
0.5950536175097861  0.3457346851095838  0.2284188553570562   T   T   T
0.5964285260774733  0.8468416864146551  0.2284687342619927   T   T   T
0.8262080458868157  0.3276755120455201  0.2506809126890198   T   T   T
0.8259504200523994  0.8263656540948618  0.2511549638572985   T   T   T
0.1407404614539768  0.1486020178895989  0.3028866052190799   T   T   T
0.1407203176696092  0.6396493300050458  0.3027694298508674   T   T   T
0.4714693447616791  0.2262350629734106  0.3024903452532796   T   T   T
0.4684671251380653  0.7224325650653115  0.3024018948883612   T   T   T
0.6991361058550578  0.1997014242614864  0.3310616914595256   T   T   T
0.6983672775140797  0.7011984180344295  0.3316145150451586   T   T   T
0.9332575183532632  0.1852279181742149  0.3409026727686844   T   T   T
```

0.9351201777476144 0.6871520546743177 0.3405289235407122 T T T
0.2680392229948544 0.0219891381889864 0.3540492044182356 T T T
0.2633228288545570 0.5183789153853164 0.3585482661245221 T T T
0.4700672458316392 0.0014972984235132 0.3716095802792372 T T T
0.4683711220890267 0.4477865339831818 0.3723767765410235 T T T
0.3875258803406515 0.6548975516142752 0.4484478568831462 T T T
0.4100679238846191 0.6680406412102877 0.4037441234488114 T T T
0.2933212628583405 0.4727855687915732 0.4562307954305149 T T T
0.3823989834885361 0.8319568808898200 0.4582407556081953 T T T
0.4716446902782030 0.6531405750681423 0.4641655160466157 T T T

*CH_2

11.5352001190000006 0.0000000000000000 0.0000000000000000
-2.8838000298000002 4.9948881704000003 0.0000000000000000
0.0000000000000000 0.0000000000000000 32.0638008118000002
Au Cu C H
24 4 1 2
Selective dynamics
Direct
0.0000000000000000 0.0000000000000000 0.1559400000000011 F F F
0.0000000029999967 0.5000000140000012 0.1559400000000011 F F F
0.2500000000000000 0.0000000000000000 0.1559400000000011 F F F
0.2500000029999967 0.5000000140000012 0.1559400000000011 F F F
0.5000000000000000 0.0000000000000000 0.1559400000000011 F F F
0.4999999830000021 0.5000000140000012 0.1559400000000011 F F F
0.7499999590000002 0.0000000000000000 0.1559400000000011 F F F
0.7499999409999987 0.5000000140000012 0.1559400000000011 F F F
0.0604609699551602 0.3189237217627152 0.2286768894083738 T T T
0.0613403544609298 0.8150332148162152 0.2295119521007912 T T T
0.3310261254460870 0.3709993343842826 0.2360257946802223 T T T
0.3281998008586162 0.8705378127036481 0.2365654345003287 T T T
0.5998180706774827 0.3429427703189601 0.2289468796193606 T T T
0.6022592549545728 0.8440642714169342 0.2283377446835990 T T T
0.8254394427083026 0.3178588692655870 0.2554710749409878 T T T
0.8268638103311862 0.8173991627586086 0.2550913645472004 T T T
0.1342879370124447 0.1547125940975411 0.2993041579779444 T T T
0.1405142808996037 0.6542021362570535 0.3024818627644810 T T T
0.4629005002768112 0.2434971182109981 0.3023073307258927 T T T
0.4653115526423976 0.7472644576875856 0.3004478644830878 T T T
0.6830300744699150 0.2126980319744244 0.3345199388824702 T T T
0.6838627022206659 0.7106550032575987 0.3337136365330539 T T T
0.9206654611879005 0.1963271768824490 0.3349609721345002 T T T
0.9249463444076341 0.6995980146446579 0.3366322843135737 T T T
0.2484586634466638 0.0345166473826866 0.3559123790900630 T T T
0.2585863001231024 0.5064958643710670 0.3562271191320205 T T T
0.4397300522634632 0.9767211525219518 0.3713713384326477 T T T
0.4495041730015170 0.4559023577343291 0.3728346728142972 T T T
0.3759623127683598 0.6510638217517249 0.4061710400507398 T T T
0.2900860101774446 0.5724945118818497 0.4276764855024749 T T T
0.4668127193749053 0.7213052889698013 0.4261551270116222 T T T

*$HOCH_3$

```
   11.5352001190000006    0.0000000000000000    0.0000000000000000
   -2.8838000298000002    4.9948881704000003    0.0000000000000000
    0.0000000000000000    0.0000000000000000   32.0638008118000002
  Au   Cu   C    O    H
   24    4    1    1    4
Selective dynamics
Direct
  0.0000000000000000  0.0000000000000000  0.1558800106620666   F   F   F
  0.0000000048476565  0.5000000192346832  0.1558800106620666   F   F   F
  0.2500000000064873  0.0000000000000000  0.1558800106620666   F   F   F
  0.2500000047676068  0.5000000192346832  0.1558800106620666   F   F   F
  0.5000000000129816  0.0000000000000000  0.1558800106620666   F   F   F
  0.4999999841789133  0.5000000192346832  0.1558800106620666   F   F   F
  0.7500000206146566  0.0000000000000000  0.1558800106620666   F   F   F
  0.7500000460574938  0.5000000192346832  0.1558800106620666   F   F   F
  0.0587460722019199  0.3125068605188883  0.2289250206612696   T   T   T
  0.0589249061344482  0.8050966592938460  0.2289927091027342   T   T   T
  0.3262314322279207  0.3215210758712608  0.2373156848431729   T   T   T
  0.3222626867644657  0.8164791237530931  0.2355379960652101   T   T   T
  0.5960917685995768  0.3487183256376613  0.2290008563374668   T   T   T
  0.5954733714550232  0.8481311834599269  0.2291468890484256   T   T   T
  0.8220118376652837  0.3241536390110837  0.2554524150429288   T   T   T
  0.8241486079333603  0.8264446672406456  0.2533562861047584   T   T   T
  0.1217772076054957  0.1207553560196786  0.2990147391302067   T   T   T
  0.1367167412205887  0.6339540649682364  0.3026002231207335   T   T   T
  0.4589814137131605  0.1978837377189744  0.3013854824942488   T   T   T
  0.4600913635552467  0.7078157087276633  0.3021666324629316   T   T   T
  0.6782565288636268  0.1781431693914160  0.3354631481138044   T   T   T
  0.6853168876074829  0.6849191360171477  0.3321604373172923   T   T   T
  0.9189318850404768  0.1736371428371616  0.3368157565179561   T   T   T
  0.9190671274078986  0.6647551810319615  0.3368189692775295   T   T   T
  0.2540961096146909  0.0283633214620099  0.3529673159498881   T   T   T
  0.2567531194980073  0.4758257038698491  0.3533435654935713   T   T   T
  0.4376969982724894  0.9384470399075928  0.3722012464822796   T   T   T
  0.4572164944293258  0.4424530362990482  0.3697760984345593   T   T   T
  0.3728627953949389  0.6343331308187320  0.4570451160578350   T   T   T
  0.3673446895632443  0.8366486441968489  0.4314211087082155   T   T   T
  0.2799455155057672  0.4407265525207552  0.4538940059222473   T   T   T
  0.3886791590865301  0.7010579307472025  0.4896999797705433   T   T   T
  0.4585321526694007  0.6173327501538672  0.4461369248277426   T   T   T
  0.2818641013841285  0.8283975986537452  0.4364322714642486   T   T   T
```

*CH_3

```
   11.5352001190000006    0.0000000000000000    0.0000000000000000
   -2.8838000298000002    4.9948881704000003    0.0000000000000000
    0.0000000000000000    0.0000000000000000   32.0638008118000002
  Au   Cu   C    H
   24    4    1    3
Selective dynamics
Direct
  0.0000000000000000  0.0000000000000000  0.1559400000000011   F   F   F
  0.0000000029999967  0.5000000140000012  0.1559400000000011   F   F   F
  0.2500000000000000  0.0000000000000000  0.1559400000000011   F   F   F
```

0.2500000029999967	0.5000000140000012	0.1559400000000011	F	F	F
0.5000000000000000	0.0000000000000000	0.1559400000000011	F	F	F
0.4999999830000021	0.5000000140000012	0.1559400000000011	F	F	F
0.7499999590000002	0.0000000000000000	0.1559400000000011	F	F	F
0.7499999409999987	0.5000000140000012	0.1559400000000011	F	F	F
0.0532367321154030	0.2987727586943543	0.2291020641698651	T	T	T
0.0559356407640512	0.8052476150065629	0.2291630063902849	T	T	T
0.3189345875136392	0.3089034360963806	0.2347368752408178	T	T	T
0.3189759586574709	0.8140370427015485	0.2345491558965710	T	T	T
0.5902080184067326	0.3403053828011693	0.2296653522427309	T	T	T
0.5997948784913457	0.8502526737569045	0.2273662129300924	T	T	T
0.8208483254275329	0.3230648426394404	0.2544739522959237	T	T	T
0.8192443531420597	0.8167250360452487	0.2555279738280415	T	T	T
0.1305954544989909	0.1284053918683072	0.3018152379201061	T	T	T
0.1249928714662252	0.6175622175248996	0.2998290699937441	T	T	T
0.4494295019506494	0.1930854484167453	0.3028912919370557	T	T	T
0.4510985498488305	0.6956305142277797	0.2970593576641425	T	T	T
0.6726821363387800	0.1672605989277148	0.3325068623925053	T	T	T
0.6700148259223662	0.6681942163370578	0.3335158238336416	T	T	T
0.9102075391118061	0.1542905260155253	0.3344298246845244	T	T	T
0.9106949943636770	0.6576059224490408	0.3339094810189800	T	T	T
0.2458525512612911	0.9774011849778638	0.3545277145797615	T	T	T
0.2295142146847900	0.4886576108442265	0.3591054339436643	T	T	T
0.4383419867146371	0.9085886146270383	0.3681657068617242	T	T	T
0.4295255146082451	0.4439369273663078	0.3677246323365981	T	T	T
0.3490323486910269	0.5915928722897920	0.4137175165100962	T	T	T
0.2457400630966972	0.4911853707891982	0.4262061799705173	T	T	T
0.4011006530252073	0.7912911538141942	0.4291072488103957	T	T	T
0.3958852589476884	0.4813328151756097	0.4279893934332968	T	T	T

Coordinates for transition state calculations.

Ag-Cu surface

*CO to *CHO

```
   11.5560998917000006    0.0000000000000000    0.0000000000000000
   -2.8890500068999998    5.0039813974999996    0.0000000000000000
    0.0000000000000000    0.0000000000000000   32.0765991211000028
  Ag   Cu   C    O    H
 24    4    1    2    2
Selective dynamics
Direct
 0.0000000000000000  0.0000000000000000  0.1558800109800487   F   F   F
 0.0000000049910014  0.5000000190138536  0.1558800109800487   F   F   F
 0.2500000000003836  0.0000000000000000  0.1558800109800487   F   F   F
 0.2500000049862759  0.5000000190138536  0.1558800109800487   F   F   F
 0.5000000000007672  0.0000000000000000  0.1558800109800487   F   F   F
 0.4999999840105426  0.5000000190138536  0.1558800109800487   F   F   F
 0.7500000209772455  0.0000000000000000  0.1558800109800487   F   F   F
 0.7500000460033568  0.5000000190138536  0.1558800109800487   F   F   F
 0.0762813402022019  0.3426611049023559  0.2276943538573540   T   T   T
 0.0778439143726459  0.8363891982696718  0.2286567911702983   T   T   T
 0.3371393206740998  0.3322255296839042  0.2289006790142780   T   T   T
 0.3328758162670230  0.8226638536832356  0.2282265023703077   T   T   T
 0.5990040436689605  0.3741215059037077  0.2314706288172513   T   T   T
 0.5935831598525394  0.8718389454517269  0.2346849333256188   T   T   T
 0.8367758314313525  0.3525760969565020  0.2466056067392613   T   T   T
 0.8410699143781681  0.8580329242355059  0.2414424981338913   T   T   T
 0.1497833209826661  0.1765354636469250  0.2975922714694368   T   T   T
 0.1591939357455320  0.6786771875758632  0.3019990111415057   T   T   T
 0.4236961929096111  0.1520029100800707  0.2976549552901304   T   T   T
 0.4186862988077132  0.6462048899949877  0.3041392158067242   T   T   T
 0.6622252120619002  0.1865485545385319  0.3250089754125130   T   T   T
 0.6783946715571534  0.7064539930395458  0.3151814178212948   T   T   T
 0.9236347873444951  0.2009124105917457  0.3234708729184958   T   T   T
 0.9240879355833299  0.6963193712677327  0.3231265200094162   T   T   T
 0.2433068123879795  0.0498281161022330  0.3598828837896807   T   T   T
 0.2167450673468743  0.4571643310095354  0.3652550650603980   T   T   T
 0.4246764993930351  0.9370208060976838  0.3692176994515833   T   T   T
 0.4782591500599113  0.4183760077208210  0.3676598722987113   T   T   T
 0.4564685127530554  0.4347524971669406  0.4270505030653024   T   T   T
 0.4949127410515705  0.3578927581578924  0.4554860375219141   T   T   T
 0.3210824509734509  0.6747591477950141  0.4142830172874756   T   T   T
 0.3910955628980258  0.5573545724572679  0.4273524030056701   T   T   T
 0.2787044502466864  0.7235939601095500  0.4363341651416355   T   T   T
```

*CO to *COH

```
   11.5560998917000006    0.0000000000000000    0.0000000000000000
   -2.8890500068999998    5.0039813974999996    0.0000000000000000
    0.0000000000000000    0.0000000000000000   32.0765991211000028
  Ag   Cu   C    O    H
 24    4    1    2    2
Selective dynamics
Direct
 0.0000000000000000  0.0000000000000000  0.1558800106620666   F   F   F
```

0.0000000048476573 0.5000000192346832 0.1558800106620666 F F F
0.2500000000064873 0.0000000000000000 0.1558800106620666 F F F
0.2500000047676068 0.5000000192346832 0.1558800106620666 F F F
0.5000000000129815 0.0000000000000000 0.1558800106620666 F F F
0.4999999841789133 0.5000000192346832 0.1558800106620666 F F F
0.7500000206146550 0.0000000000000000 0.1558800106620666 F F F
0.7500000460574843 0.5000000192346832 0.1558800106620666 F F F
0.0844289079669767 0.3331987860311140 0.2316629856432306 T T T
0.0855431105028034 0.8369818808854217 0.2320753271596508 T T T
0.3369033837294588 0.3442412136705026 0.2280946797029852 T T T
0.3372832097599874 0.8427747652817719 0.2277340568261812 T T T
0.6013247858922609 0.3458517734100268 0.2320646087203855 T T T
0.6018918831717530 0.8449775856988297 0.2325489759862009 T T T
0.8441451070418423 0.3418112746589873 0.2435252448973507 T T T
0.8428143290915322 0.8399871865367711 0.2450284235912439 T T T
0.1652598658861704 0.1658635515372996 0.3058080494221506 T T T
0.1666269678733254 0.6639690060726378 0.3054585357429936 T T T
0.4211426763487569 0.1763631752353839 0.2982806194659485 T T T
0.4249609221804616 0.6824826518327676 0.2988715185093623 T T T
0.6716015184825742 0.1711085430943826 0.3192257715909133 T T T
0.6651368308320784 0.6619998678781771 0.3225346720685888 T T T
0.9263430045575115 0.1767892262554701 0.3233656452755672 T T T
0.9257896264431500 0.6756947468160873 0.3229413664583801 T T T
0.2146920505771807 0.9831246151636966 0.3737771165439133 T T T
0.2212610708961113 0.4542562710256433 0.3730013096069726 T T T
0.4398454506276723 0.9549910997218566 0.3663527749616410 T T T
0.4312775801234150 0.4057219230045638 0.3690128831224830 T T T
0.4069545900033498 0.6445702452061771 0.4040977383808603 T T T
0.3945826257916962 0.7015716394754109 0.4418155147684397 T T T
0.1864286637858088 0.6946549140670238 0.4175269247335601 T T T
0.2879401484082360 0.7529678750528280 0.4412742655858748 T T T
0.0890231953486605 0.5984713707029448 0.4241019258923446 T T T

*CHO to *CHOH
11.5560998917000006 0.0000000000000000 0.0000000000000000
-2.8890500068999998 5.0039813974999996 0.0000000000000000
0.0000000000000000 0.0000000000000000 32.0765991211000028
Ag Cu C O H
24 4 1 2 3
Selective dynamics
Direct
0.0000000000000000 0.0000000000000000 0.1559399932678570 F F F
0.0000000048476636 0.5000000192346830 0.1559399932678570 F F F
0.2500000000064870 0.0000000000000000 0.1559399932678570 F F F
0.2500000047676070 0.5000000192346830 0.1559399932678570 F F F
0.5000000000129819 0.0000000000000000 0.1559399932678570 F F F
0.4999999841789130 0.5000000192346830 0.1559399932678570 F F F
0.7500000206146570 0.0000000000000000 0.1559399932678570 F F F
0.7500000460574729 0.5000000192346830 0.1559399932678570 F F F
0.0786873827692146 0.3567063964169210 0.2289322296918630 T T T
0.0798738673932787 0.8486323770968480 0.2303682071721520 T T T
0.3374406587157360 0.3317956928284120 0.2285780301168110 T T T
0.3362615114844720 0.8350786082112720 0.2289966864439180 T T T
0.6001222137737730 0.3762765721179790 0.2332077433308300 T T T
0.6053361270348619 0.8849902331694740 0.2313183086685480 T T T

0.8405484072456230 0.3706433845103600 0.2486890357164950 T T T
0.8445054059420570 0.8689404371364080 0.2433205472636360 T T T
0.1615486136642260 0.2054257080697620 0.2988947960515420 T T T
0.1705220499205750 0.7167416673020780 0.3045101511959970 T T T
0.4332691342169030 0.1796346397912190 0.3023220980648500 T T T
0.4312464545434950 0.6740339807925050 0.2975398304777460 T T T
0.6639082867350971 0.1559952856512320 0.3254752319301470 T T T
0.6819369241380860 0.6774160776486220 0.3176609008530030 T T T
0.9263208256096140 0.2108224404446000 0.3238099451806380 T T T
0.9404206283174550 0.7250748104190660 0.3297900084581640 T T T
0.2547313706844270 0.0962987744896427 0.3656199998629580 T T T
0.1566396455255650 0.4215132383229320 0.3684167206859620 T T T
0.3813780768852210 0.8453036176047050 0.3654239870739860 T T T
0.4627645501147170 0.5117956178303500 0.3687951645268040 T T T
0.3856003016496560 0.2693180474715080 0.4172599294404520 T T T
0.3285733631438100 0.2983852999523390 0.4492219506623410 T T T
0.2336676398179330 0.5761674848825870 0.4246670664872130 T T T
0.4463698780183160 0.1722436316224330 0.4248343270422300 T T T
0.2708676502433800 0.4569072457452840 0.4394787080869460 T T T
0.1976831099482670 0.6515608631121170 0.4443860684733810 T T T

*CHO to *CH_2O
11.5560998917000006 0.0000000000000000 0.0000000000000000
-2.8890500068999998 5.0039813974999996 0.0000000000000000
0.0000000000000000 0.0000000000000000 32.0765991211000028
Ag Cu C O H
24 4 1 2 3
Selective dynamics
Direct
0.0000000000000000 0.0000000000000000 0.1558800106620666 F F F
0.0000000048476603 0.5000000192346832 0.1558800106620666 F F F
0.2500000000064873 0.0000000000000000 0.1558800106620666 F F F
0.2500000047676068 0.5000000192346832 0.1558800106620666 F F F
0.5000000000129815 0.0000000000000000 0.1558800106620666 F F F
0.4999999841789133 0.5000000192346832 0.1558800106620666 F F F
0.7500000206146550 0.0000000000000000 0.1558800106620666 F F F
0.7500000460574828 0.5000000192346832 0.1558800106620666 F F F
0.0785437264822345 0.3516132235871297 0.2290916873583172 T T T
0.0803302859902844 0.8471851773382542 0.2291289342776367 T T T
0.3406841028173340 0.3476297509510228 0.2283352588124183 T T T
0.3393583429621742 0.8421791823472402 0.2299151963287396 T T T
0.6018837649829943 0.3608559397283803 0.2314131039552460 T T T
0.5979705115541933 0.8595520438171845 0.2347199064349011 T T T
0.8397181074611452 0.3485010428389354 0.2484916338105404 T T T
0.8435006072089239 0.8544900052951722 0.2435487030088821 T T T
0.1719642452179193 0.2159714424389406 0.2987880095680030 T T T
0.1729890843049238 0.7258221931692939 0.3022861789514649 T T T
0.4291254991498387 0.2073028357508093 0.3000029198672660 T T T
0.4271038055434841 0.6988403088042912 0.3054783213687757 T T T
0.6685507836577256 0.2112690709019224 0.3244937310625581 T T T
0.6832792744040964 0.7269399865088734 0.3173861316151339 T T T
0.9412368660403828 0.2182469834858655 0.3276729897470095 T T T
0.9335221771436045 0.7110398940044683 0.3253030265845783 T T T
0.1643492351030602 0.9960840649278169 0.3694721057869009 T T T
0.1809387187023358 0.4730523140017713 0.3670167122851437 T T T

```
 0.4378264405934253  0.9898356082080826  0.3693694770961897   T   T   T
 0.4711960830344822  0.4594213903272743  0.3698778387424306   T   T   T
 0.3796966409477927  0.2995183523981115  0.4221943122596797   T   T   T
 0.2581942832492669  0.0952450613897528  0.4231308392762630   T   T   T
 0.3224462507154823  0.6885223531657260  0.4115558618166936   T   T   T
 0.4411029301991108  0.3023440811690485  0.4492294323809152   T   T   T
 0.3677890586758331  0.5489548754668845  0.4168379954620020   T   T   T
 0.2917478316304457  0.7489053103395271  0.4356286116109072   T   T   T
```

*COH to *C

```
   11.5560998917000006    0.0000000000000000    0.0000000000000000
   -2.8890500068999998    5.0039813974999996    0.0000000000000000
    0.0000000000000000    0.0000000000000000   32.0765991211000028
  Ag   Cu   C    O    H
 24   4   1   2   3
Selective dynamics
Direct
 0.0000000000000000  0.0000000000000000  0.1558800106620666   F   F   F
 0.0000000048476565  0.5000000192346833  0.1558800106620666   F   F   F
 0.2500000000064873  0.0000000000000000  0.1558800106620666   F   F   F
 0.2500000047676068  0.5000000192346833  0.1558800106620666   F   F   F
 0.5000000000129816  0.0000000000000000  0.1558800106620666   F   F   F
 0.4999999841789136  0.5000000192346833  0.1558800106620666   F   F   F
 0.7500000206146566  0.0000000000000000  0.1558800106620666   F   F   F
 0.7500000460574938  0.5000000192346833  0.1558800106620666   F   F   F
 0.0495395714948423  0.3177142881575425  0.2292334776542511   T   T   T
 0.0622689236780902  0.8233591982103685  0.2315636592947538   T   T   T
 0.3381004754892427  0.2949915653599788  0.2301783026327604   T   T   T
 0.3135242776412920  0.7742350762940678  0.2318629571586555   T   T   T
 0.6039629096936742  0.3667818448510462  0.2306068181176406   T   T   T
 0.6028798673308289  0.8697838369329750  0.2291271029030371   T   T   T
 0.8264238050476352  0.3413338119334423  0.2582790557335657   T   T   T
 0.8276657026015513  0.8370710361608447  0.2553319296284408   T   T   T
 0.1593819831390250  0.2205962136377778  0.2967297267849613   T   T   T
 0.1684328692232241  0.7584311122232082  0.3096093177723829   T   T   T
 0.4403443603051163  0.1191951552541146  0.2988452974522617   T   T   T
 0.4476483139036531  0.6342518206047458  0.2975039376172407   T   T   T
 0.6856687916960614  0.3092277759699344  0.3382719116239665   T   T   T
 0.7022033351130905  0.8260178903971955  0.3331626106511001   T   T   T
 0.9442309295385507  0.3015042396016706  0.3366636293634678   T   T   T
 0.9378827403137968  0.7917511571941145  0.3362437360678368   T   T   T
 0.2484974330122370  0.1025469878990843  0.3735287207521857   T   T   T
 0.1909264311135639  0.4688729240784593  0.3650365534719641   T   T   T
 0.4580396990425490  0.8776006231137596  0.3666599601403089   T   T   T
 0.4952564260318128  0.4132238852832509  0.3653230077801817   T   T   T
 0.3565081234943649  0.4897704697915751  0.3792034977610500   T   T   T
 0.4010735819572430  0.7458171265146826  0.4267935311434272   T   T   T
 0.2038039313301013  0.8391515088428342  0.4241108371280939   T   T   T
 0.4727057291180992  0.7481522657932874  0.4432923177703933   T   T   T
 0.1708564879516900  0.8837841290025054  0.4490948534046447   T   T   T
 0.2826584388054427  0.8157744069762495  0.4320272390137139   T   T   T
```

*COH to *CHOH

```
   11.5560998917000006    0.0000000000000000    0.0000000000000000
```

-2.8890500068999998 5.0039813974999996 0.0000000000000000
0.0000000000000000 0.0000000000000000 32.0765991211000028
Ag Cu C O H
24 4 1 2 3
Selective dynamics
Direct
0.0000000000000000 0.0000000000000000 0.1559399996384713 F F F
0.0000000033847972 0.5000000135738748 0.1559399996384713 F F F
0.2500000000216716 0.0000000000000000 0.1559399996384713 F F F
0.2500000034064696 0.5000000135738748 0.1559399996384713 F F F
0.5000000000433431 0.0000000000000000 0.1559399996384713 F F F
0.4999999827089566 0.5000000135738748 0.1559399996384713 F F F
0.7499999586266384 0.0000000000000000 0.1559399996384713 F F F
0.7499999413789379 0.5000000135738748 0.1559399996384713 F F F
0.0625283213867748 0.3178781122916352 0.2306661132262430 T T T
0.0593952104729510 0.8098202997314817 0.2300645661637318 T T T
0.3329482718026912 0.3616011171929244 0.2322558378725280 T T T
0.3199292596690820 0.8530377362280547 0.2335455107063737 T T T
0.5953679215653721 0.3424089297363468 0.2308009848813214 T T T
0.6065821747513942 0.8522768153418104 0.2273956956758353 T T T
0.8276205655775855 0.3259027698802612 0.2555528031434121 T T T
0.8267959060190648 0.8235261215173363 0.2553976533949679 T T T
0.1497969392462120 0.1510774070806237 0.2994416075721947 T T T
0.1510447880862374 0.6560350176063107 0.3052532868591447 T T T
0.4411086632570864 0.2284451141021977 0.3031372311688977 T T T
0.4567982476393784 0.7510629427812898 0.2955047166612472 T T T
0.6817817088007249 0.1976496735372522 0.3323093979888397 T T T
0.6742687577753868 0.6927392943916598 0.3321972413669904 T T T
0.9281504268060803 0.1856825621046885 0.3361161316822345 T T T
0.9213330333952129 0.6849425067622869 0.3326791665842073 T T T
0.2284019855932749 0.0219319653885705 0.3651593613083499 T T T
0.1911894084236065 0.4137870371009381 0.3678519210329911 T T T
0.4438873944009928 0.9588048757208139 0.3671944953903489 T T T
0.4263766511299616 0.4985869846977436 0.3645462300468188 T T T
0.3477965340315887 0.6388518021865354 0.4044257456548724 T T T
0.3809875615926306 0.6227997269728193 0.4458671038066991 T T T
0.2020388948302262 0.8679504864361234 0.4216430930410863 T T T
0.4592156971197689 0.5991717363999708 0.4468974347068862 T T T
0.2626011765295320 0.7366466773958419 0.4115140898180596 T T T
0.2608306470167497 0.0080655478078784 0.4411194352768738 T T T

*CH_2O to *OCH_3
11.5560998917000006 0.0000000000000000 0.0000000000000000
-2.8890500068999998 5.0039813974999996 0.0000000000000000
0.0000000000000000 0.0000000000000000 32.0765991211000028
Ag Cu C O H
24 4 1 2 4
Selective dynamics
Direct
0.0000000000000000 0.0000000000000000 0.1558800106620666 F F F
0.0000000048476573 0.5000000192346832 0.1558800106620666 F F F
0.2500000000064873 0.0000000000000000 0.1558800106620666 F F F
0.2500000047676068 0.5000000192346832 0.1558800106620666 F F F
0.5000000000129815 0.0000000000000000 0.1558800106620666 F F F

0.4999999841789133 0.5000000192346832 0.1558800106620666 F F F
0.7500000206146550 0.0000000000000000 0.1558800106620666 F F F
0.7500000460574843 0.5000000192346832 0.1558800106620666 F F F
0.0759429881569005 0.3340341960358960 0.2281080090994355 T T T
0.0740174681215391 0.8348357690634938 0.2276792918798084 T T T
0.3349902942288593 0.3218711581388818 0.2306579014871064 T T T
0.3364341404732470 0.8224855122145649 0.2294408681902650 T T T
0.5938543966631525 0.3559036930191545 0.2328438807142303 T T T
0.5910535358197805 0.8553320095439858 0.2372813172755242 T T T
0.8363717133828372 0.3451941577061410 0.2458475905383759 T T T
0.8375596613276423 0.8462374859366769 0.2427284490679162 T T T
0.1511713975125600 0.1691784660707245 0.3004982258009278 T T T
0.1512424827351493 0.6749096952397077 0.2977834705072027 T T T
0.4224467491618126 0.1430538235538915 0.3023050519276469 T T T
0.4119846040642769 0.6312448433065642 0.3062544019198832 T T T
0.6699413954322788 0.1940408446042866 0.3241720936405038 T T T
0.6751924053442491 0.6969988103846920 0.3167782099553739 T T T
0.9216212980840498 0.1918295214112091 0.3243264893766192 T T T
0.9262497868991290 0.6943269888681493 0.3278610240719620 T T T
0.2401384576901307 0.9910039766505025 0.3633096054923916 T T T
0.2111479261680492 0.5155620015658796 0.3627795175494060 T T T
0.4404668006275386 0.9312668419298055 0.3738706851288967 T T T
0.4698001179269456 0.4092530207687863 0.3712568467118543 T T T
0.4122700049716936 0.5948218684407057 0.4491313735752628 T T T
0.4721089012406790 0.6826003535570688 0.4123641024124876 T T T
0.1801433395787084 0.2380215718583726 0.4108814490853207 T T T
0.4329382239151330 0.4548251053048466 0.4662594284577803 T T T
0.3835841602705699 0.7229947402874325 0.4661917655862226 T T T
0.0922312150825106 0.1574962126867806 0.4244158740488806 T T T
0.2569979788042432 0.3488184120227260 0.4326040450071028 T T T

*CHOH to *CH_2OH
11.5560998917000006 0.0000000000000000 0.0000000000000000
-2.8890500068999998 5.0039813974999996 0.0000000000000000
0.0000000000000000 0.0000000000000000 32.0765991211000028
Ag Cu C O H
24 4 1 2 4
Selective dynamics
Direct
0.0000000000000000 0.0000000000000000 0.1558800106620666 F F F
0.0000000048476565 0.5000000192346832 0.1558800106620666 F F F
0.2500000000064873 0.0000000000000000 0.1558800106620666 F F F
0.2500000047676068 0.5000000192346832 0.1558800106620666 F F F
0.5000000000129816 0.0000000000000000 0.1558800106620666 F F F
0.4999999841789133 0.5000000192346832 0.1558800106620666 F F F
0.7500000206146565 0.0000000000000000 0.1558800106620666 F F F
0.7500000460574939 0.5000000192346832 0.1558800106620666 F F F
0.0928590350915200 0.3623939627290703 0.2342163318769298 F F F
0.0940715460700796 0.8670793081134270 0.2363978726007726 F F F
0.3404863129673932 0.3662366299950928 0.2282096151318495 F F F
0.3412016819652002 0.8626910602650004 0.2286452133571288 F F F
0.6009845330321649 0.3475897681482162 0.2318247252812434 F F F
0.5991279001152545 0.8442160339404983 0.2337492987834537 F F F
0.8493064783021750 0.3616010165744596 0.2372066210694597 F F F
0.8451628736435457 0.8550558235952110 0.2414914531327280 F F F

```
 0.1761102101501963  0.2090930583666270  0.3129080414887382   F   F   F
 0.1714140111058377  0.6931515882834614  0.3102716593245435   F   F   F
 0.4223556806757467  0.2148534853652702  0.2984392800384363   F   F   F
 0.4245708482136334  0.7188936640929574  0.3024283349481094   F   F   F
 0.6873189246284663  0.1783275483616430  0.3121756603383892   F   F   F
 0.6825829617906023  0.6700995209178914  0.3186018926377135   F   F   F
 0.9302294097776233  0.1880684633275755  0.3175286735600512   F   F   F
 0.9333263692268725  0.6942520205438628  0.3213743505240882   F   F   F
 0.2797126820480394  0.0455819727002788  0.3718042169119264   F   F   F
 0.2940331593482025  0.6220696936094736  0.3770796888623966   F   F   F
 0.4973036829509520  0.0725000223828648  0.3656806472202795   F   F   F
 0.4736364584240774  0.5053766424968417  0.3663765808896740   F   F   F
 0.1974432369392469  0.9372727040309444  0.4260253386368228   T   T   T
 0.0612342194148802  0.7793147188407689  0.4278756775170083   T   T   T
 0.2623305553551148  0.4460975532627298  0.4334218951885143   T   T   T
 0.2377326326720713  0.2253816364098925  0.4286233817778757   T   T   T
 0.2472454273664275  0.8716144180485729  0.4486443680515717   T   T   T
 0.3458408093983174  0.5322335384100330  0.4491810627422347   T   T   T
 0.0358396135660662  0.6207895314087825  0.4450099442794681   T   T   T

*CHOH to *CH
   11.5560998917000006    0.0000000000000000    0.0000000000000000
   -2.8890500068999998    5.0039813974999996    0.0000000000000000
    0.0000000000000000    0.0000000000000000   32.0765991211000028
  Ag   Cu   C    O    H
 24   4   1   2   4
Selective dynamics
Direct
 0.0000000000000000  0.0000000000000000  0.1558800109999959   F   F   F
 0.0000000050000038  0.5000000189999980  0.1558800109999959   F   F   F
 0.2499999999999861  0.0000000000000000  0.1558800109999959   F   F   F
 0.2500000049999968  0.5000000189999980  0.1558800109999959   F   F   F
 0.4999999999999722  0.0000000000000000  0.1558800109999959   F   F   F
 0.4999999839999709  0.5000000189999980  0.1558800109999959   F   F   F
 0.7500000209999972  0.0000000000000000  0.1558800109999959   F   F   F
 0.7500000459999566  0.5000000189999980  0.1558800109999959   F   F   F
 0.0728759992055564  0.3162410398381366  0.2288827961474949   T   T   T
 0.0735098321830800  0.8260301901934509  0.2289941687123954   T   T   T
 0.3305786177815397  0.3526394488036685  0.2296558936515088   T   T   T
 0.3335030723687143  0.8548827283506291  0.2295079825325913   T   T   T
 0.5956576302840212  0.3283284629096720  0.2326168248075614   T   T   T
 0.5929580336119046  0.8233487379826022  0.2362900561527082   T   T   T
 0.8376162699873342  0.3282448615446459  0.2446249114655644   T   T   T
 0.8348227695354231  0.8232083947824345  0.2466676396628946   T   T   T
 0.1536668830836661  0.1497893812960687  0.3033926482058710   T   T   T
 0.1470263908987843  0.6450207877009300  0.2984107923751516   T   T   T
 0.4228818788280051  0.2172072174922461  0.3001172748250838   T   T   T
 0.4162979083278496  0.7158823840404632  0.3033815155788879   T   T   T
 0.6723121566290803  0.1793279823355205  0.3199539107955086   T   T   T
 0.6644345659791734  0.6619131800115988  0.3254836192421229   T   T   T
 0.9175088581761136  0.1615647702681013  0.3247164213033746   T   T   T
 0.9273841092340018  0.6730089077049088  0.3300290670442034   T   T   T
 0.2360971702779563 -0.0359920223001441  0.3651785950764193   T   T   T
 0.1964931524301455  0.4574024477083717  0.3675096997729546   T   T   T
 0.4673187684458424  1.0744896966988320  0.3740606206542337   T   T   T
```

```
0.4151877998851668  0.4417435994246754  0.3707413747414320   T   T   T
0.2977928205951458  0.7693438807816714  0.4008928959748889   T   T   T
0.4610461054497432  0.7645887145396593  0.4058820581974860   T   T   T
0.3979045766131543  0.5964858732695457  0.4876102126375221   T   T   T
0.2846043149559028  0.7711210907894103  0.4349193769071393   T   T   T
0.4626765209242210  0.7478846708475910  0.4366341922522115   T   T   T
0.3246242455107120  0.4311322572895390  0.4761922458559031   T   T   T
0.4537787540155640  0.5425355683849780  0.5026318167581240   T   T   T
```

*CH_2OH to *$HOCH_3$

```
   11.5560998917000006    0.0000000000000000    0.0000000000000000
   -2.8890500068999998    5.0039813974999996    0.0000000000000000
    0.0000000000000000    0.0000000000000000   32.0765991211000028
  Ag   Cu   C    O    H
 24    4    1    2    5
Selective dynamics
Direct
 0.0000000000000000  0.0000000000000000  0.1558800106620666   F   F   F
 0.0000000048476565  0.5000000192346832  0.1558800106620666   F   F   F
 0.2500000000064873  0.0000000000000000  0.1558800106620666   F   F   F
 0.2500000047676068  0.5000000192346832  0.1558800106620666   F   F   F
 0.5000000000129816  0.0000000000000000  0.1558800106620666   F   F   F
 0.4999999841789133  0.5000000192346832  0.1558800106620666   F   F   F
 0.7500000206146565  0.0000000000000000  0.1558800106620666   F   F   F
 0.7500000460574939  0.5000000192346832  0.1558800106620666   F   F   F
 0.0746981763846863  0.3012636631015689  0.2277379271935941   T   T   T
 0.0761656996121704  0.8083638164295400  0.2289343675225226   T   T   T
 0.3346708753894595  0.3193649882991572  0.2304744474263297   T   T   T
 0.3361888251052650  0.8200350603608769  0.2299967957694503   T   T   T
 0.5940764858081928  0.3322981000443597  0.2343056043834049   T   T   T
 0.5913001107798288  0.8264297375351015  0.2380880901578488   T   T   T
 0.8363481595192286  0.3218973800981029  0.2440462801908694   T   T   T
 0.8354767736964728  0.8212554102630107  0.2459815974036342   T   T   T
 0.1493193324355050  0.1133383034263161  0.3024967831158815   T   T   T
 0.1387070602026705  0.5976760566411093  0.2985467615368867   T   T   T
 0.4168455276219813  0.1393549153208113  0.3031801481569739   T   T   T
 0.4065670646716226  0.6309318574670880  0.3062935961776337   T   T   T
 0.6608860572477608  0.1277729254552123  0.3224040224748395   T   T   T
 0.6506996356367125  0.6189266940917204  0.3274424597101763   T   T   T
 0.9108162427942400  0.1245492858086945  0.3250718737289169   T   T   T
 0.9122050030392558  0.6307718700314577  0.3254450662527502   T   T   T
 0.2193799129921082  0.8992039232588913  0.3622008934656729   T   T   T
 0.2017940928859721  0.4377098268082852  0.3656418759203069   T   T   T
 0.4405112962470920  0.9280390873845684  0.3728651163861156   T   T   T
 0.4117218274965824  0.3621882703700451  0.3742107598529510   T   T   T
 0.4512906643475875  0.9782342515209501  0.4391118058490404   T   T   T
 0.5171984744971135  0.2716376354479742  0.4323172265246225   T   T   T
 0.3071264950118950  0.4615648206182910  0.4186360039666155   T   T   T
 0.5172653797524736  0.9377526623757851  0.4589742162961846   T   T   T
 0.3621983674396231  0.9338168982801269  0.4575895575595122   T   T   T
 0.6036597043691518  0.3274646862626114  0.4190959777141451   T   T   T
 0.2791390130901077  0.3596347746178902  0.4444862869885375   T   T   T
 0.3782206968869056  0.7080784195053543  0.4204524064794805   T   T   T
```

*CH_2OH to *CH_2

11.5560998917000006 0.0000000000000000 0.0000000000000000
-2.8890500068999998 5.0039813974999996 0.0000000000000000
0.0000000000000000 0.0000000000000000 32.0765991211000028
Ag Cu C O H
24 4 1 2 5
Selective dynamics
Direct
0.0000000000000000 0.0000000000000000 0.1558800106620666 F F F
0.0000000048476740 0.5000000192346832 0.1558800106620666 F F F
0.2500000000064873 0.0000000000000000 0.1558800106620666 F F F
0.2500000047676068 0.5000000192346832 0.1558800106620666 F F F
0.5000000000129815 0.0000000000000000 0.1558800106620666 F F F
0.4999999841789133 0.5000000192346832 0.1558800106620666 F F F
0.7500000206146550 0.0000000000000000 0.1558800106620666 F F F
0.7500000460574441 0.5000000192346832 0.1558800106620666 F F F
0.0796729263828485 0.3219652620643996 0.2299517977619832 T T T
0.0814362426479632 0.8351888637101316 0.2300917285900327 T T T
0.3351550061430688 0.3600121898596305 0.2287475589621566 T T T
0.3401775894747135 0.8603263498973576 0.2291614159659570 T T T
0.6040145085454582 0.3280881907178478 0.2320467611250381 T T T
0.6002023777105353 0.8216379457827762 0.2336684160706951 T T T
0.8456110841932055 0.3320943795160591 0.2432571268597125 T T T
0.8405306985048500 0.8259699273725380 0.2476850600507341 T T T
0.1703187513967669 0.1765755764235458 0.3075692728921245 T T T
0.1624339128042956 0.6621156218646654 0.2990797784485664 T T T
0.4313932468875141 0.2298710615012127 0.2976379426406546 T T T
0.4333920405677211 0.7246269151671294 0.3027066404535875 T T T
0.6871409983178088 0.1868850553150161 0.3179981077502310 T T T
0.6719793199698701 0.6706331108781094 0.3261816208027341 T T T
0.9341093783015713 0.1817893659216496 0.3259548850894991 T T T
0.9377788812848149 0.6884011749702277 0.3266390218494126 T T T
0.2452041116980938 0.9680654208921512 0.3664193292381648 T T T
0.2442623653386702 0.5391883538478821 0.3672864164413631 T T T
0.4757988658426756 1.0515773475584587 0.3653266226788388 T T T
0.4363592011105592 0.4453566690877372 0.3707675307872568 T T T
0.3303439972681365 0.8449349390560461 0.4116821577956019 T T T
0.3601747648361301 0.5483743239287728 0.4196929736428527 T T T
0.2225309637002888 0.1916923161398855 0.4837006975801822 T T T
0.2650167251516662 0.8059992633738813 0.4390028253261924 T T T
0.4333082262848048 0.9744256120654179 0.4243292058135444 T T T
0.1662711799174517 0.0240354079150568 0.4690210830725131 T T T
0.2974615111949935 0.1760566230457033 0.4942129756617244 T T T
0.3015552841117124 0.4228078675582379 0.4417151939241233 T T T

*CH to *CH_2
11.5560998917000006 0.0000000000000000 0.0000000000000000
-2.8890500068999998 5.0039813974999996 0.0000000000000000
0.0000000000000000 0.0000000000000000 32.0765991211000028
Ag Cu C O H
24 4 1 1 3
Selective dynamics
Direct
0.0000000000000000 0.0000000000000000 0.1558800108193551 F F F
0.0000000049185545 0.5000000191254445 0.1558800108193551 F F F
0.2500000000034603 0.0000000000000000 0.1558800108193551 F F F

0.2500000048757798 0.5000000191254445 0.1558800108193551 F F F
0.5000000000069420 0.0000000000000000 0.1558800108193551 F F F
0.4999999840956377 0.5000000191254445 0.1558800108193551 F F F
0.7500000207940190 0.0000000000000000 0.1558800108193551 F F F
0.7500000460307347 0.5000000191254445 0.1558800108193551 F F F
0.0768552901459301 0.3337017412938502 0.2288428146045161 T T T
0.0761866530946116 0.8375777070848901 0.2281310906862460 T T T
0.3324087804205869 0.3338503315127062 0.2284428265476010 T T T
0.3344663488829203 0.8397477406143549 0.2287025868543507 T T T
0.5923684617702774 0.3550167164817197 0.2343310300024782 T T T
0.5958619616302944 0.8582904112679964 0.2306149411219317 T T T
0.8379064010063986 0.3475914510947545 0.2438855337209137 T T T
0.8359529956158210 0.8465647828132621 0.2438278847729565 T T T
0.1558490699708082 0.1714161302574339 0.3019393354257806 T T T
0.1505059166101843 0.6673685728088966 0.2988758764916759 T T T
0.4140476239595328 0.1711609023556436 0.3034367499125507 T T T
0.4191263146836859 0.6698992396284652 0.2980351196890000 T T T
0.6620448049604372 0.1723562851219804 0.3187026475840810 T T T
0.6608393721272292 0.6755289024431359 0.3204028424281433 T T T
0.9195765290602114 0.1818124218826299 0.3226272651595950 T T T
0.9143041878058417 0.6798845465547612 0.3204532013103086 T T T
0.1944987664319148 -0.0474408982504140 0.3673350281047357 T T T
0.2178716426361251 0.4971668425968030 0.3654588112152218 T T T
0.4453221645065322 0.9119484613820750 0.3678893983082578 T T T
0.4299744231559681 0.4702425297406540 0.3699583278153107 T T T
0.2846594773908768 0.8125963720619026 0.4012254810426198 T T T
0.4498358633138834 0.3228604744345437 0.4218227186220121 T T T
0.2453750947680082 0.7019472818696655 0.4308093093912155 T T T
0.5410191613252138 0.3658129752969405 0.4286044777337512 T T T
0.3754346729349495 0.0703795180494097 0.4131582117630637 T T T

*CH_2 to *CH_3
11.5560998917000006 0.0000000000000000 0.0000000000000000
-2.8890500068999998 5.0039813974999996 0.0000000000000000
0.0000000000000000 0.0000000000000000 32.0765991211000028
Ag Cu C O H
24 4 1 1 4
Selective dynamics
Direct
0.0000000000000000 0.0000000000000000 0.1558800109999936 F F F
0.0000000050000047 0.5000000189999980 0.1558800109999936 F F F
0.2499999999999914 0.0000000000000000 0.1558800109999936 F F F
0.2500000049999968 0.5000000189999980 0.1558800109999936 F F F
0.4999999999999757 0.0000000000000000 0.1558800109999936 F F F
0.4999999839999744 0.5000000189999980 0.1558800109999936 F F F
0.7500000209999972 0.0000000000000000 0.1558800109999936 F F F
0.7500000254606888 0.5000000105163737 0.1558800109999936 F F F
0.0830649558998524 0.3675318472013460 0.2284733176247540 T T T
0.0838024156438690 0.8592508369857496 0.2294191499402502 T T T
0.3446357546940536 0.3616245905888198 0.2301835386304591 T T T
0.3412315266945795 0.8571979948258441 0.2299635227454707 T T T
0.6057242772210200 0.3770851051022744 0.2321551322631638 T T T
0.6016089785232045 0.8765943021352109 0.2342744927363282 T T T
0.8439605577108501 0.3682519653865154 0.2487210097136144 T T T
0.8489803530752024 0.8740369681621228 0.2422645007707675 T T T

0.1620052824145614 0.2135853755654235 0.2982910485526390 T T T
0.1715845308299868 0.7267293085963593 0.3028735375589313 T T T
0.4392763947627086 0.2126176189637610 0.3011891443550910 T T T
0.4269412795824264 0.7098717654584151 0.3041288396225770 T T T
0.6712009294951969 0.1966471301937953 0.3258027826230860 T T T
0.6898224308970741 0.7232381939953245 0.3154938247143264 T T T
0.9376028524639389 0.2353374765322876 0.3269261415925174 T T T
0.9411573517336733 0.7367125326411219 0.3288257282325791 T T T
0.2554477321872679 0.1001078878719585 0.3627337364548865 T T T
0.1886708330801595 0.4717421463563387 0.3672961793121449 T T T
0.4123125598356552 0.9443400900326044 0.3747122429218538 T T T
0.4834116931379231 0.5171831557074498 0.3675863847232692 T T T
0.3920408183416836 0.2382462329381002 0.4120962939654890 T T T
0.3104602587067032 0.6009386242109773 0.4186788629904348 T T T
0.3625296678098405 0.4673912325002744 0.4188921120318450 T T T
0.4852371779607189 0.2680882773710005 0.4270732488833387 T T T
0.3118782357789149 0.1272326366119972 0.4352373244036615 T T T
0.2872463976382097 0.6458917637826395 0.4456144854141921 T T T

*CH_3 to * + CH_4(*g*)
11.5560998917000006 0.0000000000000000 0.0000000000000000
-2.8890500068999998 5.0039813974999996 0.0000000000000000
0.0000000000000000 0.0000000000000000 32.0765991211000028
Ag Cu C O H
24 4 1 1 5
Selective dynamics
Direct
0.0000000000000000 0.0000000000000000 0.1558800106620666 F F F
0.0000000048476609 0.5000000192346832 0.1558800106620666 F F F
0.2500000000064873 0.0000000000000000 0.1558800106620666 F F F
0.2500000047676068 0.5000000192346832 0.1558800106620666 F F F
0.5000000000129815 0.0000000000000000 0.1558800106620666 F F F
0.4999999841789133 0.5000000192346832 0.1558800106620666 F F F
0.7500000206146550 0.0000000000000000 0.1558800106620666 F F F
0.7500000460574811 0.5000000192346832 0.1558800106620666 F F F
0.0752175943135951 0.3446856590133749 0.2278592760883690 T T T
0.0779404181525439 0.8398926242310590 0.2295244659104439 T T T
0.3358096563374967 0.3457737027214198 0.2290805348700072 T T T
0.3358473276415584 0.8419713302931551 0.2309053845501193 T T T
0.5958325978284212 0.3503553399151891 0.2332809530351941 T T T
0.5925247764184831 0.8475287885743598 0.2361465379046267 T T T
0.8362134518200326 0.3434371268054216 0.2459030525289791 T T T
0.8379412848101258 0.8458657592381665 0.2442233751700048 T T T
0.1442334516229220 0.1859000845397721 0.2985700874518725 T T T
0.1529924606225654 0.6851232510921268 0.3031670681860291 T T T
0.4197512358492267 0.1983396100155503 0.3018184677352057 T T T
0.4153569513969252 0.6809695290382782 0.3062864281789376 T T T
0.6552474665148754 0.1805012024134116 0.3251217504942429 T T T
0.6653341699080431 0.6898917409484625 0.3213037717525790 T T T
0.9155326163537061 0.1981511477298490 0.3248613861410014 T T T
0.9174864188178571 0.6957156546169573 0.3244402663372800 T T T
0.2217318138843105 0.0446261902696984 0.3624541096721315 T T T
0.2163729639066347 0.4762489512975858 0.3658394846830170 T T T
0.4153403735229466 0.9668132600883427 0.3701431838737940 T T T
0.4474103631941652 0.4402060073404804 0.3758476323105506 T T T

0.4586045543874180 0.4448359057624723 0.4405071694865376 T T T
0.3351573483029433 0.7124634323256991 0.4191869671763207 T T T
0.3703905762112187 0.3014175250649457 0.4585285365721196 T T T
0.5252310538315942 0.6159530804555756 0.4603660307981741 T T T
0.5161402784542706 0.3433101851062034 0.4334810284348631 T T T
0.3927679877369024 0.5988785763596449 0.4254459385133298 T T T
0.3060096428235192 0.7680340147063681 0.4440007202863855 T T T

Au-Cu surface

*CO to *CHO
11.5352001190000006 0.0000000000000000 0.0000000000000000
-2.8838000298000002 4.9948881704000003 0.0000000000000000
0.0000000000000000 0.0000000000000000 32.0638008118000002
Au Cu C O H
24 4 1 2 2
Selective dynamics
Direct
0.0000000000000000 0.0000000000000000 0.1559400000000025 F F F
0.0000000029999958 0.5000000140000015 0.1559400000000025 F F F
0.2499999999999999 0.0000000000000000 0.1559400000000025 F F F
0.2500000029999965 0.5000000140000015 0.1559400000000025 F F F
0.4999999999999999 0.0000000000000000 0.1559400000000025 F F F
0.4999999830000019 0.5000000140000015 0.1559400000000025 F F F
0.7499999589999999 0.0000000000000000 0.1559400000000025 F F F
0.7499999409999984 0.5000000140000015 0.1559400000000025 F F F
0.0667184395008428 0.3301882632575487 0.2292853879733025 T T T
0.0692519271517022 0.8272639967505870 0.2301015473216141 T T T
0.3404019293075800 0.3478253728606355 0.2364606397731747 T T T
0.3339597240436357 0.8377422470206571 0.2352011580767336 T T T
0.6041417306952733 0.3597108215931892 0.2282575996638876 T T T
0.6001769710247171 0.8586046463041000 0.2300346615177132 T T T
0.8312922918607705 0.3374916669033063 0.2544977608001743 T T T
0.8317758786873612 0.8387441848126044 0.2508979012813836 T T T
0.1430817646068298 0.1658453922985849 0.2995440812268492 T T T
0.1549291420663307 0.6709814855868445 0.3031493314707279 T T T
0.4737364404639641 0.2260867440693239 0.3005941504334018 T T T
0.4693944465385387 0.7220423444979281 0.3051073849818708 T T T
0.6976684087459522 0.2121473755140439 0.3343410073584587 T T T
0.7075599671943891 0.7250686306660370 0.3305961165847708 T T T
0.9408720975837277 0.2133002888893083 0.3400315417393804 T T T
0.9408756591170604 0.7048852978845682 0.3389196192370972 T T T
0.2720494890754022 0.0710841408635626 0.3535626562122081 T T T
0.2586884825407064 0.4989499216554665 0.3595846460232675 T T T
0.4607194689853139 0.9962647610612155 0.3707461361601356 T T T
0.4883261520807176 0.4625010159967519 0.3717198780046670 T T T
0.4826878511642766 0.5040864883982651 0.4310149662220739 T T T
0.5296632854113876 0.4505123923873944 0.4597825726655995 T T T
0.3409701141786308 0.7211221878338693 0.4127692968929740 T T T
0.4109734361876630 0.6187850367643668 0.4292129509496588 T T T
0.2867597911557409 0.7629649486067661 0.4313275417119052 T T T

*CO to *COH
11.5352001190000006 0.0000000000000000 0.0000000000000000

-2.8838000298000002 4.9948881704000003 0.0000000000000000
0.0000000000000000 0.0000000000000000 32.0638008118000002
Au Cu C O H
24 4 1 2 2
Selective dynamics
Direct
0.0000000000000000 0.0000000000000000 0.1558800106620666 F F F
0.0000000048476560 0.5000000192346833 0.1558800106620666 F F F
0.2500000000064873 0.0000000000000000 0.1558800106620666 F F F
0.2500000047676068 0.5000000192346833 0.1558800106620666 F F F
0.5000000000129815 0.0000000000000000 0.1558800106620666 F F F
0.4999999841789132 0.5000000192346833 0.1558800106620666 F F F
0.7500000206146565 0.0000000000000000 0.1558800106620666 F F F
0.7500000460574725 0.5000000192346833 0.1558800106620666 F F F
0.0843859114284323 0.3331454558600924 0.2318119070894872 T T T
0.0840110324713958 0.8362224119852671 0.2318184949111243 T T T
0.3368896286892314 0.3401685589126449 0.2285860779348039 T T T
0.3378831258026862 0.8431281152461992 0.2287883726313197 T T T
0.6010997925651392 0.3458195122039331 0.2318905511567682 T T T
0.6034391909094732 0.8480940011035489 0.2312904038197620 T T T
0.8432943526726198 0.3409994114526156 0.2441001620480649 T T T
0.8412661358921043 0.8383281898797993 0.2455782393357224 T T T
0.1640010336267072 0.1636653435551226 0.3067783457370740 T T T
0.1646552754947039 0.6650587911079235 0.3053572963496506 T T T
0.4243663479679756 0.1839008233255928 0.2999758277651519 T T T
0.4276712039703640 0.6832769456774646 0.2980150211225897 T T T
0.6741789215932951 0.1723733012373022 0.3194278609027809 T T T
0.6690311031307850 0.6670593523993887 0.3230550117744521 T T T
0.9213427794115672 0.1698691643609060 0.3231608711059977 T T T
0.9209162234602727 0.6695766745638229 0.3228732610638791 T T T
0.2178766296448073 0.9832284879324229 0.3741461431039050 T T T
0.2189499598076207 0.4639873995308373 0.3718675727888598 T T T
0.4464458049402042 0.9571364379637379 0.3650601043135115 T T T
0.4393855605517393 0.4253426564598096 0.3697015702795208 T T T
0.4004333673294566 0.6527930605330184 0.4025283613548472 T T T
0.3803448358299955 0.6857477128281099 0.4411844265266026 T T T
0.1759754732417347 0.6882770008651726 0.4148064847989903 T T T
0.2982911521212555 0.7206740234488077 0.4404619018752117 T T T
0.0845204068051969 0.6011032625339155 0.4256316091722153 T T T

*CHO to *CHOH
11.5352001190000006 0.0000000000000000 0.0000000000000000
-2.8838000298000002 4.9948881704000003 0.0000000000000000
0.0000000000000000 0.0000000000000000 32.0638008118000002
Au Cu C O H
24 4 1 2 3
Selective dynamics
Direct
0.0000000000000000 0.0000000000000000 0.1559400000000026 F F F
0.0000000029999825 0.5000000140000015 0.1559400000000026 F F F
0.2499999999999999 0.0000000000000000 0.1559400000000026 F F F
0.2500000029999965 0.5000000140000015 0.1559400000000026 F F F
0.4999999999999999 0.0000000000000000 0.1559400000000026 F F F
0.4999999830000019 0.5000000140000015 0.1559400000000026 F F F
0.7499999589999999 0.0000000000000000 0.1559400000000026 F F F

0.7499999409999984	0.5000000140000015	0.1559400000000026	F	F	F
0.0283324052891180	0.3124829754161643	0.2318871279924233	T	T	T
0.0278303241781329	0.8131624331129624	0.2298661664519111	T	T	T
0.2961778314494394	0.4633736974310217	0.2456368158208778	T	T	T
0.2956742202009658	0.9614248319561234	0.2441557208841672	T	T	T
0.5862669005441129	0.3400766484072907	0.2268390303859408	T	T	T
0.5770222392564488	0.8351546732492471	0.2288823210512164	T	T	T
0.8053953286185024	0.3304847871707134	0.2606183875276040	T	T	T
0.8034371624608578	0.8291858641792215	0.2566777118918000	T	T	T
0.0952614243185164	0.2154818208491575	0.3090503977766845	T	T	T
0.0802857735936968	0.6869290450301009	0.3047803368177043	T	T	T
0.4709265014251456	0.3804555676931188	0.2991364463523339	T	T	T
0.4653925812444822	0.8753885564518814	0.3018677029144189	T	T	T
0.6880863210002180	0.3135142479089522	0.3397597447196640	T	T	T
0.6875091445612970	0.8097478838284651	0.3364053102041448	T	T	T
0.9141985985108975	0.2799601415611323	0.3594284169769266	T	T	T
0.9180746594919164	0.7863291077354820	0.3576148249950559	T	T	T
0.2568744905098466	0.1081552650037152	0.3432480943635924	T	T	T
0.2546578171349829	0.6537582923692321	0.3491118590181633	T	T	T
0.4346080419364721	1.5725916035804295	0.3711216270761172	T	T	T
0.4508142116250831	1.1116165941691354	0.3707841702959202	T	T	T
0.4781013830008170	1.8485057454151408	0.4148798646788724	T	T	T
0.4273833234903850	1.9385572445099941	0.4395245239498312	T	T	T
0.4701274677970033	2.4237575325372167	0.5175985064668522	T	T	T
0.5898966510744226	1.9188395455350509	0.4190554451268539	T	T	T
0.4579233388979075	2.2599570427484097	0.5050729119375743	T	T	T
0.5305190131402985	2.4548706452802911	0.5409770566516251	T	T	T

*CHO to *CH_2O

```
   11.5352001190000006    0.0000000000000000    0.0000000000000000
   -2.8838000298000002    4.9948881704000003    0.0000000000000000
    0.0000000000000000    0.0000000000000000   32.0638008118000002
  Au   Cu   C    O    H
  24   4    1    2    3
Selective dynamics
Direct
```

0.0000000000000000	0.0000000000000000	0.1558800106620666	F	F	F
0.0000000048476752	0.5000000192346832	0.1558800106620666	F	F	F
0.2500000000064873	0.0000000000000000	0.1558800106620666	F	F	F
0.2500000047676068	0.5000000192346832	0.1558800106620666	F	F	F
0.5000000000129815	0.0000000000000000	0.1558800106620666	F	F	F
0.4999999841789133	0.5000000192346832	0.1558800106620666	F	F	F
0.7500000206146550	0.0000000000000000	0.1558800106620666	F	F	F
0.7500000460574462	0.5000000192346832	0.1558800106620666	F	F	F
0.0597229250849467	0.3209226458480387	0.2304790429292224	T	T	T
0.0600091283331052	0.8250710555288713	0.2300729426811526	T	T	T
0.3434461461962696	0.2775510677542293	0.2302155526584863	T	T	T
0.3454516023943651	0.7758541351882229	0.2296925277357523	T	T	T
0.6137856944177051	0.3805050714483194	0.2312668854038279	T	T	T
0.6138947675874634	0.8800428130751173	0.2322745800998630	T	T	T
0.8353425510148704	0.3544644710421417	0.2586086631016214	T	T	T
0.8343302579038985	0.8532682394988476	0.2587294729417078	T	T	T
0.1775613088421195	0.1827819917258818	0.2988987096290871	T	T	T
0.1761545953754931	0.6869122072695760	0.2960029463394522	T	T	T
0.4488556530103202	0.1052626197706539	0.2991073193194043	T	T	T

0.4491161089485929 0.6013916395515295 0.3012894737118610 T T T
0.7010399482986914 0.3515393504746921 0.3377018033388686 T T T
0.6983198096314087 0.8541376397597699 0.3383925802497605 T T T
0.9474710694441169 0.3667657735793048 0.3411672241909623 T T T
0.9406146381181741 0.8598436144940691 0.3400294808834187 T T T
0.1677945986641692 0.9022898402790605 0.3656395359342689 T T T
0.1871523963054109 0.4588104631213336 0.3634289277215251 T T T
0.4928714191598554 0.9167378823802899 0.3658369838178404 T T T
0.4968561267628737 0.3813877193941034 0.3669807278849764 T T T
0.3915561103669095 0.2188930037987165 0.4168374386074106 T T T
0.2725905722279542 0.0129163061865646 0.4166555912892323 T T T
0.3443868812162520 0.6209000446725477 0.4035608117134638 T T T
0.4499954731843117 0.2268646844512769 0.4450895988272627 T T T
0.3821273713964276 0.4663160932947556 0.4079860048271812 T T T
0.3234014749949241 0.6854695074550959 0.4291576691073077 T T T

*COH to *C
11.5352001190000006 0.0000000000000000 0.0000000000000000
-2.8838000298000002 4.9948881704000003 0.0000000000000000
0.0000000000000000 0.0000000000000000 32.0638008118000002
Au Cu C O H
24 4 1 2 3
Selective dynamics
Direct
0.0000000000000000 0.0000000000000000 0.1559399996384713 F F F
0.0000000033847973 0.5000000135738748 0.1559399996384713 F F F
0.2500000000216716 0.0000000000000000 0.1559399996384713 F F F
0.2500000034064696 0.5000000135738748 0.1559399996384713 F F F
0.5000000000433431 0.0000000000000000 0.1559399996384713 F F F
0.4999999827089566 0.5000000135738748 0.1559399996384713 F F F
0.7499999586266384 0.0000000000000000 0.1559399996384713 F F F
0.7499999413789379 0.5000000135738748 0.1559399996384713 F F F
0.0643049320969929 0.3297799345885873 0.2298876876026124 T T T
0.0622826057993860 0.8249402054005904 0.2303906391229354 T T T
0.3336112228418021 0.2770226523336227 0.2319194477488547 T T T
0.3381698658738375 0.7857543414433975 0.2322325704398518 T T T
0.6040211922973071 0.3562797389870940 0.2319301512493723 T T T
0.6066856723957366 0.8608633431015712 0.2300495777148572 T T T
0.8317157475286611 0.3346613645706610 0.2567665089845995 T T T
0.8318066049280550 0.8329399174419432 0.2548606088071990 T T T
0.1622037371900197 0.2266945303353435 0.3007574209714472 T T T
0.1688585157323368 0.7332550280273272 0.3041160733449521 T T T
0.4477650969285620 0.1145246977814687 0.3024418578785993 T T T
0.4545775545275801 0.6223967903941039 0.2990572183412063 T T T
0.6966814450527135 0.2759279983820812 0.3379301605388592 T T T
0.7134320296648783 0.7952051147495757 0.3333582080418024 T T T
0.9513261363921830 0.2770893361346198 0.3364218545959870 T T T
0.9474644040895043 0.7623560764369117 0.3362062801629824 T T T
0.2675009128177321 0.1041573716285361 0.3625215199913439 T T T
0.2134026354179876 0.5215705142108872 0.3693945182106131 T T T
0.4697431832619787 0.8692779196504037 0.3686529551689393 T T T
0.5153870333417920 0.4052416028965952 0.3708101271619962 T T T
0.3771111785986259 0.4847728548015793 0.3722403860070125 T T T
0.2429166711930264 0.5984190424031387 0.4260736388848945 T T T
0.2801362682499305 0.8374880370883284 0.5000413534086852 T T T

0.2687789929722029 0.7786972912365384 0.4363414491738103 T T T
0.2473128093217709 0.6443059206452436 0.4979281345775995 T T T
0.3772031129411830 0.9278706935506119 0.4977026238188861 T T T

*COH to *CHOH
11.5352001190000006 0.0000000000000000 0.0000000000000000
-2.8838000298000002 4.9948881704000003 0.0000000000000000
0.0000000000000000 0.0000000000000000 32.0638008118000002
Au Cu C O H
24 4 1 2 3
Selective dynamics
Direct
0.0000000000000000 0.0000000000000000 0.1559399996384713 F F F
0.0000000033847949 0.5000000135738748 0.1559399996384713 F F F
0.2500000000216716 0.0000000000000000 0.1559399996384713 F F F
0.2500000034064696 0.5000000135738748 0.1559399996384713 F F F
0.5000000000433431 0.0000000000000000 0.1559399996384713 F F F
0.4999999827089566 0.5000000135738748 0.1559399996384713 F F F
0.7499999586266384 0.0000000000000000 0.1559399996384713 F F F
0.7499999413789379 0.5000000135738748 0.1559399996384713 F F F
0.0607381714820142 0.3175255646018397 0.2304432837833670 T T T
0.0586940554473370 0.8132862978395330 0.2302945894992576 T T T
0.3329399627163667 0.3563190857123883 0.2315847497521386 T T T
0.3166734451499453 0.8450685709347994 0.2330113837444453 T T T
0.5966103884712688 0.3453821414530597 0.2302771498274341 T T T
0.6075944965631811 0.8547412790228607 0.2270153961092428 T T T
0.8274408479386298 0.3285603540898579 0.2559152127752813 T T T
0.8265711378344903 0.8259703063929038 0.2565917853181481 T T T
0.1511340224471931 0.1588655176964719 0.2995817291229328 T T T
0.1502836227606155 0.6591060183240246 0.3055040656701388 T T T
0.4409296739747116 0.2264266170563558 0.3025034159778784 T T T
0.4563344109663010 0.7492495925509244 0.2947216487178152 T T T
0.6819568671285910 0.1934629733212418 0.3319989541218243 T T T
0.6728025724247284 0.6864687163682930 0.3323599397769212 T T T
0.9271648000312054 0.1828645657084972 0.3355219935827298 T T T
0.9213704443064048 0.6837818165856535 0.3333127982129103 T T T
0.2295000974845183 1.0279446515053317 0.3650843866113765 T T T
0.1895120287484653 0.4171275847184246 0.3688628841901466 T T T
0.4453289582703916 0.9581676207964439 0.3666820973115295 T T T
0.4253818632433405 0.5004560669172563 0.3639135788448414 T T T
0.3498749981202305 0.6419594537060985 0.4050383796848328 T T T
0.3879119745329367 0.6252445986634749 0.4454525930790934 T T T
0.2094909718169927 0.8818242913989353 0.4199697936624306 T T T
0.4743684433374890 0.6323238366168058 0.4453085537395389 T T T
0.2670973334579826 0.7343865530691838 0.4128032412285523 T T T
0.2519088996351149 0.0138746002544924 0.4421915874207665 T T T

*CH_2O to *OCH_3
11.5352001190000006 0.0000000000000000 0.0000000000000000
-2.8838000298000002 4.9948881704000003 0.0000000000000000
0.0000000000000000 0.0000000000000000 32.0638008118000002
Au Cu C O H
24 4 1 2 4
Selective dynamics
Direct

0.0000000000000000	0.0000000000000000	0.1558800106620666	F	F	F
0.0000000048476546	0.5000000192346833	0.1558800106620666	F	F	F
0.2500000000064873	0.0000000000000000	0.1558800106620666	F	F	F
0.2500000047676068	0.5000000192346833	0.1558800106620666	F	F	F
0.5000000000129815	0.0000000000000000	0.1558800106620666	F	F	F
0.4999999841789132	0.5000000192346833	0.1558800106620666	F	F	F
0.7500000206146565	0.0000000000000000	0.1558800106620666	F	F	F
0.7500000460574866	0.5000000192346833	0.1558800106620666	F	F	F
0.0656384414361273	0.3286123423463709	0.2295702115003541	T	T	T
0.0653608987357868	0.8254153373922433	0.2288350765770200	T	T	T
0.3295268978042601	0.3158749020241382	0.2321174798027972	T	T	T
0.3317161481867689	0.8180713440213480	0.2319400939129761	T	T	T
0.5890294217344337	0.3370145254046729	0.2307148229136978	T	T	T
0.5873149433043968	0.8403348922812052	0.2333713033131987	T	T	T
0.8248604611072288	0.3300811025427850	0.2531412133561729	T	T	T
0.8250973790205820	0.8310520379461348	0.2388068179282067	T	T	T
0.1454204433193836	0.1688060819950296	0.3009822598234506	T	T	T
0.1455333766746913	0.6772360687562474	0.2997353629940013	T	T	T
0.4387737855006459	0.1580638382308251	0.3029233945198031	T	T	T
0.4225856208213937	0.6403119977288039	0.3050420311252973	T	T	T
0.6811591526313174	0.2085196685694263	0.3311109847540129	T	T	T
0.6835513596469741	0.7168914161348723	0.3196764982830395	T	T	T
0.9232867504778514	0.2018249137997936	0.3321202857557115	T	T	T
0.9207989635852953	0.7027488775014199	0.3304799004095400	T	T	T
0.2510318277210190	1.0091088779709076	0.3612054415014030	T	T	T
0.2219585363680812	0.5203096487416065	0.3609282590512529	T	T	T
0.4494834634204681	0.9368596028088026	0.3727996590993409	T	T	T
0.4789832634801449	0.4263991015825971	0.3703348462423123	T	T	T
0.4173861517883186	0.5988075684159289	0.4497098235513674	T	T	T
0.4725794975308239	0.6806263678978192	0.4125709831450118	T	T	T
0.1884630142871575	0.2399222181038916	0.4083560089450384	T	T	T
0.4378423645115915	0.4576110599877652	0.4666525487720057	T	T	T
0.3866805531048154	0.7244611927081447	0.4667533569512460	T	T	T
0.0961575863477865	0.1511687339968456	0.4187478060820403	T	T	T
0.2548395873712133	0.3414303045678905	0.4322447488158123	T	T	T

*CHOH to *CH_2OH

11.5352001190000006	0.0000000000000000	0.0000000000000000
-2.8838000298000002	4.9948881704000003	0.0000000000000000
0.0000000000000000	0.0000000000000000	32.0638008118000002

Au Cu C O H
24 4 1 1 3
Selective dynamics
Direct

0.0000000000000000	0.0000000000000000	0.1558800106620666	F	F	F
0.0000000048476546	0.5000000192346833	0.1558800106620666	F	F	F
0.2500000000064873	0.0000000000000000	0.1558800106620666	F	F	F
0.2500000047676068	0.5000000192346833	0.1558800106620666	F	F	F
0.5000000000129815	0.0000000000000000	0.1558800106620666	F	F	F
0.4999999841789132	0.5000000192346833	0.1558800106620666	F	F	F
0.7500000206146565	0.0000000000000000	0.1558800106620666	F	F	F
0.7500000460574937	0.5000000192346833	0.1558800106620666	F	F	F
0.0645582406322923	0.3030806464693802	0.2299557119824679	T	T	T
0.0652219928577574	0.8166064899786267	0.2305275749125014	T	T	T
0.3240039151070935	0.3331842683167257	0.2334381243582835	T	T	T

0.3246809751443541 0.8316955465892572 0.2337169415323735 T T T
0.5911101058114253 0.3437732106286572 0.2286067308485639 T T T
0.5878310330853049 0.8331236851306835 0.2294179543318031 T T T
0.8253253351062049 0.3221258239508551 0.2375629338198546 T T T
0.8255234628679367 0.8232125337867716 0.2540434567838309 T T T
0.1509299444556513 0.1314043519474364 0.3060830474560247 T T T
0.1301032444429177 0.6168304224549019 0.3003739783608578 T T T
0.4444283059777799 0.2082868612149240 0.3003849704494364 T T T
0.4464323242411011 0.7112937584874119 0.3026092595696107 T T T
0.6906644913896905 0.1879227692274046 0.3199423140089033 T T T
0.6749340568891284 0.6710534588932719 0.3303739735630746 T T T
0.9186942396203115 0.1536536536835726 0.3326499806785158 T T T
0.9198930041386675 0.6662637727150700 0.3338844587962275 T T T
0.2559435843341303 0.9530078108107615 0.3606024336100010 T T T
0.2467187186803135 0.5127632714459397 0.3595947060949181 T T T
0.4914662190618897 0.0405638764267453 0.3695720734455456 T T T
0.4388141487155371 0.4377495650382791 0.3705037817009252 T T T
0.4423477418557740 0.6099121587109965 0.4254472758322659 T T T
0.4310488129106471 0.8367978859147229 0.4242843057290736 T T T
0.3708952162950074 0.4562273632400993 0.4467729817598504 T T T
0.5477345905040079 0.6622637204228856 0.4304903444380144 T T T
0.3297082735839455 0.8147055930678418 0.3974977022406868 T T T

*CHOH to *CH
11.5352001190000006 0.0000000000000000 0.0000000000000000
-2.8838000298000002 4.9948881704000003 0.0000000000000000
0.0000000000000000 0.0000000000000000 32.0638008118000002
Au Cu C O H
24 4 1 2 4
Selective dynamics
Direct
0.0000000000000000 0.0000000000000000 0.1559400000000014 F F F
0.0000000029999966 0.5000000140000015 0.1559400000000014 F F F
0.2499999999999999 0.0000000000000000 0.1559400000000014 F F F
0.2500000029999965 0.5000000140000015 0.1559400000000014 F F F
0.4999999999999999 0.0000000000000000 0.1559400000000014 F F F
0.4999999830000019 0.5000000140000015 0.1559400000000014 F F F
0.7499999589999999 0.0000000000000000 0.1559400000000014 F F F
0.7499999409999984 0.5000000140000015 0.1559400000000014 F F F
0.0645694007854469 0.3122958859698072 0.2291440944066539 T T T
0.0601073460544336 0.8035762418897870 0.2291035803999694 T T T
0.3463416626200243 0.3703843961570575 0.2334199277872295 T T T
0.3306465895450740 0.8586474384199637 0.2335635036692581 T T T
0.6015256934231182 0.3433395125854436 0.2308588011417361 T T T
0.6124778533030673 0.8523971859821992 0.2277654939553956 T T T
0.8308456920114633 0.3225357798719337 0.2567548259285929 T T T
0.8306499460356460 0.8189904450640159 0.2577500066388261 T T T
0.1565201523419246 0.1528654433837656 0.2970558270892974 T T T
0.1561402413601290 0.6519437829375138 0.3021797128206382 T T T
0.4533001573807382 0.2244794355022657 0.3051843476601993 T T T
0.4695647831308515 0.7525278875712157 0.2973987772141521 T T T
0.6887670348215433 0.2131325777946049 0.3343000646811135 T T T
0.6811447033914586 0.6992702447819610 0.3343724642475088 T T T
0.9291721017906074 0.1890349506734010 0.3346536576025025 T T T
0.9365440799811622 0.7024666731120646 0.3372723795968151 T T T

0.1986682585624623 0.9779019889239740 0.3648898416256125 T T T
0.1727809051841029 0.3874733030352693 0.3671009246906607 T T T
0.4483522977571138 0.9332726570770570 0.3698625851943191 T T T
0.4233150381061582 0.4808192441162778 0.3654836697243392 T T T
0.3246730901107512 0.5952327925016341 0.4012967549858131 T T T
0.3644349471531675 0.9923899615965991 0.4911386642810202 T T T
0.2994879111670027 0.9063518142371335 0.4070779326618930 T T T
0.3324809189061665 0.5837284509725511 0.4354099027429811 T T T
0.4570989028982230 0.0325944142002185 0.4914684231266077 T T T
0.3661335216811378 0.1483853557818623 0.5034916622259314 T T T
0.3158538188858356 0.9507451815495049 0.4372016733799178 T T T

*CH_2OH to *$HOCH_3$
11.5352001190000006 0.0000000000000000 0.0000000000000000
-2.8838000298000002 4.9948881704000003 0.0000000000000000
0.0000000000000000 0.0000000000000000 32.0638008118000002
Au Cu C O H
24 4 1 2 5
Selective dynamics
Direct
0.0000000000000000 0.0000000000000000 0.1558800106620666 F F F
0.0000000048476535 0.5000000192346833 0.1558800106620666 F F F
0.2500000000064873 0.0000000000000000 0.1558800106620666 F F F
0.2500000047676068 0.5000000192346833 0.1558800106620666 F F F
0.5000000000129815 0.0000000000000000 0.1558800106620666 F F F
0.4999999841789132 0.5000000192346833 0.1558800106620666 F F F
0.7500000206146565 0.0000000000000000 0.1558800106620666 F F F
0.7500000460574937 0.5000000192346833 0.1558800106620666 F F F
0.0498572628654872 0.2902987958897765 0.2298258322811537 T T T
0.0495745784698583 0.7940199632730456 0.2307174751400763 T T T
0.3125662117657975 0.2754549421243850 0.2379881078144565 T T T
0.3141539973976584 0.7756827329935253 0.2388993999403237 T T T
0.5885115395956917 0.3435312335988595 0.2278069579265709 T T T
0.5833671517801441 0.8375174185784030 0.2296359480343966 T T T
0.8143665144650879 0.3172036332928963 0.2515233370992310 T T T
0.8135484076521505 0.8191152149138848 0.2527175541652097 T T T
0.1227409152726004 0.1080819354104941 0.3034614769848461 T T T
0.1149209283474201 0.5947758314792604 0.3011917678281682 T T T
0.4595440468344784 0.1858411946603230 0.3011279264815851 T T T
0.4501388337387241 0.6721714162436190 0.3046526701197924 T T T
0.6871276673515546 0.1605363971218876 0.3315631138092459 T T T
0.6821140545391802 0.6605725115272640 0.3326433822011565 T T T
0.9197503641748144 0.1450399343862397 0.3444589437443200 T T T
0.9189620358426008 0.6489104632784141 0.3438698362805782 T T T
0.2553299741702211 0.9707559711133298 0.3509449965571842 T T T
0.2373841212365183 0.4907395004177608 0.3576577793013922 T T T
0.4582232315181672 0.9567979220931524 0.3701600154226306 T T T
0.4501497513719008 0.4133972367150565 0.3733685750229949 T T T
0.4829082684747257 1.0292446948100813 0.4341782516313739 T T T
0.5547295354187776 0.3204692551528089 0.4249143448193421 T T T
0.3308951365438527 0.5001600904494601 0.4130054449855994 T T T
0.5512305539715484 0.9855087502025804 0.4522060239989111 T T T
0.4008560101031421 1.0029414835934694 0.4546929163965220 T T T
0.6386531392856307 0.3650029210968225 0.4102517998665949 T T T
0.2813103622007644 0.3907955040366020 0.4369801575157167 T T T

0.3995773496714766 0.7506794391211578 0.4165803018369974   T   T   T

*CH_2OH to *CH_2
11.5352001190000006   0.0000000000000000   0.0000000000000000
-2.8838000298000002   4.9948881704000003   0.0000000000000000
0.0000000000000000   0.0000000000000000   32.0638008118000002
Au   Cu   C   O   H
24   4   1   2   5
Selective dynamics
Direct
0.0000000000000000 0.0000000000000000 0.1559399996384743   F   F   F
0.0000000033847982 0.5000000135738748 0.1559399996384743   F   F   F
0.2500000000216716 0.0000000000000000 0.1559399996384743   F   F   F
0.2500000034064698 0.5000000135738748 0.1559399996384743   F   F   F
0.5000000000433432 0.0000000000000000 0.1559399996384743   F   F   F
0.4999999827089570 0.5000000135738748 0.1559399996384743   F   F   F
0.7499999586266385 0.0000000000000000 0.1559399996384743   F   F   F
0.7499999413789386 0.5000000135738748 0.1559399996384743   F   F   F
0.0686934353003542 0.3163994774817896 0.2310610001449388   T   T   T
0.0644614226019048 0.8096302875051747 0.2278193067316941   T   T   T
0.3434902864286639 0.2136399933974009 0.2386178979845271   T   T   T
0.3427251311312370 0.7133949813346732 0.2392452553237417   T   T   T
0.6158332747588126 0.3766224807319969 0.2296537839280846   T   T   T
0.6157366889263687 0.8787773056246431 0.2303300135223288   T   T   T
0.8396411249767108 0.3512677787101184 0.2578184618337270   T   T   T
0.8379759698088304 0.8513536716541352 0.2567572648561424   T   T   T
0.1549778215612480 0.0710781411590767 0.3006420260489976   T   T   T
0.1675527448516700 0.5808290398766895 0.3060517782039832   T   T   T
0.4901259541001729 0.1421931751530737 0.3024582029599279   T   T   T
0.4868253418218923 0.6357571662290169 0.3043834805007817   T   T   T
0.7082658138450032 0.1149608433903020 0.3387204380862822   T   T   T
0.7129786684183421 0.6226169072876927 0.3364134020201249   T   T   T
0.9522928679827219 0.1084467354552754 0.3418773734975053   T   T   T
0.9506323562554964 0.6035528060126920 0.3397732405907378   T   T   T
0.2693817682149137 0.9572680516505011 0.3586087652218262   T   T   T
0.2206808572088514 0.3513855526230986 0.3657312273304802   T   T   T
0.4619657651741349 0.8802317947513465 0.3695757119256536   T   T   T
0.4781745809505053 0.3580107778617367 0.3724632112142140   T   T   T
0.3996584730610940 0.3980187713077760 0.4240775396282747   T   T   T
0.3428988903555823 0.5791090495003092 0.4146730292016216   T   T   T
0.3496295327139037 0.7050739858234732 0.4961655258837701   T   T   T
0.4844928896366225 0.5104619070498386 0.4462425495190028   T   T   T
0.3213284986070812 0.2168702167980651 0.4395244786191166   T   T   T
0.3240880801207019 0.6429076215517440 0.4412820560580194   T   T   T
0.2788873000742540 0.5375603306618635 0.5081228704849894   T   T   T
0.4295209032319237 0.6897177837542524 0.4981652071075295   T   T   T

*CH to *CH_2
11.5352001190000006   0.0000000000000000   0.0000000000000000
-2.8838000298000002   4.9948881704000003   0.0000000000000000
0.0000000000000000   0.0000000000000000   32.0638008118000002
Au   Cu   C   O   H
24   4   1   1   3
Selective dynamics
Direct

0.0000000000000000	0.0000000000000000	0.1559400000000026	F	F	F
0.0000000029999957	0.5000000140000015	0.1559400000000026	F	F	F
0.2499999999999999	0.0000000000000000	0.1559400000000026	F	F	F
0.2500000029999965	0.5000000140000015	0.1559400000000026	F	F	F
0.4999999999999999	0.0000000000000000	0.1559400000000026	F	F	F
0.4999999830000019	0.5000000140000015	0.1559400000000026	F	F	F
0.7499999589999999	0.0000000000000000	0.1559400000000026	F	F	F
0.7499999409999984	0.5000000140000015	0.1559400000000026	F	F	F
0.0689053210359855	0.3278489410633137	0.2301661961539988	T	T	T
0.0671748559239574	0.8238101378510113	0.2298862604442828	T	T	T
0.3475975477266395	0.2399214330472729	0.2319929089625514	T	T	T
0.3471356456091247	0.7501450631446852	0.2329556447443076	T	T	T
0.6186004241629006	0.3803104392714576	0.2335995732117202	T	T	T
0.6216388109416446	0.8889936495247398	0.2304186442570676	T	T	T
0.8433943729467005	0.3513443685460585	0.2585494786728390	T	T	T
0.8412355435248560	0.8498946735193404	0.2586463368220872	T	T	T
0.1759611547185733	0.1805492171444295	0.2974874494514801	T	T	T
0.1789390232984926	0.6856253199614830	0.3015321772994727	T	T	T
0.4573261639534787	0.0937608822568265	0.3053979389922226	T	T	T
0.4677053750705079	0.6051370940475600	0.2959273649946117	T	T	T
0.7118991073547701	0.2978522894661660	0.3368231116524519	T	T	T
0.7100593318492067	0.7901069542584701	0.3379769752131120	T	T	T
0.9570475415190031	0.2808205608034869	0.3398539238295870	T	T	T
0.9552411938364372	0.7818597570798813	0.3393045702322722	T	T	T
0.2100863410754040	0.9858796710725182	0.3664741524510035	T	T	T
0.2113594722521721	0.4271633800348911	0.3660430124364770	T	T	T
0.5124207322015916	0.8716403636389142	0.3675432697199887	T	T	T
0.4490714006816581	0.3843657770465755	0.3666508831715775	T	T	T
0.3745184208404108	0.5726731946681544	0.3989586020856951	T	T	T
0.2840522453589937	0.9072844160174156	0.4149071425847857	T	T	T
0.3284838196008666	0.7451308117580672	0.4077064161248672	T	T	T
0.3865321734266093	0.5030363435781237	0.4300103602067457	T	T	T
0.2374109165069147	0.8802899589081651	0.4414315742714836	T	T	T

*CH_2 to *CH_3

11.5352001190000006   0.0000000000000000   0.0000000000000000
-2.8838000298000002   4.9948881704000003   0.0000000000000000
0.0000000000000000   0.0000000000000000   32.0638008118000002
Au Cu C O H
24 4 1 1 4
Selective dynamics
Direct

0.0000000000000000	0.0000000000000000	0.1559400000000026	F	F	F
0.0000000029999925	0.5000000140000015	0.1559400000000026	F	F	F
0.2499999999999999	0.0000000000000000	0.1559400000000026	F	F	F
0.2500000029999965	0.5000000140000015	0.1559400000000026	F	F	F
0.4999999999999999	0.0000000000000000	0.1559400000000026	F	F	F
0.4999999830000019	0.5000000140000015	0.1559400000000026	F	F	F
0.7499999589999999	0.0000000000000000	0.1559400000000026	F	F	F
0.7499999409999984	0.5000000140000015	0.1559400000000026	F	F	F
0.0574640877434940	0.3032728254400928	0.2300415493243608	T	T	T
0.0557141588063098	0.7983850454998187	0.2296734874280812	T	T	T
0.3243169339535119	0.2936982623587441	0.2342788200727281	T	T	T
0.3204316169686298	0.7905447073468433	0.2348870469955937	T	T	T
0.5996263749569392	0.3564805337419769	0.2279086164672750	T	T	T

```
  0.5996833069598531  0.8579417852972827  0.2281853866732081   T   T   T
  0.8230269357654117  0.3296351047917520  0.2566297601816551   T   T   T
  0.8230394814688206  0.8288402339811544  0.2554725242623153   T   T   T
  0.1344000803871892  0.1167258054632635  0.3003311956419936   T   T   T
  0.1399671528144773  0.6256954324328208  0.3033743628013907   T   T   T
  0.4544749706969035  0.1806755240859788  0.2991442575567636   T   T   T
  0.4567465583850858  0.6815794964726264  0.2994125955572696   T   T   T
  0.6749077657454531  0.1563671404877032  0.3339087237165087   T   T   T
  0.6762340147685190  0.6525673413518147  0.3334311171453228   T   T   T
  0.9153778098435598  0.1465730017641653  0.3341331172513765   T   T   T
  0.9203572910104247  0.6501668096829434  0.3375335386694861   T   T   T
  0.1938255142734714  0.9530518305353284  0.3668948071635387   T   T   T
  0.2479212198143257  0.4584117749620009  0.3571646120879298   T   T   T
  0.4272932581380033  0.9284862779931885  0.3660393042883691   T   T   T
  0.4320889081317656  0.3876656286267046  0.3681763004297846   T   T   T
  0.3730267691015418  0.6069703071632426  0.4077556211360869   T   T   T
  0.2420443028718875  0.8786357826175855  0.4212333701535214   T   T   T
  0.3196237233212053  0.7724787940439642  0.4160531589408730   T   T   T
  0.4788148326535125  0.6998393906857509  0.4181981107536953   T   T   T
  0.3103826234041236  0.4741309201760798  0.4331893944722233   T   T   T
  0.2847478640387571  1.0357396892581063  0.4393216658573530   T   T   T
```

*CH_3 to * + CH_4(*g*)

```
   11.5352001190000006    0.0000000000000000    0.0000000000000000
   -2.8838000298000002    4.9948881704000003    0.0000000000000000
    0.0000000000000000    0.0000000000000000   32.0638008118000002
   Au   Cu   C    O    H
   24   4    1    1    5
Selective dynamics
Direct
  0.0000000000000000  0.0000000000000000  0.1558800106620666   F   F   F
  0.0000000048476624  0.5000000192346833  0.1558800106620666   F   F   F
  0.2500000000064873  0.0000000000000000  0.1558800106620666   F   F   F
  0.2500000047676068  0.5000000192346833  0.1558800106620666   F   F   F
  0.5000000000129815  0.0000000000000000  0.1558800106620666   F   F   F
  0.4999999841789132  0.5000000192346833  0.1558800106620666   F   F   F
  0.7500000206146565  0.0000000000000000  0.1558800106620666   F   F   F
  0.7500000460574794  0.5000000192346833  0.1558800106620666   F   F   F
  0.0656732852702520  0.3369315248017536  0.2287921998297414   T   T   T
  0.0674254815973838  0.8326196282615093  0.2299825884959656   T   T   T
  0.3275573689574922  0.3422022128717061  0.2330327598894527   T   T   T
  0.3274068103969285  0.8408809311723502  0.2338579438205279   T   T   T
  0.5912496011855152  0.3501997120570636  0.2299350221863310   T   T   T
  0.5883075860608823  0.8502289579554692  0.2316195517567944   T   T   T
  0.8239989020293048  0.3355046598608971  0.2508291483033450   T   T   T
  0.8245218290621376  0.8375718734651776  0.2468382991760862   T   T   T
  0.1289118101173392  0.1682794979914714  0.3002553225446383   T   T   T
  0.1394086428512118  0.6711108056341244  0.3044307230935021   T   T   T
  0.4393030629913945  0.2112750265802959  0.3017300682221297   T   T   T
  0.4330156041385768  0.6955938774668851  0.3048886148003233   T   T   T
  0.6641775453730743  0.1913315259227871  0.3317438079562640   T   T   T
  0.6719791366325878  0.7033041429517861  0.3273496733622838   T   T   T
  0.9068348783461609  0.1941136537769789  0.3301860601074221   T   T   T
  0.9063201334973549  0.6873278255207549  0.3276836418782225   T   T   T
  0.2399839740231326  0.0542289324048450  0.3577056362579347   T   T   T
```

0.2299994469832759	0.4855495027008485	0.3619259429224475	T	T	T
0.4290065485654989	0.9707433532209927	0.3699029860614210	T	T	T
0.4588804200691902	0.4475678077210217	0.3750702599157353	T	T	T
0.4654756648641816	0.4439029243153571	0.4389683706267516	T	T	T
0.3353405666420627	0.7010729636820002	0.4166827838017981	T	T	T
0.3784742693359779	0.2994137305454813	0.4574320510409400	T	T	T
0.5296956748485867	0.6210360589163771	0.4582568814055646	T	T	T
0.5265849468601245	0.3479356551343543	0.4330661285885971	T	T	T
0.3928424523961810	0.5910519397989649	0.4233755762987181	T	T	T
0.2982409245046258	0.7438220021571275	0.4411085603914868	T	T	T

Supplementary References

1. Gaarenstroom, S. & Winograd, N. J. T. J. o. C. P. Initial and final state effects in the ESCA spectra of cadmium and silver oxides. *J. Chem. Phys.* **67,** 3500-3506 (1977).

2. Han, S. W., Kim, Y. & Kim, K. Dodecanethiol-Derivatized Au/Ag Bimetallic Nanoparticles: TEM, UV/VIS, XPS, and FTIR Analysis. *J. Colloid Interface Sci.* **208,** 272-278 (1998).

3. Biesinger, M. C.*, et al.* Quantitative chemical state XPS analysis of first row transition metals, oxides and hydroxides. *J. Phys.: Conf. Ser.* **100,** 012025 (2008).

4. Biesinger, M. C., Lau, L. W. M., Gerson, A. R. & Smart, R. S. C. Resolving surface chemical states in XPS analysis of first row transition metals, oxides and hydroxides: Sc, Ti, V, Cu and Zn. *Appl. Surf. Sci.* **257,** 887-898 (2010).

5. Kim, Y. G., Baricuatro, J. H., Javier, A., Gregoire, J. M. & Soriaga, M. P. The evolution of the polycrystalline copper surface, first to Cu(111) and then to Cu(100), at a fixed CO_2RR potential: a study by operando EC-STM. *Langmuir* **30,** 15053-15056 (2014).

6. Kim, Y.-G.*, et al.* Surface reconstruction of pure-Cu single-crystal electrodes under CO-reduction potentials in alkaline solutions: A study by seriatim ECSTM-DEMS. *J. Electroanal. Chem.* **780,** 290-295 (2016).
